# Trisubstituted 1,3,5-Triazines as Histamine H_4_ Receptor Antagonists with Promising Activity In Vivo

**DOI:** 10.3390/molecules28104199

**Published:** 2023-05-19

**Authors:** Agnieszka Olejarz-Maciej, Szczepan Mogilski, Tadeusz Karcz, Tobias Werner, Katarzyna Kamińska, Jarosław Kupczyk, Ewelina Honkisz-Orzechowska, Gniewomir Latacz, Holger Stark, Katarzyna Kieć-Kononowicz, Dorota Łażewska

**Affiliations:** 1 Department of Technology and Biotechnology of Drugs, Faculty of Pharmacy, Jagiellonian University Medical College in Kraków, Medyczna 9, 30-688 Kraków, Poland; agnieszka.olejarz@uj.edu.pl (A.O.-M.); t.karcz@uj.edu.pl (T.K.); ewelina.honkisz@uj.edu.pl (E.H.-O.); gniewomir.latacz@uj.edu.pl (G.L.); mfkonono@cyf-kr.edu.pl (K.K.-K.); 2 Department of Pharmacodynamics, Faculty of Pharmacy, Jagiellonian University Medical College in Kraków, Medyczna 9, 30-688 Kraków, Poland; szczepan.mogilski@uj.edu.pl; 3 Institute of Pharmaceutical and Medicinal Chemistry, Heinrich Heine University Düsseldorf, Universitätsstr. 1, 40225 Düsseldorf, Germany; t.werner@hhu.de (T.W.); stark@hhu.de (H.S.)

**Keywords:** histamine H_4_ receptor, biased signalling, anti-inflammatory activity, analgesic activity, antipruritic activity

## Abstract

Pain is a very unpleasant experience that makes life extremely uncomfortable. The histamine H_4_ receptor (H_4_R) is a promising target for the treatment of inflammatory and immune diseases, as well as pain. H_4_R ligands have demonstrated analgesic effects in a variety of pain models, including inflammatory pain. Continuing the search for active H_4_R ligands among the alkyl derivatives of 1,3,5-triazine, we obtained 19 new compounds in two series: acyclic (I) and aliphatic (II). In vitro pharmacological evaluation showed their variable affinity for H_4_R. The majority of compounds showed a moderate affinity for this receptor (K_i_ > 100 nM), while all compounds tested in ß-arrestin and cAMP assays showed antagonistic activity. The most promising, compound **6**, (4-(cyclopentylmethyl)-6-(4-methylpiperazin-1-yl)-1,3,5-triazin-2-amine; K_i_ = 63 nM) was selected for further in vitro evaluation: blood-brain barrier permeability (PAMPA assay; P_e_ = 12.26 × 10^−6^ cm/s) and toxicity tests (HepG2 and SH-5YSY cells; no toxicity up to 50 µM). Next, compound **6** tested in vivo in a carrageenan-induced inflammatory pain model showed anti-inflammatory and analgesic effects (strongest at 50 mg/kg i.p.). Furthermore, in a histamine- and chloroquine-induced pruritus model, compound **6** at a dose of 25 mg/kg i.p. and 50 mg/kg i.p., respectively, reduced the number of scratch bouts. Thus, compound **6** is a promising ligand for further studies.

## 1. Introduction

Histamine is an important biogenic amine and endogenous neurotransmitter that has a number of important functions in the body, including the mediation of inflammatory and allergic reactions, playing an important role in wakefulness or sleep, and involvement in the sensation of pain [1,2]. In the CNS, histamine has antinociceptive activity while it has nociceptive in the periphery [3]. Histamine acts through four histamine receptors (H_1_–H_4_) that differ in their location, roles, and sensitivity to endogenous agonists [1,2]. Histamine H_4_ receptor (H_4_R) is located mainly in cells and tissues related to inflammatory state, such as eosinophils, mast cells, monocytes, lymphocytes, and macrophages [2,4].

H_4_R plays a significant role in the immune response that influences the inflammation process. Activation of H_4_R induces chemotaxis, not only of mast cells, eosinophils, and dendritic cells [5,6], but also migration of regulatory T-cells [7] and microglia [8]. In human mast cells, H_4_R activation induces the release of inflammatory mediators, such as Th2 cytokines (IL-4 IL-5 IL-13), pro-inflammatory cytokines (IL-6, IL-1beta), immunoregulatory cytokine IL-10 and chemokines (IL-8, MCP-1) [9]. In some experiments, microglial H_4_R activation led to the production of pro-inflammatory mediators [10,11] but in other experiments inhibited LPS-induced IL-1β production [8]. Furthermore, H_4_R induces the secretion of IL-16 from CD4+ T cells [12] and increases the secretion of INF-γ and IL-4 from NK cells [13]. H_4_R expression was also found to change in the presence of immune mediators [14,15]. 

**Figure 1:**
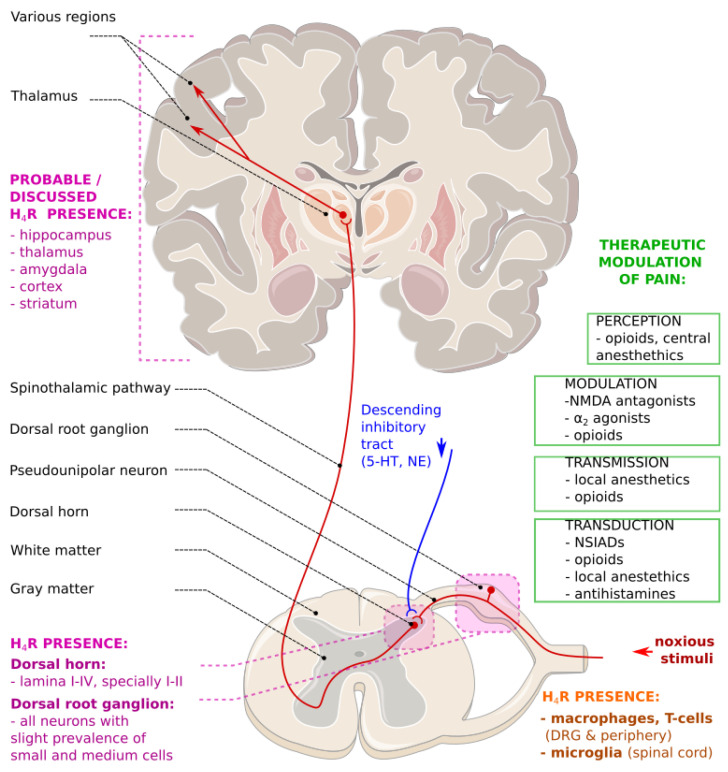
Simplified scheme showing pain pathway. The ascending pathway (red) is physiologically modulated by the descending inhibitory tracts (blue). Also represented are medications that can modify the sensory input of each of the four processes (green) [23,24]. Proposed H_4_R presence on neuronal cells in DRG, spinal cord [21,22] and in the brain [21,25] (pink) and non-neuron cells involved in pain [26] (orange). Abbreviations: 5-HT, serotonin, DRG, dorsal root ganglion; NE, norepinephrine; NMDA, N-methyl-d-aspartate receptor, NSAIDs, nonsteroidal anti-inflammatory drugs; Parts of Figure were drawn in Inkscape 1.1 using images from Servier Medical Art, which is licenced under a Creative Commons Attribution 3.0 Unported Licence (https://creativecommons.org/licenses/by/3.0/ accessed on 18 January 2023).

The presence of H_4_Rs in the CNS has been controversial and discussed by the research community in recent years [16,17,18,19]. Some reports indicate the presence of H_4_R on sensory nerves in the dorsal root ganglia and in the spinal cord [20,21,22]. The location of H_4_R coincides with the pathways of pain transmission (Figure 1) [23,24], supporting the modulatory role of H_4_R in this process [25,26]. 

**Figure 2:**
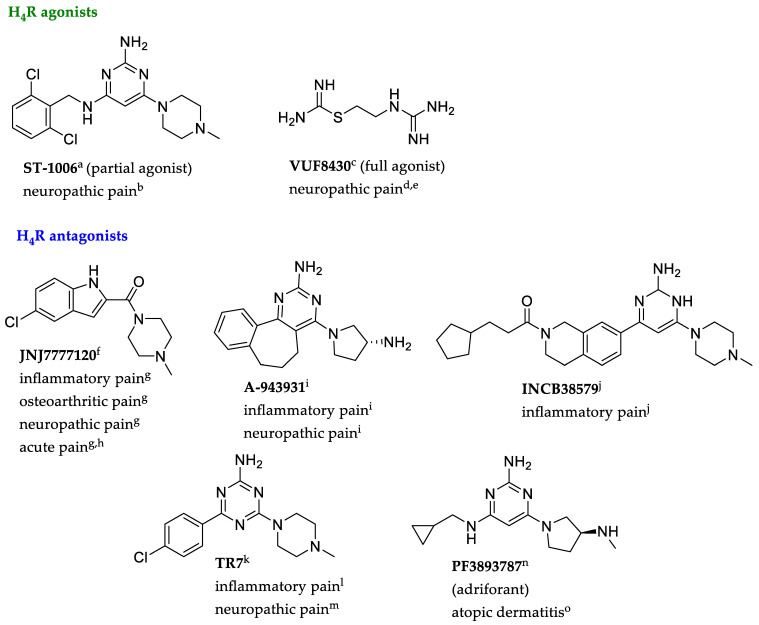
Structures of selected H_4_R ligands: agonists and antagonists with pharmacological activity in vivo. Information from ^a^ [41], ^b^ [22], ^c^ [42], ^d^ [28], ^e^ [29], ^f^ [43], ^g^ [31], ^h^ [32], ^i^ [33], ^j^ [34], ^k^ [44], ^l^ [35], ^m^ [36], ^n^ [45], ^o^ [39].

According to current knowledge, H_4_R possibly influences the pain process in two ways: reduces pain activity by neuronal H_4_R stimulation and promotes pain by pro-inflammatory effects by peripheral H_4_R stimulation [22]. Both H_4_R agonists [22,27,28,29] and antagonists [30,31,32,33,34,35,36,37,38] showed antinociceptive effects. H_4_R agonists induced analgesic activity when administered intrathecally [28] or intracerebroventricularly [22] which was performed to measure the effect that comes from the central, not peripheral, H_4_R. The H_4_R antagonists showed antinociceptive activity in many models of pain, including acute, chronic, inflammatory, neuropathic, postsurgical, and osteoarthritic pain [30,31,32,33,34,35,36,37,38]. The structures of the most interesting H_4_R ligands with antinociceptive activity in vivo are shown in Figure 2. For all presented antagonists, promising anti-inflammatory activity in in vivo models of inflammatory diseases were also observed e.g., pruritus (**INCB38579** [33]), peritonitis model (**JNJ7777120** [31], **TR7** [34], **A-987306** [37]]) or atopic dermatitis (e.g., **adriforant** Figure 2, [39]). Several of the most promising H_4_R ligands entered into clinical trials, e.g., **JNJ-39758979** (atopic dermatitis; trials terminated due to agranulocytosis [40], **toreforant** (rheumatoid arthritis; trials terminated due to lack of efficacy [40], or **adriforant** (atopic dermatitis; discontinued [39]). 

**JNJ7777120** (Figure 2), the first potent and selective H_4_R ligand [43], is the standard reference for both in vitro and in vivo studies. Numerous preclinical tests confirmed the high efficacy of this compound but also showed complicated pharmacological behaviour [40]. **JNJ7777120** was reported as neutral antagonist and inverse antagonist in G_αi_ dependent signalling [46,47,48], while acting as a partial agonist in the β-arrestin pathway [46,48,49]. Furthermore, **JNJ7777120** acted as an antagonist in in vivo studies and in primary cells [4].

**Figure 3:**
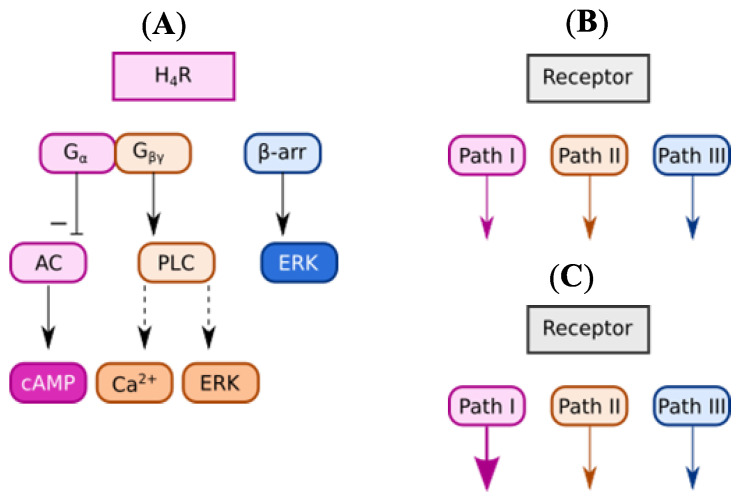
Schemes splintered of (**A**) signalling pathways after activation of H_4_R. Some second gauges were omitted for the purpose of clarity of the scheme. The dotted arrows indicate that there are more steps between the two levels that were skipped for the readability of the scheme. G-protein-dependent signal from H_4_R involves G_i/0_ protein. Activation of the H_4_R leads to inhibition of adenyl cyclase (AC) and its signalling pathway through G_α_ protein, activation of PLCβ through G_βγ_ protein which in turn leads to Ca^2+^ release and activation of ERK1/2 pathway [2,5,53,54,58] (**B**) bias signalling after binding of balanced ligand (**C**) bias signalling after binding of unbalanced ligand—bias towards Pathway I. Abbreviations: G_α_, α subunit of G-protein; G_βγ_, βγ subunit of G-protein; β-arr, β-arrestin; AC, adenyl cyclase; PLC, phospholipase C; ERK, protein–serum/threonine kinases; cAMP, cyclic adenosine monophosphate; Path—signalling pathway.

H_4_R is G protein-coupled receptor (G_i/o_) and upon stimulation activates a specific G-protein-dependent pathway and/or independent (β-arrestin) elements of the signal transduction cascade (Figure 3A) [50,51]. Ligands binding to this receptor may represent balanced activity towards all pathways (Figure 3B) or show preference toward one of them (Figure 3C) [52]. This unique behaviour is called functional selectivity or biased signalling.

Moreover, H_4_R signalling pathways depend on cell background. In recombinant systems, H_4_R activation inhibits adenylyl cyclase activity, resulting in a decrease in intracellular cAMP [53,54], while in some cell types (i.e., mouse mast cells), endogenous activation of H_4_R led to Ca^2+^ mobilization, without influence on cAMP (in G_i_ protein-dependent manner proved by pertussis toxin) [5]. Functional selectivity with one signalling branch is proposed to be responsible for therapeutic effects, while the other signalling could cause unwanted side effects [55]. Thus, from the point of view of drug screening, it is not sufficient to rely only on one functional assay [49]. In our previous studies, **TR7** (Figure 2) was shown to be an H_4_R antagonist in the cAMP accumulation assay of cAMP [56] and the Ca^2+^ efflux aequorin-based assay [57], while it showed agonist activity in the adhesion of eosinophils to the endothelium assay [57].

**Figure 4:**
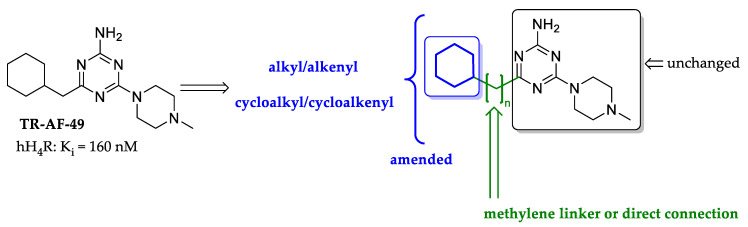
Design of novel histamine H_4_ receptor ligands.

The search for new compounds with biological activity can be inspired by compounds found in various types of extracts and natural products [59] or publications by other authors, which are especially useful in the search for effective ways to fight cancer or infection diseases, e.g., SARS-CoV2 [60]. Recently, we have published a series of compounds, alkyl derivatives of amine-1,3,5-triazine, which showed promising H_4_R in vitro and in vivo activity [61]. The most potent compound from those series, **TR-AF-49** (Figure 4) was chosen as a lead structure for further modifications and a new series of alkyl derivatives was designed (Figure 4), synthesized, and pharmacologically evaluated in vitro for human H_4_R (hH_4_R) affinities. Next, the complicated pharmacology of previously tested compounds (i.e., **TR7**) and the fact that H_4_R may represent the features of functional selectivity [62] encouraged us to expand the scope of the investigation to more than one signal transduction pathway (cAMP and ß-arrestin). Next, the most promising compound from the new series was evaluated for toxicity (in HepG2 and SH-SY5Y cells) and artificial membrane permeability (in PAMPA assay), and finally was tested in in vivo inflammatory pain models and pruritus models.

## 2. Results and Discussion

### 2.1. Design of Compounds

Based on our previous research results [61], **TR-AF-49** (Figure 4) with a good affinity for hH_4_R (K_i_ = 160 nM), was chosen as the lead structure. This compound proved to be an antagonist in functional tests (the cellular aequorin-based functional assay and [^35^S]GTPγS binding assay) and showed promising analgesic activity in inflammatory pain models (formalin test, carrageenan-induced inflammation). In the present work, we designed modifications to this structure: by changing the length of the alkyl chain (mainly elongation), introducing a double bond into the molecule and replacing the cyclohexane ring with other cyclic rings (cyclopropane, cyclobutane or cyclopentane) (Figure 2).

### 2.2. Synthesis of Compounds

**Scheme 1:**
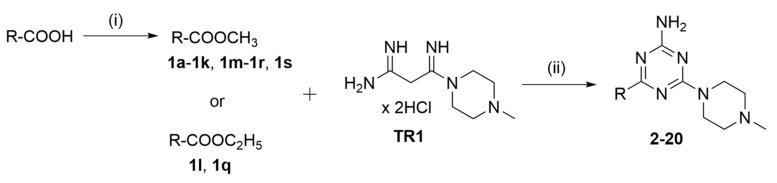
Synthetic route for target compounds **2**–**20**. Reagents and conditions (i) CH_3_OH, conc. H_2_SO_4_, rt 24 h; (ii) freshly prepared CH_3_ONa, rt 2-3 h; **TR1**, rt, 24–48 h. For R See Table 1 and Table 2.

Compounds were synthesized as shown in Scheme 1. Commercially unavailable esters (**1a**-**1i**, **1k**-**1l**, **1n**-**1p**, **1r**, and **1t**) were prepared from proper carboxylic acids to methyl esters by refluxing in methanol in the presence of sulfuric acid as described previously [61]. Next, crude and commercially available (**1j**, **1m**, **1q** and **1s**) esters were coupled with **TR1** (4-methylpiperazin-1-yl biguanide dihydrochloride) in a freshly prepared sodium methoxide as described previously to give desired 1,3,5-triazines **2**–**21** [44]. For all compounds, spectral analysis (^1^H NMR and ^13^C NMR) and mass spectrometry (LC/MS) confirmed their structures.

**Scheme 2:**
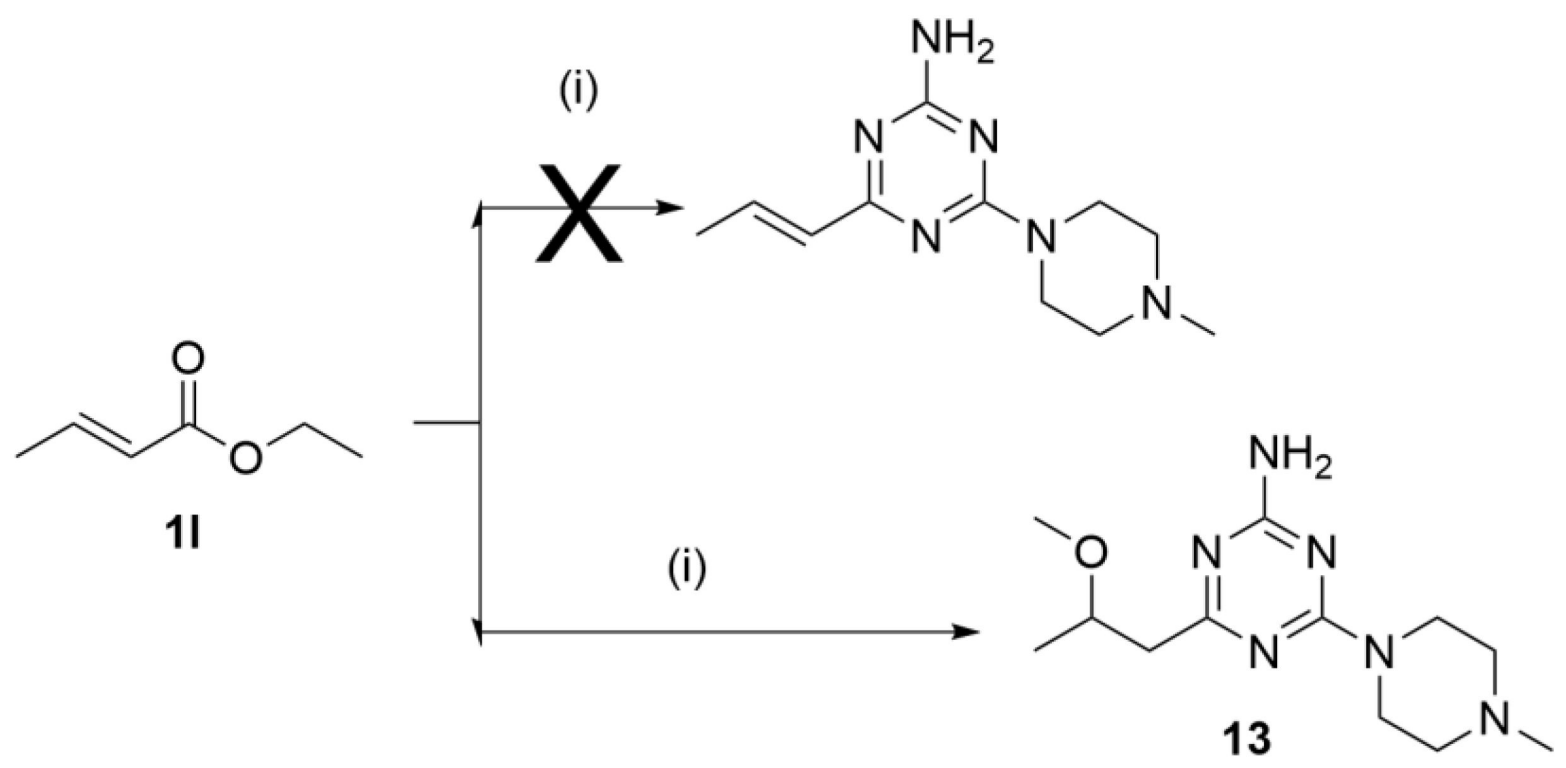
Synthesis of compound **13**. Reagents and condition: (i) freshly prepared CH_3_ONa, rt 2–3 h; **TR1**, rt, 48 h.

Compound **13** was obtained from commercially available ethyl crotonate (Alfa Aesar **1l**). However, the product proved to be not as expected 4-(4-methylpiperazin-1-yl)-6-(prop-1-enyl)-1,3,5-triazin-2-amine but 4-(2-methoxypropyl)-6-(4-methylpiperazin-1-yl)-1,3,5-triazin-2-amine (**13**) (Scheme 2). The reaction was repeated three times but the product was the same every time. In these cases, α,β-unsaturated esters reacted via the Michael addition reaction with sodium methoxide and produced β-methoxylated derivatives. Analysis of mass spectrometry confirmed the formation of this product with a parent ion [M + H]^+^ = 267.23 with one greater than the theoretical mass of M = 266.35 (Scheme 2). This molecular mass was 33 greater than the mass of the expected structure (Scheme 2). Further, ^1^H NMR and COSY NMR spectrum for compound **13** (Supplementary Materials) confirmed the formation of the predicted structure. A similar observation was made by Kisanga et al. [63] who also obtained ethyl 3-methoxybutanoate, in methanol but in the presence of the catalytic amount of the nonionic strong base proazaphosphatrane (P(*i*-BuNCH_2_CH_2_)_3_N), which further underwent in that condition transesterification.

**Table 1:** Structures and in vitro activity of tested alicyclic derivatives.

No	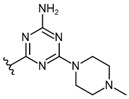	hH_4_R ^a^ K_i_ [nM] [CI 95%] (n) ^b^ or (inh. at 1 μM) ^c^	β-arrestin hH_4_R ^d^ IC_50_ ± SEM [nM] ^e^ (% of max. Antagonist Activity at 10 µM)	cAMP hH_4_R ^f^ IC_50_ ± SEM [nM] ^e^ (% of max. Antagonist Activity at 10 µM)
**2**	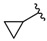	574 [160;2058] (3)	287 ± 30 (96)	847 ± 53 (99)
**3**	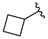	200 [156;257] (3)	100 ± 15 (87)	104 ± 17 (109)
**4**	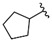	1237 [250;6115] (2)	68.8 ± 2 (95)	102 ± 2 (95)
**5**	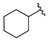	432 [128;1452] (3)	101 ± 10 (92)	219 ± 24 (98)
**6**	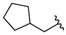	63 [18;214] (3)	10 ± 1 (101)	10 ± 2 (100)
**7**	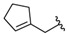	96 [20;450] (3)	14 ± 1 (103)	33 ± 2 (104)
**TR-AF-49** **(Lead)**	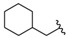	160 ^g^ [66.6;385] (4)	68 ± 7 (99)	271 ± 11 (98)
**8**	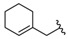	4700 ^h^ (1)	41 ± 6 (102)	743 ± 30 (98)
**9**	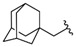	(43%) (2)	nt ^i^	nt ^i^
**JNJ7777120**		32 ^j^	56 ± 8 (91)	49 ± 2 (96)
**Thioperamide**		106 ^j^	209 ± 40 (102)	453 ± 30 (111)

^a^ [^3^H]histamine displacement assay with membrane preparation of S*f*9 cells expressing human histamine H_4_ receptor, co-expressed with G protein Gα_i2_ and Gβ_1_γ_2_ subunits [44]; ^b^ Mean values within 95% confidence intervals (CI), (n) number of performed experiments; ^c^ The per cent of inhibition at 1 μM, mean values of two independent experiments; ^d^ LiveBLAzerTM cell-based assay, ^e^ mean values of 2–5 independent experiments in triplicates ± SEM; ^f^ cAMP accumulation assay by LANCE Ultra cAMP detection; ^g^ data from Łażewska et al. [61]; ^h^ data from Grosicki et al. [57]; ^i^ nt: not tested; ^j^ data from Schneider et al. [64].

**Table 2:** Structures and in vitro activity of tested aliphatic derivatives.

No	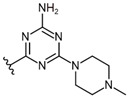	hH_4_R K_i_ [nM] ^a^ $\overset{-}{x}$ [CI 95%] (n) ^b^ or (inh. at 1 μM) ^c^	hH_4_R β-arrestin ^d^ IC_50_ ± SEM [nM] ^e^ (% of max. Antagonist Activity at 10 µM)	hH_4_R cAMP ^f^ IC_50_ ± SEM [nM] ^e^ (% of max. Antagonist Activity at 10 µM)
**10**	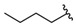	192 [42;874] (4)	82 ± 5 (96)	445 ± 10 (105)
**11**	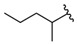	353 [275;454] (3)	97 ± 16 (97)	640 ± 49 (100)
**12**	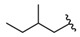	321 [127;814] (3)	38 ± 7 (100)	132 ± 9 (111)
**13**	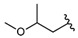	4264 [2074;8767] (2)	nt ^g^	nt ^g^
**14**	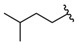	203 [69;601] (3)	24 ± 3 (101)	43 ± 17 (107)
**15**	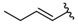	1490 [489;4538] (3)	nt ^g^	nt ^g^
**16**	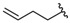	319 [223;458] (3)	146 ± 38 (94)	635 ± 123 (104)
**17**	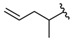	263 [112;617] (3)	744 ± 139 (92)	721 ± 70 (94)
**18**	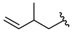	262 [91;756] (3)	66.6 ± 3 (101)	188 ± 45 (104)
**19**	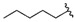	393 [224;688] (3)	34.9 ± 7 (99)	419 ± 77 (97)
**20**	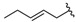	(6%) (3)	376 ± 9 (101)	nt ^g^

^a^ [^3^H]histamine displacement assay with membrane preparation of S*f*9 cells expressing human histamine H_4_ receptor, co-expressed with G protein Gα_i2_ and Gβ_1_γ_2_ subunits [44]; ^b^ mean values within 95% confidence intervals (CI), (n) number of performed experiments; ^c^ the percent of inhibition at 1 μM, mean values of at least two independent experiments; ^d^ LiveBLAzerTM cell-based assay; ^e^ mean values of 2–5 independent experiments in triplicates ± SEM; ^f^ cAMP accumulation assay by LANCE Ultra cAMP detection; ^g^ nt—not tested.

### 2.3. In Vitro Pharmacological Studies

#### 2.3.1. Histamine H_4_ Receptor Affinity

The affinity of compounds **2**–**20** for hH_4_R was evaluated in a binding assay as previously described [44]. [^3^H]Histamine was used as a radioligand and hH_4_R was expressed in S*f*9 cells with G protein Gα_i2_ and Gβ_1_γ_2_ subunits. The pharmacological results represented as K_i_ values are listed in Table 1 (for alicyclic derivatives) and Table 2 (for aliphatic derivatives). The compounds showed variable affinities for hH_4_R, ranging from good (K_i_ < 100 nM) to very weak (K_i_ > 4000 nM). Results depended on the substituent in the four positions of a triazine (a main structure).

In the group of alicyclic derivatives (**2**–**9**; Table 1), the affinity was related to the type of ring and its distance from the main structure. Compounds with 4- and 6-membered alicyclic moiety (**3**, **5**) had better affinity than 3-membered (**2**) opposite to the 5-membered (**4**), which showed a much weaker affinity. The introduction of a methylene linker between the alicyclic ring (5- and 6-membered) and the triazine scaffold increased the affinity for hH_4_R (**6** vs. **4** and **TR-AF-49** vs. **5**). The use of a larger adamantane ring resulted in a significant decrease in affinity (**9** vs. **TR-AF-49**). Additionally, the presence of an unsaturated bond in the rings (**7**, **8**) led to a lower affinity for these ligands. Among this group of alicyclic derivatives (**2**–**9**), two compounds (**6** and **7**) achieved higher binding affinity than the lead structure **TR-AF-49**.

In the group of aliphatic derivatives (**10**–**20**; Table 2), the affinity was influenced by the presence of a methyl branch and/or a double bond in the main chain. Unbranched saturated aliphatic derivatives (**10**, **19**) showed better affinity than branched compounds (**11**–**14**). An introduction of the methyl substituent into the butyl chain in the α- or ß- positions to the triazine ring resulted in a decrease in affinity (**10** vs. **11** vs. **12**). On the contrary, the presence of this substituent in the γ-position had little effect on affinity (**14** vs. **10**). The introduction of a double bond regardless of the position caused a decrease in affinity (**10** vs. **15** vs. **16** or **19** vs. **20**). In contrast, the presence of this bond in compounds with branching at the α- and ß-positions slightly increased receptor affinity (**17** vs. **11** and **18** vs. **12**). Thus, the presence of the double bond was profitable for the branched derivatives and unprofitable for straight-chain compounds.

The exchange of a carbon atom for an oxygen atom similar to the series described earlier [61] led to a significant decrease in affinity (**12** vs. **13**). In this group, none of the compounds achieved the high binding affinity of the lead structure **TR-AF-49**. In comparison with our previous work [61] where an elongation of the chain from methyl to propyl resulted in an increase in hH_4_R affinity (K_i_ from 2664 nM to 185 nM), present results showed that the butyl chain is optimal as further increasing to the pentyl one caused a decrease in affinity.

To sum up, the introduced modifications to the lead **TR-AF-49** gave the very potent compound **6** (K_i_ = 63 nM). This compound showed higher affinity than thioperamide but worse than **JNJ7777120** (K_i_ = 106 nM, and K_i_ = 32 nM, respectively [64]).

#### 2.3.2. Functional Characterization in β-Arrestin Recruitment Assay

β-Arrestin recruitment assay was performed only for the ligands that had K_i_ < 1 µM in the binding assay (except compound **3**: K_i_ = 1247 nM) (Table 1 and Table 2). Compounds were tested using LiveBLAzer^TM^ assay and Tango-H4-bla U2OS cells, allowing the estimation of the percentage of β-arrestin recruitment in response to tested treatments.

Compounds were tested in agonist and antagonist mode of the assay and all showed antagonistic properties.

For alicyclic derivatives (Table 1) we observed a similar structure–activity relationship as in the case of hH_4_R binding affinity evaluation: 4-, 5- and 6-membered alicyclic moieties showed better activity in the β-arrestin recruitment assay than 3-membered (**3**–**5** vs. **2**) compound. The presence of a methylene linker between the alicyclic ring and the triazine scaffold improved the activity (**6** vs. **4** and **TR-AF-49** vs. **5**). Addition of a double bond slightly improved activity only in the cyclohexylene derivative (compare **TR-AF-49** vs. **8**).

The 5-membered ring compounds, **6** and **7**, (without or with the double bond) most potently blocked the histamine-induced β-arrestin recruitment. In that particular assay conditions, these compounds performed better (IC_50_ < 15 nM) than **JNJ7777120** (IC_50_ = 56 nM).

For aliphatic derivatives (Table 2) addition of the methyl group in the β- or γ-position, or elongation of the carbon chain (from butyl to pentyl) resulted in more active antagonists (**12**, **14**, **19** vs. **10**). Presence of the unsaturated bond at the chain diminished the activity (**10** vs. **16**, **12** vs. **18**, **19** vs. **20**). The most active compound **14** with an IC_50_ of 24 nM also showed better activity than **JNJ7777120** (IC_50_ = 56 nM).

#### 2.3.3. Functional Characterization in cAMP Accumulation Assay

Intrinsic activity via the Gα subunit was measured using a LANCE Ultra cAMP detection kit and CHO-K1 cells stably expressing hH_4_R. Adenyl cyclase was stimulated by forskolin (10 µM). Histamine (140 nM) was used with the tested ligands in the antagonist mode of the assay. Only compounds with binding K_i_ values < 1 µM (except compound **3**: K_i_ = 1247 nM) were tested in this assay.

Among the alicyclic derivatives (**2**–**9**; Table 1) SAR of cAMP accumulation directly reflected the binding affinity (see Section 2.3.1).

Aliphatic derivatives (**10**–**20**; Table 2) showed a similar SAR pattern as compounds in the β-arrestin recruitment assay (see Section 2.3.2). The addition of methyl group in β- and γ-positions of the main chain increased activity, while the introduction of methyl substituent in α-position decreased antagonist activity (**12**, **14** and **11** vs. **10**). Elongation of the aliphatic chain had no influence on antagonist activity towards cAMP pathway (**10** vs. **20**).

The most active antagonist in both series, compound **6** (IC_50_ = 10 nM) showed better activity than **JNJ7777120** (IC_50_ = 49 nM) tested in the same conditions.

#### 2.3.4. Comparison of Intrinsic Activities

In the next step, we decided to compare the intrinsic activities of compounds toward two transduction pathways. Because both functional assays were conducted in different conditions, we first calculated K_b_ values using the Leff–Dougall variant of the Cheng–Prusoff equation (Equation (1)) [65].

(1) $$K_{b}={IC}_{50}/{{\left((2+([Ag]/[{EC}_{50}]\right)}^{n})}^{1/2}-1)$$

where: IC_50_, concentration of antagonist that inhibits agonist response by 50%; [Ag], concentration of agonist employed in the assay; [EC_50_], agonist EC_50_ value in the assay; n, Hill slope of the concentration–response curve of the agonist.

To compare the two pathways, we transformed the data to pK_b_ values and calculated the differences in pK_b_ values between the two pathways (Equation (2)).

(2) $$Bias\;factor=pK_{b\;\beta -arr}-{pK}_{b\;cAMP}$$

where: pK_b_ − log from K_b_ values; β-arr, β-arrestin pathway; cAMP, cAMP pathway. 

Calculated pK_b_ values and bias factors are presented in Table 3. A bias factor above 0 suggests a ligand bias towards the β-arrestin pathway while a bias factor below 0 suggests a ligand bias toward the cAMP pathway.

**Figure 5:**
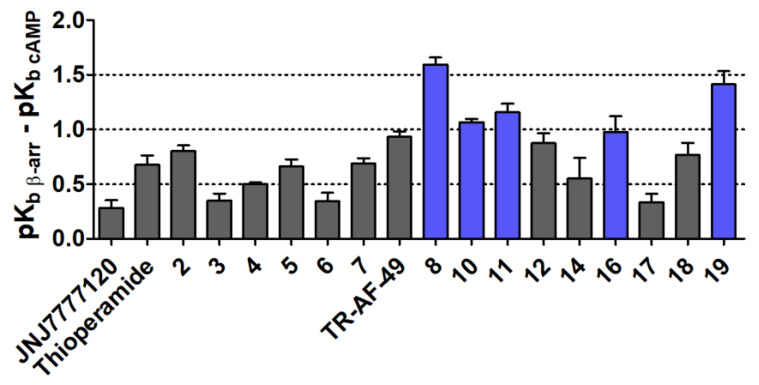
Bar plots representing bias factors (as differences between pK_b_ values between the signal transduction pathways) with SEM. Compounds with the highest differences (bias factor ≥ 1) are highlighted by blue bars.

All tested compounds showed bias towards the ß-arrestin pathway (Table 3, Figure 5).

All tested compounds showed bias towards the β-arrestin pathway (Table 3, Figure 5). In general, triazine derivatives with aliphatic moiety (**10**–**19**; Table 3, Figure 5) showed higher bias towards the β-arrestin pathway than those with alicyclic fragments (**2**–**8**; Table 3, Figure 1). The highest bias towards the β-arrestin pathway (bias factor ≥ 1) was observed for compounds **8**, **10**, **11**, **16**, and **19**, while the most balanced activity (bias factor ≤ 0.5) was determined for compounds **3**, **6** and **17**. Among alicyclic derivatives, the analysis of the bias factor vs. compound structure relationship showed that compounds with a 6-membered ring always represented higher bias for the β-arrestin pathway than the ones with a 5-membered ring (**8** vs. **7**; **TR-AF-49** vs. **6**; **5** vs. **4**; Table 3, Figure 5). The structure element that could be linked to higher bias among aliphatic derivatives (except **11**) was straight aliphatic moiety over branched (**12**, **14** vs. **10**; **17**, **18** vs. **16**). The most active H_4_R ligand in the whole series (**6**), presented balance activity towards both cAMP and β-arrestin pathways.

#### 2.3.5. Toxicity Evaluation of Compound **6**

Toxic substances can affect the cells. Early in the research process, it is crucial to determine whether obtained compounds can induce such effects. From our series of compounds, compound **6** was selected for toxicity evaluation. MTS assay was used to test toxicity on HepG2 and SH-SY5Y cell lines. HepG2 cells closely reflect the human liver cell model [66] whereas SH-SY5Y neuroblastoma cells are used in models of neurodegenerative diseases (especially Parkinson’s disease) to study the cellular and molecular factors that lead to these disorders [67].

**Figure 6:**
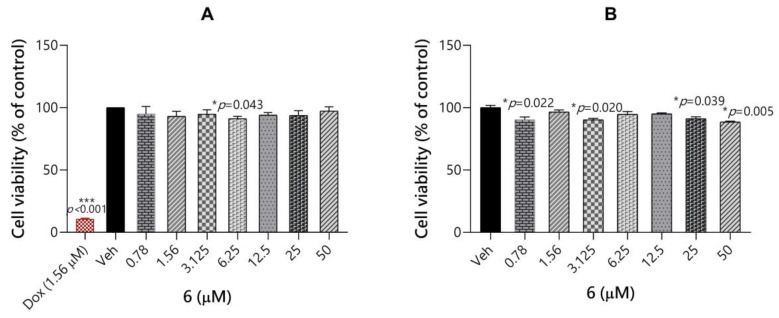
Toxicity evaluation of compound **6** and cytostatic drug doxorubicin (Dox) in HepG2 (**A**) and SH-SY5Y cells (**B**). Each point represents the mean ± SEM of two independent experiments, each of which consisted of three replicates per treatment group. Statistical analyses were performed using GraphPad Prism software 8.0. Statistical significance was evaluated by one-way ANOVA with post-hoc Dunnett test at significance level α = 0.05; *p*-values are detailed in the figure.

Compound **6**, at concentrations ranging from 0.78 µM to 50 µM, was incubated with the respective cell lines for 48 h (HepG2) or 24 h (SH-SY5Y). Then, MTS reagent was added and absorbances at 490 nm were read after 1 h. The recorded results are shown in Figure 6A,B. For both cell lines, compound **6** did not reduce their viability by more than 50%, even at the highest concentration. The reduction in SH-SY5Y cell viability (Figure 6B) of an average of 4–12% was observed over the range of concentrations. For the HepG2 cell line (Figure 6A), the compound did not show significant toxicity in the tested concentration range (except for 6.25 µM concentration).

#### 2.3.6. Permeability of Compound **6** through Blood Brain Barrier

The ability of compound **6** to cross the blood–brain barrier (BBB) was checked using the experimental PAMPA method as described previously [68]. This test is a popular method for estimating the possibility of crossing BBB through passive transport. The compound **6** was tested at a concentration of 200 µM and the incubation time was 5 h. Caffeine was used as a standard well-permeable compound. The results obtained are shown in Table 4. Compound **6** showed a high permeability. The calculated P_e_ value for this compound (P*_e_* = 12.26 × 10^−6^ cm/s) was even slightly higher than for caffeine (P*_e_* = 9.78 × 10^−6^ cm/s). In addition, mass retention (R%) was calculated, which was 3.18% for **6** and 1.54% for caffeine. These values show that both compound **6** and caffeine were retained, to a small extent, in the artificial membrane.

### 2.4. In Vivo Pharmacological Studies

The localization of H_4_Rs in various immune and neuronal cells [4] suggests their involvement in the mechanisms of pain transduction, transmission, and perception. It has been proven that H_4_R ligands show analgesic properties in pain, especially of inflammatory origin [27,31]. Pain and pruritus are distinct unpleasant sensations, but, in many ways, they are closely related. Both sensations share many integral similarities such as largely overlapping mediators and receptors [69,70]. It has been reported that H_4_R antagonists effectively attenuate experimental pruritus [71]. The above-mentioned data encouraged us to test compound **6** in animal models of inflammatory pain and itch.

#### 2.4.1. Antinociceptive Activity of Compound **6** in Formalin Test

A commonly used screening method to test new molecules with analgesic potential is the formalin test. Local injection of formalin induces two phases of the nociceptive response. The early phase (I) is associated with immediate activation of the nociceptors mainly dependent on chemical stimulation of TRPA1 receptors and is related rather to acute neurogenic pain. The late phase (II) is the result of tissue damage, a subsequent inflammatory response, and sensitisation of the spinal reflex circuits. Furthermore, it has been suggested that formalin induces pathological changes that resemble those observed in nerve injury and neuropathic pain [72]. The biphasic response and a plethora of mechanisms involved in the nociceptive response to formalin make the formalin test a valuable tool in the assessment of the analgesic efficacy of a variety of compounds.

**Figure 7:**
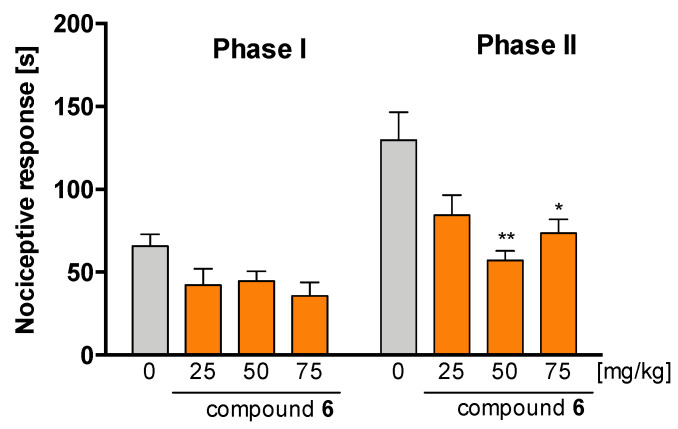
The effect of compound **6** on the duration of licking/biting behaviour in the acute phase (0–5 min after formalin injection and in the late phase (15–30 min after formalin injection). The tested compound or vehicle (1% Tween 80) were administered 30 min intraperitoneally (i.p.) before the test. The results are presented as bar plots showing the mean ± SEM. Statistical analysis: one-way ANOVA followed by Dunnett’s post hoc test, * *p* < 0.05, ** *p* < 0.01, *n* = 8–10 mice per group.

The administration of compound **6** to mice did not significantly affect the duration of the nociceptive response in the acute phase of the formalin test [F(3,30) = 1.547, *p* = 0.22] (Figure 7), but at the doses of 50 mg/kg and 75 mg/kg it significantly attenuated the paw licking or biting behaviour in the late phase [F(3,30) = 2.955, *p* < 0.01]. The results show that compound **6** has no significant influence on acute pain but effectively attenuates inflammatory pain. Interestingly, the analgesic effect had no dose-dependent character. The most potent effect was observed at a dose of 50 mg/kg (44.02% of the control group). Administration of a higher dose of 75 mg/kg resulted in a less pronounced effect (56.62% of the control group). This u-shaped response is often observed in analgesic agents, and in this case, it may be the result of the fact that H_4_R plays a different role in inflammatory cells and neurones. The blockade of H_4_Rs expressed in inflammatory cells results in anti-inflammatory and analgesic effects. On the contrary, the activation of neuronal H_4_Rs leads to analgesia [73]. We hypothesize that the higher dose of compound **6** could antagonize neuronal H_4_Rs in a more pronounced way than the lower dose, thus attenuating the overall analgesic effect.

#### 2.4.2. Antinociceptive and Anti-Inflammatory Activity of Compound **6** in Carrageenan-Induced Inflammatory Pain and Oedema

**Figure 8:**
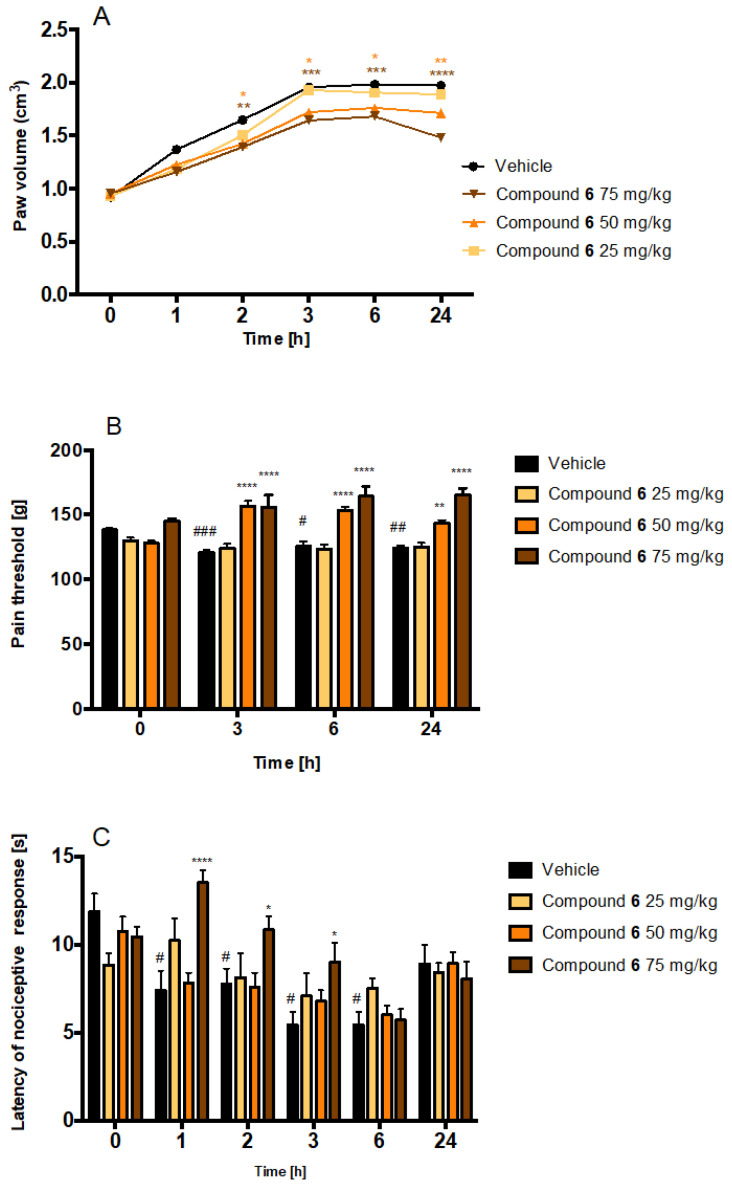
Effect of compound **6** on carrageenan-induced oedema (**A**), mechanical hyperalgesia (**B**) and thermal hyperalgesia (**C**) developed after subplantar injection of 1% carrageenan in rats. Data are expressed as means ± SEM for 5–6 animals. Time 0—the initial reaction considered as the nociceptive reaction before carrageenan administration. Statistical significance compared to vehicle-treated animals: * *p* < 0.05, ** *p* < 0.01, *** *p* < 0.001, **** *p* < 0.0001. Statistical analysis: two-way ANOVA post hoc Bonferroni test. Statistical significance compared to initial value (0 time point) in the vehicle-treated group: # *p* < 0.05, ## *p* < 0.01, ### *p* < 0.0001. Statistical analysis: one-way ANOVA followed by Dunnett’s post hoc test.

Compound **6** showed activity in the late phase of the formalin test, which revealed its analgesic activity in inflammatory pain. We wanted to confirm the activity in an additional model of inflammation in another species. To evaluate the influence of compound **6** on acute inflammation, such as oedema and hyperalgesia, we tested it in the carrageenan-induced inflammation model in rats. Subplantar injection of carrageenan significantly induced oedema (F(5,100) = 31.0, *p* < 0.0001). The paw volume increased from 0.92 ± 0.03 cm^3^ before carrageenan injection to the values of 1.36 ± 0.03 cm^3^, 1.64 ± 0.03 cm^3^, 1.95 ± 0.04 cm^3^, 1.98 ± 0.07 cm^3^ and 1.98 ± 0.07 cm^3^, respectively 1, 2, 3, 6, and 24 h after injection, which corresponds to the increase by 48.9%, 79.3%, 113.0%, 115.2% and 114.1%, respectively. The time course of the development of rat paw oedema (Figure 8A) shows that compound **6** significantly reduced paw oedema (F3,20) = 11.33, *p* = 0.0001). The effect was dose-dependent, and the administration of the most potent dose of 75 mg/kg also resulted in the paw volume increase but only by 22.1%, 46.3%, 72.6%, 76.8% and 55.8%. As oedema is one of the most significant symptoms of inflammation resulting from the release of inflammatory mediators, we may conclude that compound **6** has some anti-inflammatory activity. Experiments with analgesimeter and Plantar test apparatus showed that carrageenan induces significant mechanical and thermal hyperalgesia (F(3, 15) = 40.48, *p* < 0.001 and F(5, 25) = 9.85, *p* < 0.001). The response to mechanical stimuli was observed as the pain withdrawal threshold (Figure 8B) decreased from the value of 138.33 ± 1.05 g (baseline) before carrageenan injection to the value of 120.83 ± 2.0 g (87.3% of the baseline) 3 h after injection, 125.83 ± 2.51 (90.9% of the baseline) 6 h after injection, and 124.17 ± 2.0 (89.7% of the baseline). Compound **6** at the dose of 50 mg/kg significantly increased the pain withdrawal threshold to 122.1%, 119.5% and 111.7% of the baseline. Whereas at the dose of 75 mg/kg compound **6** significantly increased the pain withdrawal threshold to 107.5%, 113.2% and 113.8% of the baseline. The results obtained confirmed its analgesic activity in mechanical inflammatory hyperalgesia. The interesting fact is that the effect had a long-lasting character and was observed even after 24 h after compound administration. We claim that the effect may result from the inhibition of the release of inflammatory mediators and subsequent inhibition of peripheral and central sensitization. In the vehicle-treated group, the response for thermal stimuli observed as the paw withdrawal latency (Figure 8C) decreased from the value of 11.90 ± 0.98 s (baseline) before carrageenan injection to the values of 7.43 ± 1.08 s (62.44% of the baseline), 7.75 ± 0.89 s (65.13% of the baseline), 5.43 ± 0.76 s (45.63 % of the baseline), 5.45 ± 0.70 s (45.80% of the baseline) and 8.90 ± 1.06 s (74.79% of the baseline) 1, 2, 3, 6 and 24 h after the injection, respectively. Compound **6** only at a dose of 75 mg/kg significantly (F(3,20) = 25.22, *p* < 0.0001) increased paw withdrawal latency. The effect was not as persistent as in the mechanical hyperalgesia and was observed only 1, 2 and 3 h after induction of inflammation. The administration of 75 mg/kg increased the latency of paw withdrawal to 129.5%, 103.6% and 86.3% of baseline 1, 2 and 3 after inflammation induction, respectively. The results (the level of pain reactivity over-reaching baseline) show that compound **6** attenuates inflammatory hypersensitivity and induces analgesia.

Taking into account all the results of the analgesic activity of compound **6**, we propose the hypothesis that its anti-inflammatory properties resulting from the antagonism of H_4_R are central to the overall in vivo analgesic profile of the compound. As an H_4_R antagonist, compound **6** can reduce inflammation by inhibiting the release of inflammatory mediators from immune cells decreasing the migration of immune cells to the site of inflammation. It may subsequently inhibit the process of inflammatory sensitization of the peripheral nerve endings and synapses in the dorsal horn of the spinal cord. The results of the formalin test support the hypothesis. On the one hand, compound **6** did not affect the first phase, which resulted from the direct stimulation of nociceptors, proving that this compound had no impact on processes such as transformation and transduction. On the other hand, compound **6** significantly inhibited the late phase, which depends at least partly on inflammatory sensitization. Significant anti-inflammatory activity was additionally confirmed in the carrageenan-induced inflammatory model, where compound **6** reduced oedema formation and inflammatory hyperalgesia.

#### 2.4.3. Antipruritic Effect of Compound **6** in Histamine- and Chloroquine-Induced Pruritus

**Figure 9:**
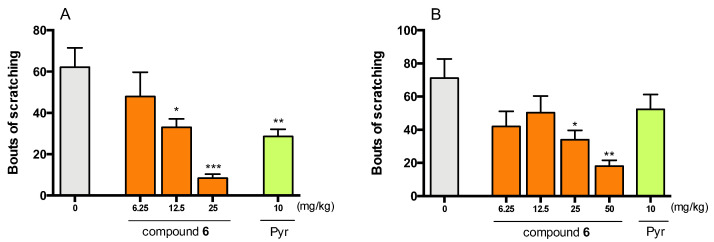
The effect of compound **6** and reference compound pyrilamine (Pyr) on the number of scratching bouts during 60 min-long observation in histamine-induced pruritus (**A**) and 30 min-long observation in chloroquine-induced pruritus (**B**). The tested compound or vehicle (1% solution of Tween 80) was administered intraperitoneally (i.p.) 30 min before the intradermal (i.d.) injection of histamine or chloroquine. The results are presented as bar plots showing the mean ± SEM. Statistical analysis: one-way ANOVA followed by Dunnett’s post hoc test, * *p* < 0.05, ** *p* < 0.01, *** *p* < 0.001, *n* = 8–10 mice per group.

Chemically induced itch can be classified into histamine-dependent and histamine-independent subclasses. The first type results from the stimulation of histamine H_1_ receptors (H_1_Rs) on itch-mediating primary sensory neurons. The second one results from the stimulation of distinct types of ion channels and receptors such as Mas-related G protein-coupled receptors (Mrgprs), protease-activated receptors (PARs), bile acid receptors (TGR5), toll-like receptors (TLRs), and transient receptor potential subfamily V1/A1 (TRPV1/A1). An example of histamine-independent itch is the sensation induced by the MrgprA3 agonist–chloroquine (CQ) [74]. Classical antihistamine agents, which are H_1_Rs antagonists, attenuate histamine-dependent itch, but not the histamine-independent [75]. We tested compound **6** in two different pruritus mice models to assess its potential to affect histamine-dependent and histamine-independent itch (Figure 9).

Intradermal injection of histamine induced scratching bouts in the amount of 62.11 ± 9.4. Compound **6** significantly reduced scratching behaviour (F(3,30) = 7.80, *p* < 0.001). Administration of the most effective dose of 25 mg/kg resulted in a decrease in scratch bouts to the value of 8.37 ± 1.96 (13.47% of the control value). Histamine H_1_R antagonist pyrilamine (used as reference ligand) at the dose of 10 mg/kg significantly reduced scratching behaviour to the value of 28.56 ± 3.55 scratch bouts.

Intradermal injection of the CQ solution resulted in robust scratching behaviour manifested as 72.20 ± 11.59 scratch bouts during the 30 min observation. A single administration of compound **6** at doses 25 mg/kg and 50 mg/kg significantly decreased the number of scratch bouts but the effects of doses of 6.25 mg/kg and 12.5 mg/kg were not statistically significant (F(5,44) = 19.14, *p* < 0.0001). The most effective dose of compound **6** was 50 mg/kg, which decreased the number of scratch bouts to 18.14 ± 3.48 (25.12% of the control value) whereas pyrilamine (at the dose of 10 mg/kg) did not significantly reduce scratching behaviour. When comparing the activity of the tested in both used models, it should be noted that the efficacy (higher maximal effect) and potency (lower doses needed to obtain the same result) of the compound were better in histamine-induced pruritus. Nevertheless, compound **6** was also active in histamine-independent itch, which contrasts with the activity of pyrilamine representing a commonly used drug in the treatment of pruritus. This H_1_R antagonist showed activity in histamine-induced itch but was inactive in CQ-induced itch. The wide spectrum of antipruritic activity of compound **6** is very promising, considering that histamine-independent itch is still a crucial clinical problem in pruritus treatment.

## 3. Materials and Methods

### 3.1. Synthesis of Compounds

Reagents were purchased from Alfa Aesar (Haverhill, MA, USA) or Sigma Aldrich (Darmstadt, Germany) and were used without further purification. Melting points (Mp.) were measured on a MEL-TEMP II (LD Inc., Long Beach, CA, USA) melting point apparatus and are uncorrected. Mass spectra (LC/MS) were conducted on Waters TQ Detector (Water Corporation, Milford, CT, USA) mass spectrometer. Retention times (t_R_) are given in minutes. All compounds showed UPLC/MS purity > 96%. ^1^H NMR spectra were recorded on a Mercury 300 MHz PFG spectrometer (Varian, Palo Alto, CA, USA) in DMSO-d_6_. ^13^C NMR spectra were recorded on FTNMR 500 MHz spectrometer (Joel Ltd., Akishima, Tokyo, Japan) in DMSO-d_6_. Chemical shifts were expressed in parts per million (ppm) using the solvent signal as an internal standard. Data are reported in the following order: multiplicity (br., broad; d, doublet; m, multiplet; quin, quintet; s, singlet; sxt, sextet; t, triplet), approximate coupling constants *J* expressed in Hertz (Hz), number of protons. Elemental analysis was performed on an Elemental Analyser Vario El-III (Hanau, Germany). The results are in agreement with the theoretical values within ± 0.4%. TLC data were obtained with Merck (Darmstadt, Germany) silica gel 60F_254_ aluminium sheets with the following detection with UV light and evaluation with Dragendorff’s reagent (solvent system: methylene chloride:methanol 1:1).

#### 3.1.1. Synthesis of Esters

Esters 1a–1k, 1m–1r and 1s were obtained from proper commercially available carboxylic acid according to the method described previously [61].

#### 3.1.2. Synthesis of Triazines **2**–**20**—General Procedure

To a freshly prepared sodium methoxide (12 mmol Na in 5 mL of methanol) 4-methylpiperazin-1-yl biguanide dihydrochloride (5 mmol) was added and the mixture was stirred at room temperature for 2–3 h. Then, a crude suitable carboxylic acid ester (5 mmol) was added and the mixture was further stirred for 48–84 h at room temperature. After that time, the solvent was evaporated, and was added to the residue water (5 mL). The precipitated product was filtrated and crystallized from a proper solvent.

*4-Cyclopropyl-6-(4-methylpiperazin-1-yl)-1,3,5-triazin-2-amine* (**2**)

Synthesis from prepared methyl cyclopropanecarboxylate **1a** (5 mmol). Crystallization: CH_3_OH. Yield 3%, m.p. 156–159 °C, C_11_H_18_N_6_ (MW = 234.31). LC/MS^+^: purity: 100%, t_R_ = 0.649, (ESI) *m*/*z* [M+H]^+^ 235.097. ^1^H NMR (300 MHz, DMSO-d_6_) δ: 6.60 (br. S., 2H), 3.63 (br. s., 4H), 2.25 (t, *J* = 4.62 Hz, 4H), 2.16 (s, 3H), 1.56–1.75 (m, 1H), 0.70–0.95 (m, 4H). ^13^C NMR (126 MHz, DMSO-d_6_) δ: 178.7, 167.0, 164.7, 54.9, 46.3, 42.8, 17.7, 9.2. Anal. Calcd. For C_11_H_18_N_6_: C, 56.43; H, 7.68; N, 35.87%. Found: C, 56.06; H, 7.56; N, 35.61%.

*4-Cyclobutyl-6-(4-methylpiperazin-1-yl)-1,3,5-triazin-2-amine* (**3**)

Synthesis from prepared methyl cyclobutanecarboxylate **1b** (5 mmol). Crystallization: CH_3_OH. Yield 14%, m.p. 127–132 °C, C_12_H_20_N_6_ (MW = 248.12). LC/MS^+^: purity: 100%, t_R_ = 0.671, (ESI) *m*/*z* [M+H]^+^ 249.110. ^1^H NMR (300 MHz, DMSO-d_6_) δ: 6.69 (br. s., 2H), 3.67 (br. s., 4H), 3.20 (quin, *J* = 8.46 Hz, 1H), 2.02–2.36 (m, 11H), 1.84–2.00 (m, 1H), 1.68–1.83 (m, 1H). ^13^C NMR (126 MHz, DMSO-d_6_) δ: 179.6, 167.4, 165.1, 54.9, 46.3, 42.9, 42.1, 26.5, 18.3. Anal. Calcd. For C_12_H_20_N_6_: C, 56.65; H, 7.79; N, 32.67%. Found: C, 57.20; H, 7.94; N, 32.62%.

*4-Cyclopentyl-6-(4-methylpiperazin-1-yl)-1,3,5-triazin-2-amine* (**4**)

Synthesis from prepared methyl cyclopentanecarboxylate **1c** (5 mmol). Crystallization: CH_3_OH. Yield 9%, m.p. 163–169 °C, C_13_H_22_N_6_ (MW = 262.37). LC/MS^+^: purity: 100%, t_R_ = 0.722, (ESI) *m*/*z* [M+H]^+^ 263.268.^1^H NMR (300 MHz, DMSO-d_6_) δ: 6.65 (br. s., 2H), 3.66 (br. s., 4H), 2.74 (quin, *J* = 8.02 Hz, 1H), 2.26 (t, *J* = 4.74 Hz, 4H), 2.16 (s, 3H), 1.50–1.85 (m, 8H). ^13^C NMR (126 MHz, DMSO-d_6_) δ: 181.2, 167.4, 165.1, 54.9, 47.9, 46.3, 42.8, 31.9, 26.1. Anal. Calcd. For C_13_H_22_N_6_: C, 59.51; H, 8.45; N, 32.04%. Found: C, 59.66; H, 8.46; N, 32.01%.

*4-Cyclohexyl-6-(4-methylpiperazin-1-yl)-1,3,5-triazin-2-amine* (**5**)

Synthesis from prepared methyl cyclohexanecarboxylate **1d** (5 mmol). Crystallization: CH_3_OH. Yield 14%, m.p. 133–138 °C, C_14_H_24_N_6_ (MW = 276.39). LC/MS^+^: purity: 56.50%, t_R1_ = 2.674, (ESI) *m*/*z* [M+H]^+^ 277.255 + 43.50%, t_R2_ = 2.992, (ESI) *m*/*z* [M+H]^+^ 277.179. ^1^H NMR (300 MHz, DMSO-d_6_) δ: 6.65 (br. s., 2H), 3.66 (br. s., 4H), 2.07–2.37 (m, 8H), 1.55–1.89 (m, 5H), 1.34–1.53 (m, 2H), 1.04–1.33 (m, 3H). ^13^C NMR (126 MHz, DMSO-d_6_) δ: 181.1, 167.4, 165.1, 54.9, 46.7, 46.3, 42.8, 31.1, 26.2, 26.1. Anal. Calcd. For C_14_H_24_N_6_: C, 60.84; H, 8.75; N, 30.41%. Found: C, 60.69; H, 8.75; N, 30.36%.

*4-(Cyclopentylmethyl)-6-(4-methylpiperazin-1-yl)-1,3,5-triazin-2-amine* (**6**)

Synthesis from prepared methyl 2-cyclopentylacetate **1e** (5 mmol). Crystallization: C_2_H_5_OH/H_2_O. Yield 22%, m.p. 165–168 °C, C_14_H_24_N_6_ (MW = 276.39). LC/MS^+^: purity: 100%, t_R_ = 2.34, (ESI) *m*/*z* [M+H]^+^ 277.44. ^1^H NMR (300 MHz, DMSO-d_6_) δ: 6.65 (br. s., 2H), 3.65 (br. s., 4H), 2.21–2.37 (m, 7H), 2.16 (s, 3H), 1.37–1.77 (m, 6H), 1.05–1.30 (m, 2H). ^13^C NMR (126 MHz, DMSO-d_6_) δ: 177.5, 167.3, 165.0, 54.9, 46.3, 44.7, 42.9, 38.5, 32.5, 25.1.

*4-(Cyclopent-2-enylmethyl)-6-(4-methylpiperazin-1-yl)-1,3,5-triazin-2-amine* (**7**)

Synthesis from prepared methyl 2-(cyclopen-1-en-1-yl)acetate **1f** (5 mmol). Crystallization: C_2_H_5_OH/H_2_O. Yield 20%, m.p. 166–169 °C, C_14_H_22_N_6_ (MW = 274.36). LC/MS^+^: purity: 100%, t_R_ = 2.01, (ESI) *m*/*z* [M+H]^+^ 275.45. ^1^H NMR (300 MHz, DMSO-d_6_) δ: 6.67 (br. s., 2H), 5.58–5.81 (m, 2H), 3.66 (br. s., 4H), 3.08 (br. s., 1H), 2.37–2.44 (m, 1H), 2.12–2.36 (m, 10H), 1.87–2.03 (m, 1H), 1.36–1.53 (m, 1H). ^13^C NMR (126 MHz, DMSO-d_6_) δ: 177.0, 167.3, 165.0, 135.5, 130.8, 54.9, 46.3, 44.7, 44.1, 42.9, 31.9, 29.7.

*4-(Cyclohexenylmethyl)-6-(4-methylpiperazin-1-yl)-1,3,5-triazin-2-amine* (**8**)

Synthesis from prepared methyl 2-(cyclohex-1-en-1-yl)acetate **1g** (5 mmol). Crystallization: C_2_H_5_OH/H_2_O. Yield 22%, m.p. 149–153 °C, C_15_H_24_N_6_ (MW = 288.41). LC/MS^+^: purity: 100%, t_R_ = 2.50, (ESI) *m*/*z* [M+H]^+^ 289.41. ^1^H NMR (500 MHz, DMSO-d_6_) δ: 6.46–6.94 (m, 2H), 5.37 (br. s., 1H), 3.63 (br. s., 4H), 2.94 (s, 2H), 2.24 (t, *J* = 4.73 Hz, 4H), 2.14 (s, 3H), 1.91 (br. s., 4H), 1.37–1.59 (m, 4H). ^13^C NMR (126 MHz, DMSO-d_6_) δ: 176.3, 167.4, 165.1, 134.8, 123.5, 54.9, 47.7, 46.3, 42.9, 28.6, 25.3, 22.9, 22.4.

*4-(1-Adamantylmethyl)-6-(4-methylpiperazin-1-yl)-1,3,5-triazin-2-amine* (**9**)

Synthesis from prepared methyl 2-(adamantan-1-yl)acetate **1h** (5 mmol). Purification: product insoluble in reflux CH_3_CN. Yield 4%, m.p. 200–201 °C, C_19_H_30_N_6_ (MW = 342.48). LC/MS^+^: purity: 100%, t_R_ = 3.46, (ESI) *m*/*z* [M+H]^+^ 343.32. ^1^H NMR (300 MHz, DMSO-d_6_) δ: 6.63 (br. s., 2H), 3.65 (br. s., 4H), 2.03–2.32 (m, 9H), 1.88 (br. s., 3H), 1.42–1.69 (m, 12H). ^13^C NMR (126 MHz, DMSO-d_6_) δ: 175.4, 167.1, 164.8, 54.9, 53.2, 46.3, 43.0, 37.0, 33.7, 28.7.

*4-Butyl-6-(4-methylpiperazin-1-yl)- 1,3,5-triazin-2-amine* (**10**)

Synthesis from commercial methyl pentanoate **1i** (5 mmol). Crystallization: CH_3_OH/H_2_O. Yield 31%., m.p. 117–118 °C, C_12_H_22_N_6_ (MW = 250.34). LC/MS^+/−^: purity: 97.39%, t_R_ = 1.64 (ESI) *m*/*z* [M+H]^+^ 251.21. ^1^H NMR (300 MHz, DMSO-d_6_) δ: 6.66 (br. s., 2H), 3.66 (br. s., 4H), 2.20–2.41 (m, 6H), 2.16 (s, 3H), 1.57 (quin, *J* = 1.00 Hz, 2H), 1.28 (sxt, *J* = 1.00 Hz, 2H), 0.86 (t, *J* = 7.33 Hz, 3H). ^13^C NMR (DMSO-d_6_, 126 MHz) δ: 178.0, 167.3, 165.0, 54.9, 46.3, 42.8, 38.4, 29.7, 22.5, 14.4. Anal. Calcd. For C_12_H_22_N_6_: C, 57.59; H, 8.86; N, 33.56%. Found: C, 57.51; H, 9.31; N, 34.15%.

*4-(4-Methylpiperazin-1-yl)-6-(pentan-2-yl)-1,3,5-triazin-2-amine* (**11**)

Synthesis from prepared methyl 2-methylpentanoate **1j** (5 mmol). Crystallization: CH_3_OH/H_2_O. Yield 8%, m.p. 138–140 °C, C_13_H_24_N_6_ (MW = 264.37). LC/MS^+^: purity: 100%, t_R_ = 2.05, (ESI) *m*/*z* [M+H]^+^ 265.30. ^1^H NMR (300 MHz, DMSO-d_6_) δ: 6.68 (br. s., 2H), 3.66 (br. s., 4H), 2.34–2.44 (m, 1H), 2.20–2.33 (m, 4H), 2.16 (s, 3H), 1.53–1.74 (m, 1H), 1.00–1.43 (m, 6H), 0.75–0.89 (m, 3H). ^13^C NMR (126 MHz, DMSO-d_6_) δ: 181.7, 167.5, 165.1, 54.9, 46.3, 42.9, 42.1, 37.7, 20.7, 19.6, 14.6.

*4-(2-Methylbutyl)-6-(4-methylpiperazin-1-yl)-1,3,5-triazin-2-amine* (**12**)

Synthesis from prepared methyl 3-methylpentanoate **1k** (5 mmol). Crystallization: CH_3_OH/H_2_O. Yield 8%, m.p. 115–116 °C, C_13_H_24_N_6_ (MW = 264.37). LC/MS^+^: purity: 100%, t_R_ = 1.99, (ESI) *m*/*z* [M+H]^+^ 265.30. ^1^H NMR (300 MHz, DMSO-d_6_) δ: 6.69 (br. s., 2H), 3.65 (br. s., 4H), 2.21–2.40 (m, 5H), 2.03–2.20 (m, 4H), 1.79–1.99 (m, 1H), 1.02–1.41 (m, 2H), 0.73–0.91 (m, 6H). ^13^C NMR (126 MHz, DMSO-d_6_) δ: 177.4, 167.3, 165.0, 54.9, 46.3, 45.8, 42.9, 33.5, 29.5, 19.7, 11.8.

*4-(2-Methoxypropyl)-6-(4-methylpiperazin-1-yl)-1,3,5-triazin-2-amine* (**13**)

Synthesis from commercial ethyl crotonate **1l** (5 mmol). Crystallization: CH_3_CN. Yield 10%, m.p. 148–151 °C, C_12_H_22_N_6_O (MW = 266.34). LC/MS^+^: purity: 55.28%, t_R_ = 0.98, (ESI) *m*/*z* [M+H]^+^ 267.23, purity: 44.72%, t_R_ = 1.15, (ESI) *m*/*z* [M+H]^+^ 267.23. ^1^H NMR (300 MHz, DMSO-d_6_) δ: 6.72 (br. s., 2H), 3.73–3.88 (m, 1H), 3.66 (br. s., 4H), 3.18 (s, 3H), 2.56-2.68 (m, 1H), 2.20–2.38 (m, 5H), 2.16 (s, 3H), 1.07 (d, *J* = 6.45 Hz, 3H).

*4-Isopentyl-6-(4-methylpiperazin-1-yl)-1,3,5-triazin-2-amine* (**14**)

Synthesis from prepared methyl 4-methylpentanoate **1m** (5 mmol). Crystallization: CH_3_OH/H_2_O. Yield 16%, m.p. 123–126 °C, C_13_H_24_N_6_ (MW = 264.37). LC/MS^+^: purity: 100%, t_R_ = 2.16, (ESI) *m*/*z* [M+H]^+^ 265.48. ^1^H NMR (300 MHz, DMSO-d_6_) δ: 6.66 (br. s., 2H), 3.65 (br. s., 4H), 2.22–2.36 (m, 6H), 2.16 (s, 3H), 1.37–1.62 (m, 3H), 0.86 (d, *J* = 5.90 Hz, 6H). ^13^C NMR (126 MHz, DMSO-d_6_) δ: 178.3, 167.3, 165.0, 54.9, 46.3, 42.9, 36.8, 36.7, 27.9, 22.9.

(*E*) *4-(But-1-enyl)-6-(4-methylpiperazin-1-yl)-1,3,5-triazin-2-amine* (**15**)

Synthesis from prepared methyl pent-2-enoate **1n** (5 mmol). Crystallization: CH_3_OH/H_2_O. Yield 13%, m.p. 125–126 °C, C_12_H_20_N_6_ (MW = 248.33). LC/MS^±^: purity: 96.43%, t_R_ = 1.56, (ESI) *m*/*z* [M+H]^+^ 249.22; purity: 3,57%, t_R_ = 1.72, (ESI) *m*/*z* [M+H]^+^ 249.15. ^1^H NMR (300 MHz, DMSO-d_6_) δ: 6.69 (br. s., 2H), 5.34–5.70 (m, 2H), 3.65 (br. s., 4H), 3.05 (d, *J* = 6.67 Hz, 2H), 2.22–2.31 (m, 4H), 2.17 (s, 3H), 1.60 (d, *J* = 0.77 Hz, 3H).

*4-(But-3-enyl)-6-(4-methylpiperazin-1-yl)-1,3,5-triazin-2-amine* (**16**)

Synthesis from prepared methyl pent-4-enoate **1o** (5 mmol). Crystallization: CH_3_CN. Yield 8%, m.p. 132–133 °C, C_12_H_20_N_6_ (MW = 248.33). LC/MS^+^: purity: 100%, t_R_ = 1.40, (ESI) *m*/*z* [M+H]^+^ 249.2. ^1^H NMR (300 MHz, DMSO-d_6_) δ: 6.71 (br. s., 2H), 5.74–5.92 (m, 1H), 4.84–5.13 (m, 2H), 3.66 (br. s., 4H), 2.30–2.44 (m, 4H), 2.26 (t, *J* = 4.69 Hz, 4H), 2.16 (s, 3H). ^13^C NMR (126 MHz, DMSO-d_6_) δ: 177.2, 167.3, 165.0, 138.6, 115.4, 54.9, 46.3, 42.9, 37.8, 31.3.

*4-(4-Methylpiperazin-1-yl)-6-(pent-4-en-2yl)-1,3,5-triazin-2-amine* (**17**)

Synthesis from prepared methyl 2-methylpent-4-enoate **1p** (5 mmol). Crystallization: CH_3_OH/H_2_O. Yield 10%, m.p. 134–136 °C, C_13_H_22_N_6_ (MW = 262.35). LC/MS^±^: purity: 100%, t_R_ = 1,79, (ESI) *m*/*z* [M+H]^+^ 263.22. ^1^H NMR (300 MHz, DMSO-d_6_) δ: 6.68 (br. s., 2H), 5.60–5.81 (m, 1H), 4.85–5.05 (m, 2H), 3.66 (br. s., 4H), 2.51–2.56 (m, 1H), 2.35–2.45 (m, 1H), 2.27 (t, *J* = 4.87 Hz, 4H), 2.03–2.20 (m, 4H), 1.09 (d, *J* = 6.67 Hz, 3H). ^13^C NMR (126 MHz, DMSO-d_6_) δ: 180.9, 167.4, 165.1, 137.6, 116.5, 54.9, 46.3, 42.9, 42.0, 19.1.

*4-(2-Methylbut-3-enyl)-6-(4-methylpiperazin-1-yl)-1,3,5-triazin-2-amine* (**18**)

Synthesis from prepared methyl 3-methylpent-4-enoate **1r** (5 mmol). Crystallization: CH_3_OH/H_2_O. Yield 25%, m.p. 113–115 °C, C_13_H_22_N_6_ (MW = 262.35). LC/MS^+^: purity: 100%, t_R_ = 1.78, (ESI) *m*/*z* [M+H]^+^ 263.24. ^1^H NMR (300 MHz, DMSO-d_6_) δ: 6.69 (br. s., 2H), 5.66–5.94 (m, 1H), 4.81–5.03 (m, 2H), 3.66 (br. s., 4H), 2.63–2.80 (m, 1H), 2.34–2.44 (m, 1H), 2.20–2.32 (m, 5H), 2.16 (s, 3H), 0.94 (d, *J* = 6.45 Hz, 3H). ^13^C NMR (126 MHz, DMSO-d_6_) δ: 176.6, 167.3, 165.0, 144.2, 113.2, 54.9, 46.3, 45.4, 42.9, 35.8, 19.8.

*4-(4-Methylpiperazin-1-yl)-6-pentyl-1,3,5-triazin-2-amine* (**19**)

Synthesis from commercial ethyl hexanoate **1q** (5 mmol). Crystallization: CH_3_OH/H_2_O. Yield 40%, m.p. 173–178 °C, C_13_H_24_N_6_ (MW = 264.37). LC/MS^+/−^: purity: 100%, t_R_= 2.19, (ESI) *m*/*z* [M+H]^+^ 265.23. ^1^H NMR (300 MHz, DMSO-d_6_) δ: 6.66 (br. s., 2H), 3.66 (br. s., 4H), 2.20–2.38 (m, 6H), 2.16 (s, 3H), 1.60 (quin, *J* = 7.33 Hz, 2H), 1.18–1.30 (m, 4H), 0.84 (t, *J* = 6.74 Hz, 3H). ^13^C NMR (126 MHz, DMSO-d_6_) δ: 178.0, 167.3, 165.0, 54.9, 46.3, 42.9, 38.6, 31.6, 27.2, 22.5, 14.4. Anal. calcd. for C_13_H_24_N_6_ x H_2_O (MW = 282.39): C, 55.29; H, 9.28; N, 29.76%. Found: C, 55.30; H, 9.71; N, 30.21%.

*(E)-4-(4-Methylpiperazin-1-yl)-6-(pent-2-enyl)-1,3,5-triazin-2-amine* (**20**)

Synthesis from prepared methyl (*E*) hex-3-enoate **1s** (5 mmol). Crystallization: CH_3_OH/H_2_O. Yield 15%, m.p. 117–119 °C, C_13_H_22_N_6_ (MW = 262.36). LC/MS^+/−^: purity: 98.57%, t_R_ = 2.05, (ESI) *m*/*z* [M+H]^+^ 263.35. ^1^H NMR (300 MHz, DMSO-d_6_) δ: 6.71 (br. s., 2H), 5.40–5.68 (m, 2H), 3.65 (br. s., 4H), 3.05 (d, *J* = 5.90 Hz, 2H), 2.26 (t, *J* = 4.87 Hz, 4H), 2.16 (s, 3H), 1.87–2.06 (m, 2H), 0.91 (t, *J* = 7.44 Hz, 3H). ^13^C NMR (126 MHz, DMSO-d_6_) δ: 176.6, 167.4, 165.0, 134.0, 125.5, 54.9, 46.3, 42.9, 42.4, 25.6, 14.1.

### 3.2. In Vitro Biological Studies

#### 3.2.1. Histamine H_4_ Receptor Affinity

Affinities of all triazine derivatives were determined in a [^3^H] histamine (f.c. 10 nM) displacement assay using membrane preparations from S*f*9 cells expressing human H_4_R and co-expressed with G_αi2_ and G_β1γ2_ subunits as described previously [44]. Nonspecific binding was determined in the presence of unlabelled JNJ7777120 (10 mM).

#### 3.2.2. β-Arrestin Recruitment Assay

All used media and reagents except DMSO and tested compounds were purchased from ThermoFisher Scientific, Waltham, MA, USA. DMSO was from CarlRoth (Karlsruhe, Germany) and histamine was from Sigma Aldrich (Darmstadt, Germany). Tango-H4-bla U2OS cells were cultured in McCoy’s 5A medium supplemented according to the provider’s recommendation (dialyzed FBS 10%, NEAA 0.1 mM, HEPES 25 mM, sodium pyruvate 1 mM, penicillin/streptomycin 100 U/mL, zeocin 200 µg/mL, hygromycin 50 µg/mL, G418 (geneticin) 100 µg/mL) in a CO_2_ incubator (HERAcell240, Heraeus, Hanau, Germany) at 37 °C in a humidified atmosphere containing 5% CO_2_. The day before the experiment cells were collected from the culture flask with trypsin and seeded in 384 well black-wall, clear-bottom plate (PerkinElmer, Waltham, MA, USA) in concentration 10,000 cells/well in FreeStyle medium. After 24 h of incubation, the cells were checked under the microscope and the dilutions of tested compounds were prepared and added to the wells. In agonist mode compounds were added to the wells and plates were placed in the incubator (HERAcell240, Heraeus, Hanau, Germany) for 16 h. In antagonist mode, first, the compounds were added to the wells, plates were incubated for 30 min, then histamine solution (corresponding to EC_80_—500 nM) was added and plates were put in the incubator for 16 h. After this time β-lactamase substrate mixture was prepared and added to wells (8 µL/well). Cells were incubated for 2 h at room temperature in the dark then the signal was read using an EnSpire microplate reader (PerkinElmer, Waltham, MA, USA). Drawing of dose-dependent curves and IC_50_ calculation for compounds were made using GraphPad Prism 6 (GraphPad Software, San Diego, CA, USA).

#### 3.2.3. cAMP Accumulation Assay

Intrinsic activity in G protein-dependent pathway was measured with homogenous TR-FRET immunoassay, using LANCE Ultra cAMP kit and CHO-K1 H_4_R cell line or γ-irradiated recombinant CHO-K1 H_4_R cells (all from PerkinElmer). If frozen CHO-K1 H_4_R cells were used cells had been thawed in a 37 °C water bath and placed in the culture flask with DMEM/F12 medium supplemented with 10 % FBS the day before the experiment. If the CHO-K1 H_4_R cell line was used it had been cultivated in DMEM/F12 10% FBS 400 µg/mL geneticin and passaged three times a week. On the experiment day cells were detached using Tryple, collected, centrifuged and resuspended in stimulation buffer (HBSS, 100 mM RO-201724, 5 mM HEPES, 0.1% BSA, pH 7.4). The antagonist activity was measured in a white, opaque, 384-well microplate (PerkinElmer) with a total volume of 20 µL. Cells (1000 cells/well) were incubated with forskolin (10 µM), histamine (140 nM) and tested antagonists (0.003–100 µM) for 30 min at room temperature. After incubation, 5 µL of europium chelate-labelled cAMP tracer and 5 µl of ULight-labelled anti-cAMP mAb working solutions were added, mixed and incubated for 1 h. TR-FRET signal was read using a microplate reader (PerkinElmer, Waltham, MA, USA).

#### 3.2.4. PAMPA Assay

The Gentest^TM^ PAMPA Plate System, purchased from Corning (Tewksbury, MA, USA), was used for permeability evaluation. Caffeine (a reference) and the compound tested were dissolved in DMSO (10 mM stocks) and after dilution to 200 μM in PBS (pH 7.4), added to donor wells. Then, after incubation for 5 h at room temperature, concentrations of tested compounds in donor and acceptor wells were estimated by the mass spectra (LC/MS) method as described previously [68]. The UPLC/MS Waters ACQUITYTM TQD system with the TQ Detector (Waters, Milford, CT, USA) was used for this detection. The assay was performed in triplicate. The permeability value (P_e_) was calculated using the formula described by Chen et al. [76].

#### 3.2.5. Toxicity Evaluation

The hepatoma cell line HepG2 (ATCC^®^ HB-8065TM) and SH-SY5Y CRL-2266 neuroblastoma cell line were used to evaluate the toxicity of tested compounds. Tests were conducted as described previously [77]. CellTiter 96^®^ Aqueous Non-Radioactive Cell Proliferation Assay was purchased from Promega (Madison, WI, USA). Compounds were tested at 7 concentrations (0.78, 1.56, 3.125, 6.25, 12.5, 25 and 50 µM). Cell viability was determined after incubation with compounds for 24 h (HepG2) or 48 h (SH-SY5Y). Each experiment was performed twice in triplicate.

### 3.3. In Vivo Studies

#### 3.3.1. Animals

The experiments were carried out on adult male Albino Swiss mice (CD-1, 18–25 g) and male Wistar rats (Krf:(WI) WU), 180–250 g). The animals were housed in plastic cages in a room at a constant temperature of 20 ± 2 °C, under a light/dark (12:12) cycle and had free access to a standard pellet diet and water. The experimental groups consisted of 6–12 animals, all the animals were used only once and they were killed by cervical dislocation immediately after the assay. The rats were previously anaesthetized with sodium pentobarbital (60 mg/kg). The minimum number of animals was used needed to obtain definite and normally distributed results with the utilized test. Behavioural measures were scored by trained observers, which were blind to experimental conditions. The treatment of laboratory animals in the present study was in full accordance with the respective Polish regulations. All procedures were conducted according to the guidelines of ICLAS (International Council on Laboratory Animal Science) and approved by the Local Ethics Committee of the Jagiellonian University in Kraków (105/2016 and 666/2022).

#### 3.3.2. Formalin Test

The procedure used was essentially the same as that described previously [77,78]. Briefly, the pain was induced by the intraplantar injection of 20 μL of 2.5% formalin solution into the right hind paw of the mice. The total time (in s) spent on licking the injected paw during periods of 0–5 min (early phase, neurogenic) and 15–30 min (late phase, inflammatory) was measured and was considered as an indicator of nociceptive behaviour. Before formalin injection different groups of mice were treated i.p. with vehicle (10 mL/kg, negative control) and the dose–response of the investigated compound.

#### 3.3.3. Carrageenan-Induced Inflammatory Pain and Oedema

The procedure used was described previously [35]. Briefly, the inflammation and paw oedema was induced by subplantar injection of 0.1 mL of 1% carrageenan (made in PBS) into the right hind paw of the rat. The paw volume was measured by the dislocation of the water column of the plethysmometer (Plethysmometer 7140, Ugo Basile). The hyperalgesic response to mechanical stimuli was measured using Analgesy Meter 37215, Ugo Basile. The intensity of the applied force, in grams, was recorded when the paw was withdrawn (withdrawal threshold). Moreover, the hyperalgesic response to thermal stimuli was determined by using a plantar test apparatus (Commat Ltd., Ankara, Turkey). The latency to the heat stimulus was automatically recorded. Three subsequent applications of the heating stimulus were done, separated by 1- to 2-min intervals, and the mean of these measures was taken.

#### 3.3.4. Histamine- and Chloroquine-Induced Pruritus

The surface (around 2 cm^2^) of the mice’s nape was shaved at least 2 days prior to experiments. On the day of the experiment, mice were individually placed in plastic chambers (15 × 15 × 30 cm) and after the 30 min habituation period, they were given an intradermal (i.d.) injection of histamine dihydrochloride (10 µmol/site) or chloroquine (CQ, 200 µg/site), which were dissolved in physiological saline and administrated in the constant volume of 20 μL. Immediately after the injection, scratching (series of movements considered as a single scratching bout) of the injected site by the hind paw was counted for 60 min or 30 min after histamine and CQ administration, respectively [77,78,79,80]. Before pruritogens injection different groups of mice were treated i.p. with vehicle (10 mL/kg, negative control) or the dose–response of investigated compound.

## 4. Conclusions

**Figure 10:**
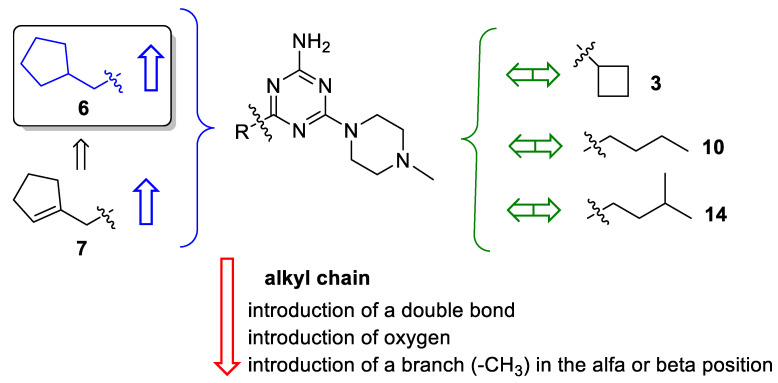
SAR analysis of hH_4_R affinities of alkyl derivatives of 4-(4-methylpiperazin-1-yl)-1,3,5-triazin-2-amines.

In two series (alicyclic and aliphatic), nineteen new alkylaminopiperazinyl-1,3,5-triazines were obtained and pharmacologically evaluated. Most compounds (eleven) showed comparable or weaker affinities for hH_4_R (100 < K_i_ < 600 nM) than the lead structure **TR-AF-49** (K_i_ = 160 nM). Two compounds (**6** and **7**) had good affinities with K_i_ values below 100 nM whereas for six compounds very low affinities were observed (K_i_ > 1000 nM). The summary of SAR analysis is shown in Figure 10. Compounds with substituents such as a cyclobutyl (**3**), a butyl (**10**) or a 3-methylbutyl (**14**) had a comparable affinity to the lead **TR-AF-49** (K_i_ ~ 200 nM). The introduction of a lower ring (a cyclopentyl instead of a cyclohexyl) increased hH_4_R affinity (K_i_ < 100 nM) and compound **6** is the most potent compound in both series. Other changes especially in the alkyl chain led to a decrease in affinity.

All the compounds tested in functional assays (β-arrestin recruitment, cAMP accumulation) in the agonist and antagonist modes showed only antagonistic effects. Although the mode of action was the same (antagonistic), different behaviour was observed toward the tested signalling pathways. Most of the compounds favoured the β-arrestin pathway. Compound **6** showed the highest antagonist activity compared to the other compounds tested and the reference ligand **JNJ7777120**. The analysis of calculated bias coefficients clearly showed the balanced activity of this compound toward the cAMP and β-arrestin pathways. Further in vitro studies of compound **6** demonstrated low neurotoxicity to SH-SY5Y cells and hepatotoxicity to HepG2 cells. The PAMPA assay allowed us to estimate the high capacity of compound **6** to penetrate BBB. In in vivo studies, we found that compound **6**, decreased nociceptive response in some animal models of inflammatory pain also attenuating inflammatory oedema. Furthermore, we showed that the tested compound decreased scratching behaviour in mice in different models of itch. However, compound **6** showed better efficacy and potency in decreasing scratching behaviour than in decreasing the nociceptive response. This observed anti-inflammatory effect is not surprising as H_4_R is located in cells and tissues associated with inflammation. Our previous studies showed that the aryl derivatives of 1,3,5-triazine (e.g., **TR7**) tested in in vivo models of inflammation induced by carrageenan or zymosan decreased the production of inflammatory cytokines such as TNFa and IL-1b and reduced the production of reactive oxygen species [35]. It is possible that compound **6** shows a similar mechanism of action, but further studies are needed to confirm this.

We consider that compound **6** is a selective H_4_R antagonist and has no significant affinity to H_1_R (in a preliminary study, compound **6** showed 0% activity at 10 µM) and histamine H_3_ receptor (data based on our previous experiments with H_4_R ligands [44]).

To conclude, compound **6** is a promising lead structure for structural modifications, further in vitro studies (e.g., metabolic stability), and in vivo studies to test its efficacy in other pain and itch models, and to try to find the mechanism of its anti-inflammatory effect.

## Figures and Tables

**Table 3:** Calculated pK_b_ and bias factors for tested ligands ^a^.

No	pK_b β-arrestin_ ± SEM	pK_b cAMP_ ± SEM	Bias Factor (pK_b β-arrestin_–pK_b cAMP)_ ± SEM
**2**	7.32 ± 0.05	6.52 ± 0.03	0.8 ± 0.1
**3**	7.78 ± 0.06	7.43 ± 0.01	0.4 ± 0.1
**4**	7.94 ± 0.01	7.44 ± 0.01	0.5 ± 0.0
**5**	7.78 ± 0.04	7.12 ± 0.05	0.7 ± 0.1
**6**	8.79 ± 0.03	8.45 ± 0.07	0.3 ± 0.1
**7**	8.63 ± 0.04	7.94 ± 0.03	0.7 ± 0.1
**TR-AF-49**	7.95 ± 0.04	7.02 ± 0.02	0.9 ± 0.0
**8**	8.17 ± 0.06	6.58 ± 0.02	1.6 ± 0.1
**10**	7.87 ± 0.03	6.8 ± 0.01	1.1 ± 0.0
**11**	7.8 ± 0.07	6.65 ± 0.03	1.2 ± 0.1
**12**	8.21 ± 0.08	7.33 ± 0.03	0.9 ± 0.1
**14**	8.41 ± 0.05	7.86 ± 0.18	0.6 ± 0.2
**16**	7.63 ± 0.12	6.66 ± 0.09	1.0 ± 0.1
**17**	6.93 ± 0.07	6.59 ± 0.04	0.3 ± 0.1
**18**	7.96 ± 0.02	7.19 ± 0.11	0.8 ± 0.1
**19**	8.25 ± 0.09	6.84 ± 0.08	1.4 ± 0.1
JNJ7777120	8.04 ± 0.06	7.76 ± 0.02	0.3 ± 0.1
Thioperamide	7.47 ± 0.08	6.8 ± 0.03	0.7 ± 0.1

^a^ Bias factor above 0 suggests ligand bias towards the beta-arrestin pathway while a bias factor below 0 suggests ligand bias toward the cAMP pathway. Blue highlights the results with the highest bias factors for the ß-arrestin pathway (bias factor ≥ 1).

**Table 4:** Permeability coefficient and mass retention of compound **6**.

Compound	P*_e_* ^1,2^ [10^−6^ cm/s] ± SD	Mass Retention (R)
**6**	12.26 ± 0.31	3.15%
Caffeine	9.78 ± 1.75	1.54%

^1^ tested in triplicate; ^2^ for permeable compounds P*_e_* is higher than 1.5 × 10^−6^cm/s.

## Data Availability

Not applicable.

## Supplementary Materials

The following supporting information can be downloaded at: https://www.mdpi.com/article/10.3390/molecules28104199/s1, Spectral information (^1^H-NMR and ^13^C-NMR) of synthesized compounds.

## Sample Availability

Samples of the compounds could be available from the authors upon request.
