# Supplementary material for: Trisubstituted 1,3,5-Triazines as Histamine H4 Receptor Antagonists with Promising Activity In Vivo
Source: Molecules. 2023 May 19;28(10):4199. doi: 10.3390/molecules28104199 (PMC10224356; doi:10.3390/molecules28104199)
Supplement: Supplementary file 1 [file molecules-28-04199-s001.zip › molecules-2344770-supplementary.pptx]

## Slide 1
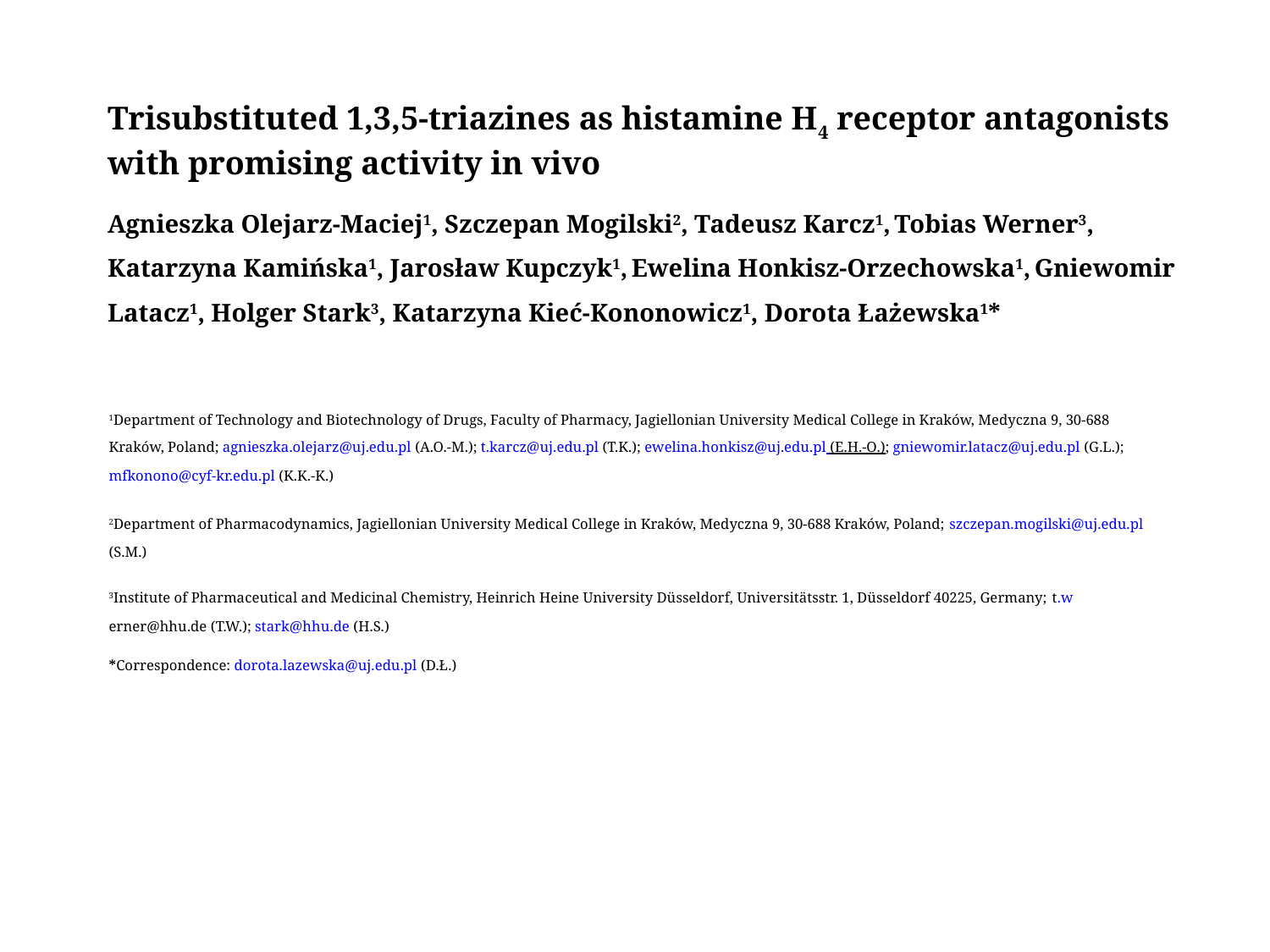

Trisubstituted 1,3,5-triazines as histamine H4 receptor antagonists with promising activity in vivo
Agnieszka Olejarz-Maciej1, Szczepan Mogilski2, Tadeusz Karcz1, Tobias Werner3, Katarzyna Kamińska1, Jarosław Kupczyk1, Ewelina Honkisz-Orzechowska1, Gniewomir Latacz1, Holger Stark3, Katarzyna Kieć-Kononowicz1, Dorota Łażewska1*
1Department of Technology and Biotechnology of Drugs, Faculty of Pharmacy, Jagiellonian University Medical College in Kraków, Medyczna 9, 30-688 Kraków, Poland; agnieszka.olejarz@uj.edu.pl (A.O.-M.); t.karcz@uj.edu.pl (T.K.); ewelina.honkisz@uj.edu.pl (E.H.-O.); gniewomir.latacz@uj.edu.pl (G.L.); mfkonono@cyf-kr.edu.pl (K.K.-K.)
2Department of Pharmacodynamics, Jagiellonian University Medical College in Kraków, Medyczna 9, 30-688 Kraków, Poland; szczepan.mogilski@uj.edu.pl (S.M.)
3Institute of Pharmaceutical and Medicinal Chemistry, Heinrich Heine University Düsseldorf, Universitätsstr. 1, Düsseldorf 40225, Germany; t.werner@hhu.de (T.W.); stark@hhu.de (H.S.)
*Correspondence: dorota.lazewska@uj.edu.pl (D.Ł.)

## Slide 2
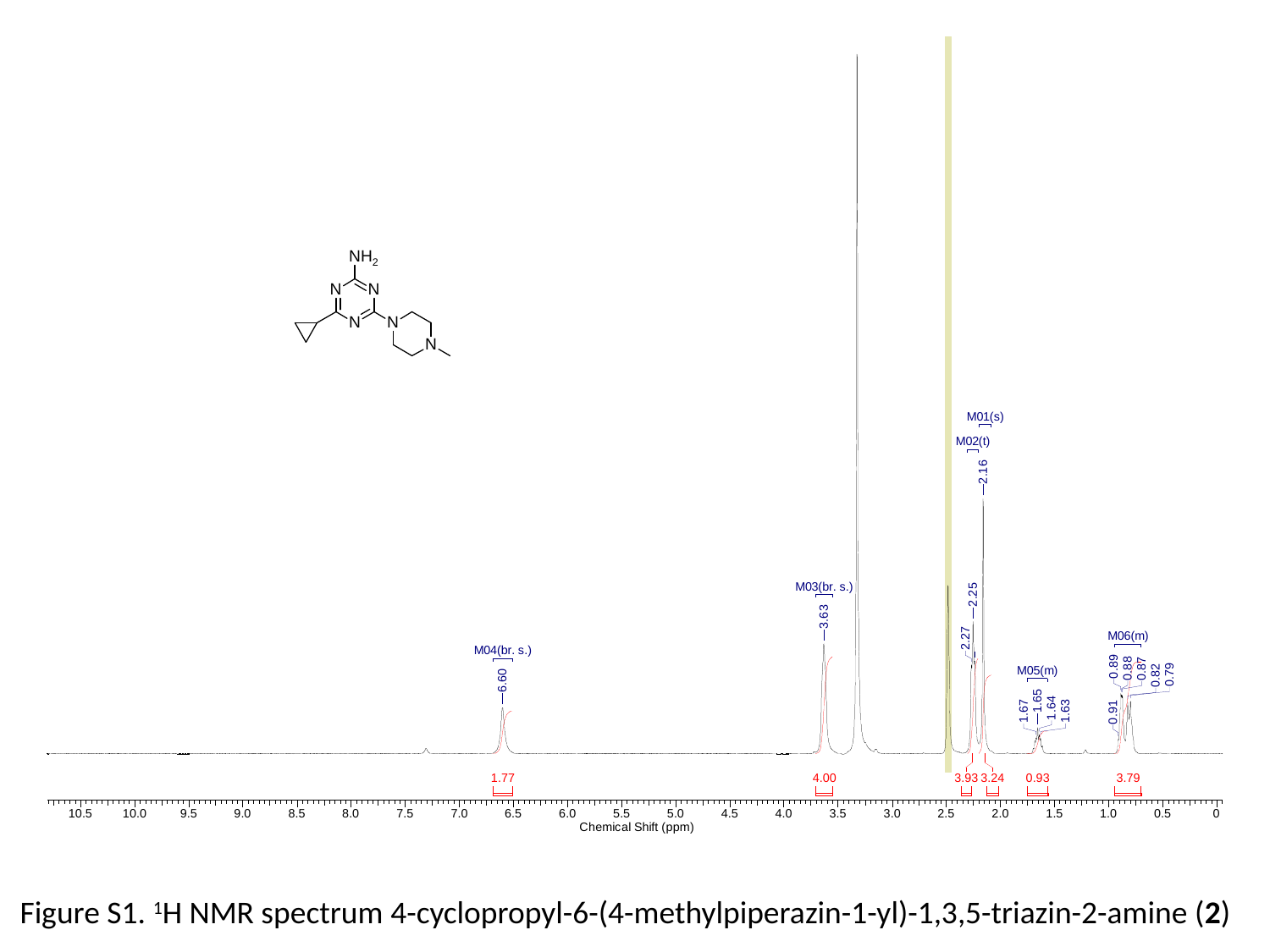

Figure S1. 1H NMR spectrum 4-cyclopropyl-6-(4-methylpiperazin-1-yl)-1,3,5-triazin-2-amine (2)

## Slide 3
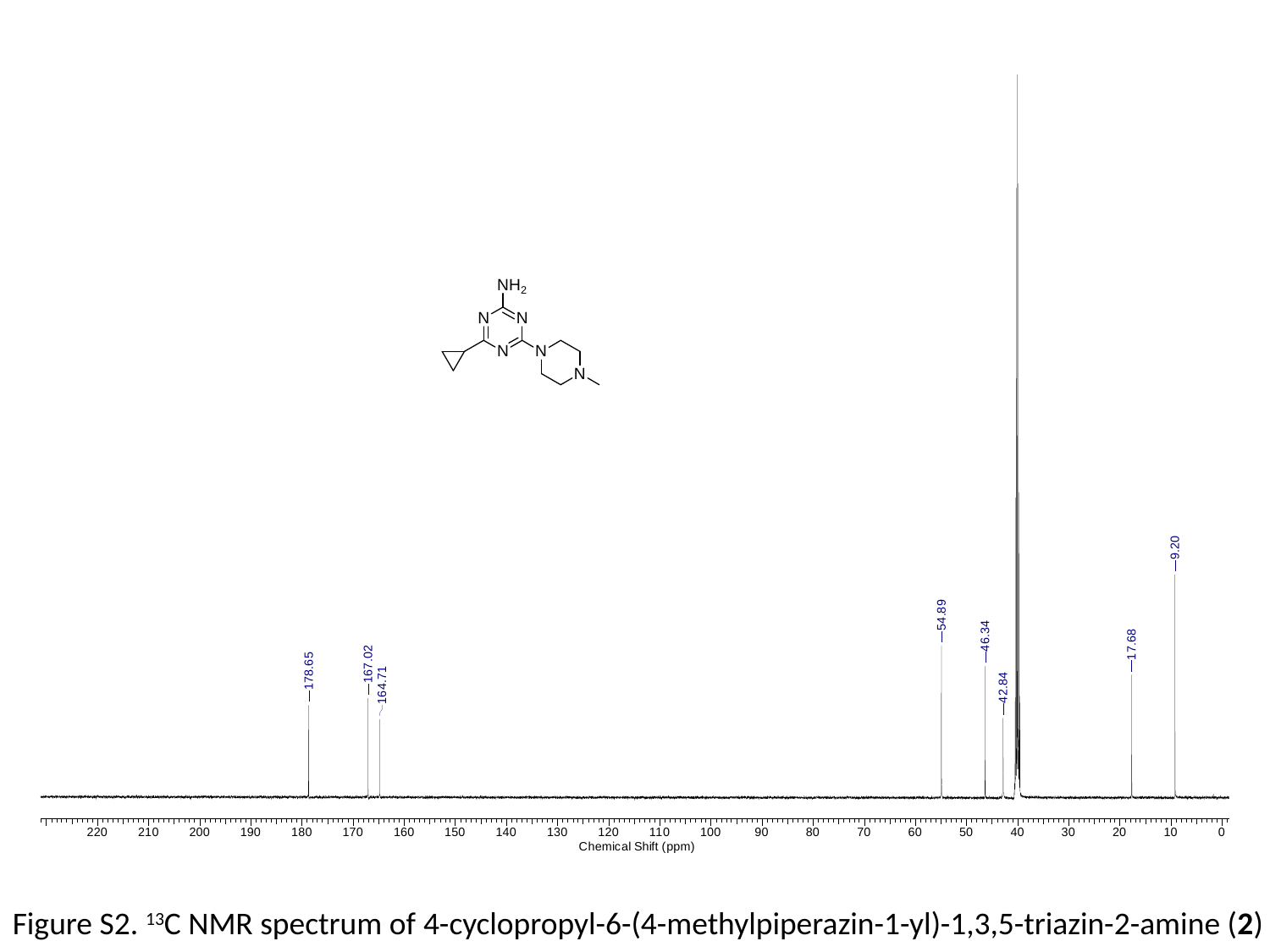

Figure S2. 13C NMR spectrum of 4-cyclopropyl-6-(4-methylpiperazin-1-yl)-1,3,5-triazin-2-amine (2)

## Slide 4
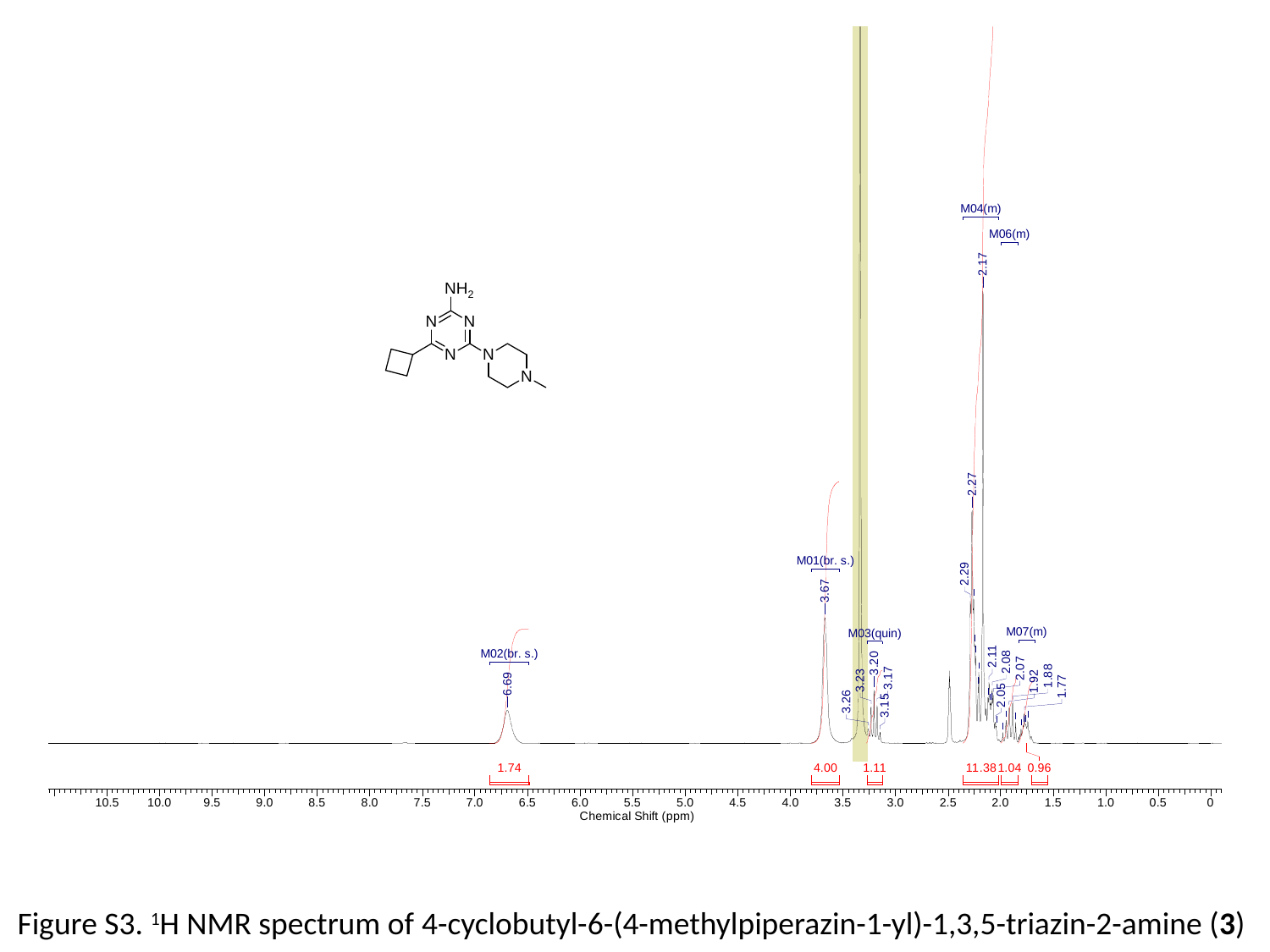

Figure S3. 1H NMR spectrum of 4-cyclobutyl-6-(4-methylpiperazin-1-yl)-1,3,5-triazin-2-amine (3)

## Slide 5
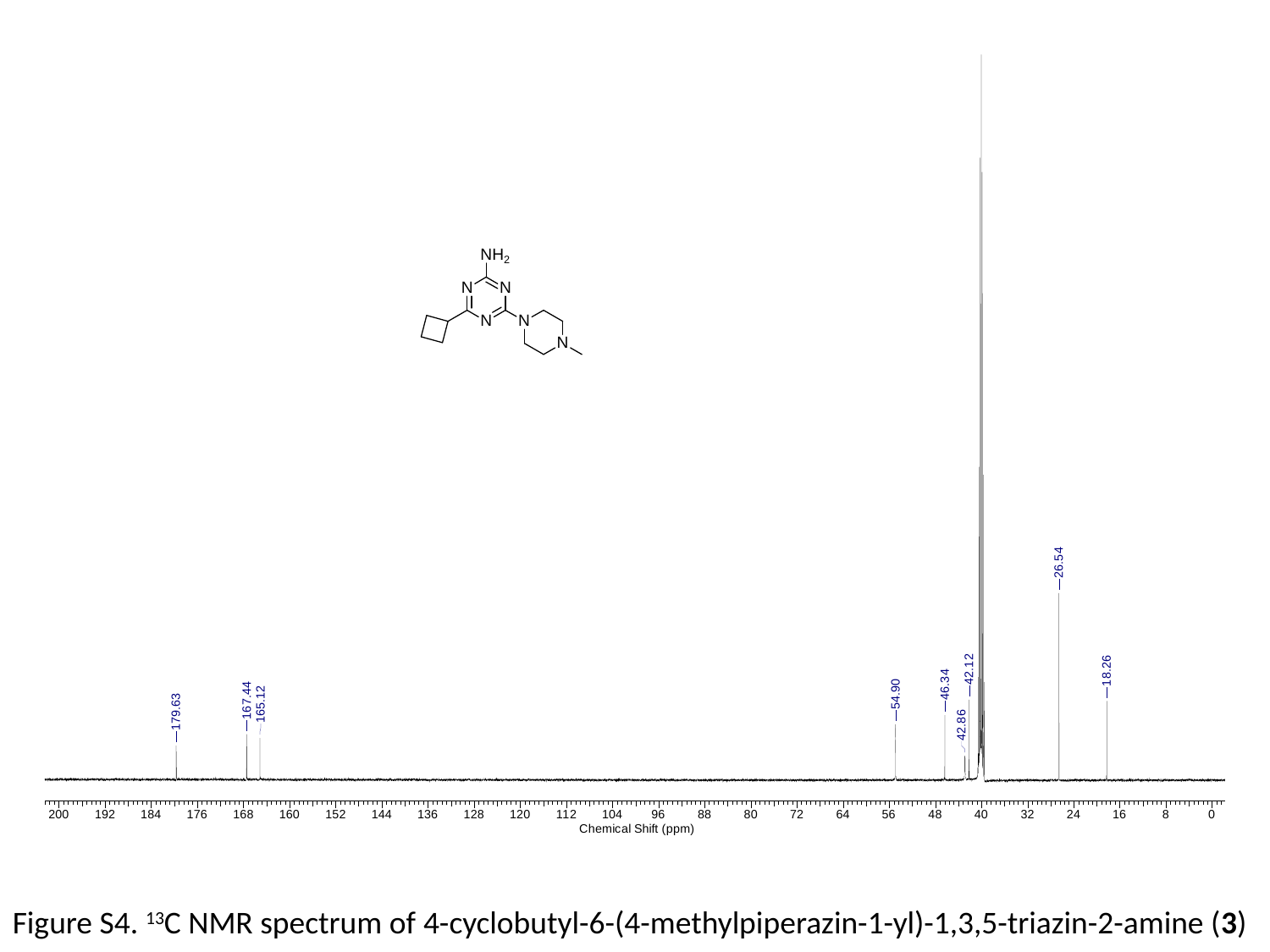

Figure S4. 13C NMR spectrum of 4-cyclobutyl-6-(4-methylpiperazin-1-yl)-1,3,5-triazin-2-amine (3)

## Slide 6
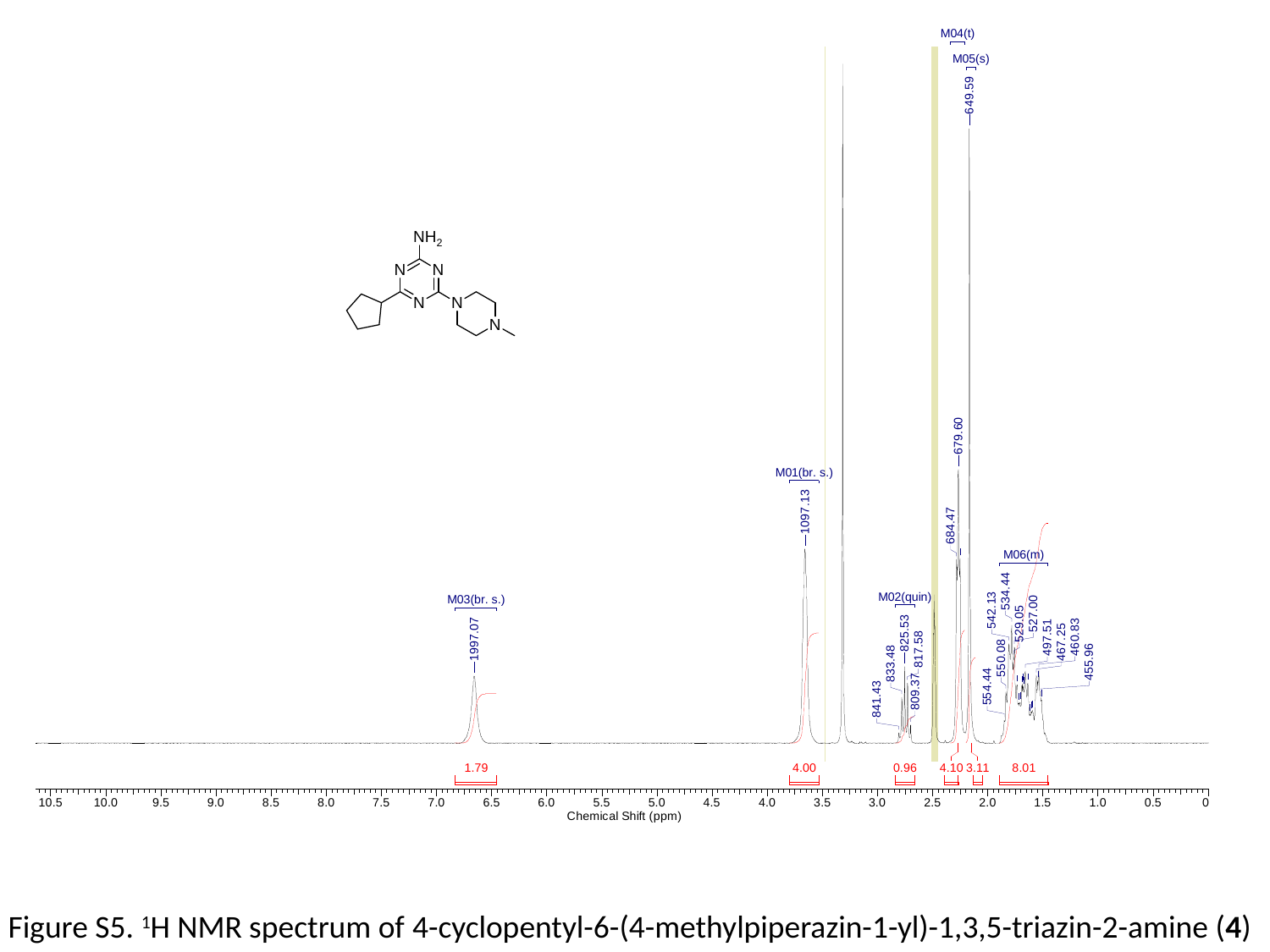

Figure S5. 1H NMR spectrum of 4-cyclopentyl-6-(4-methylpiperazin-1-yl)-1,3,5-triazin-2-amine (4)

## Slide 7
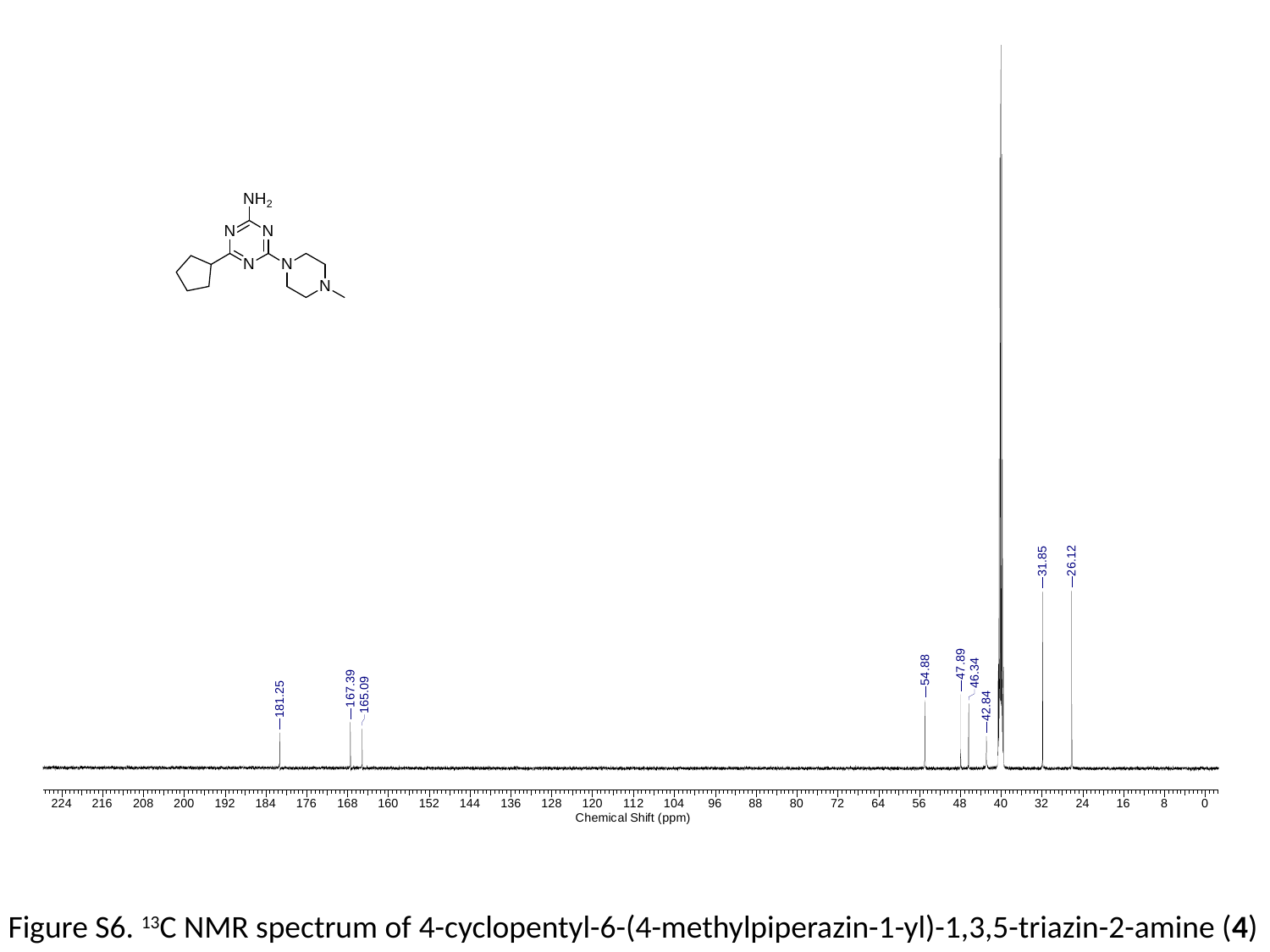

Figure S6. 13C NMR spectrum of 4-cyclopentyl-6-(4-methylpiperazin-1-yl)-1,3,5-triazin-2-amine (4)

## Slide 8
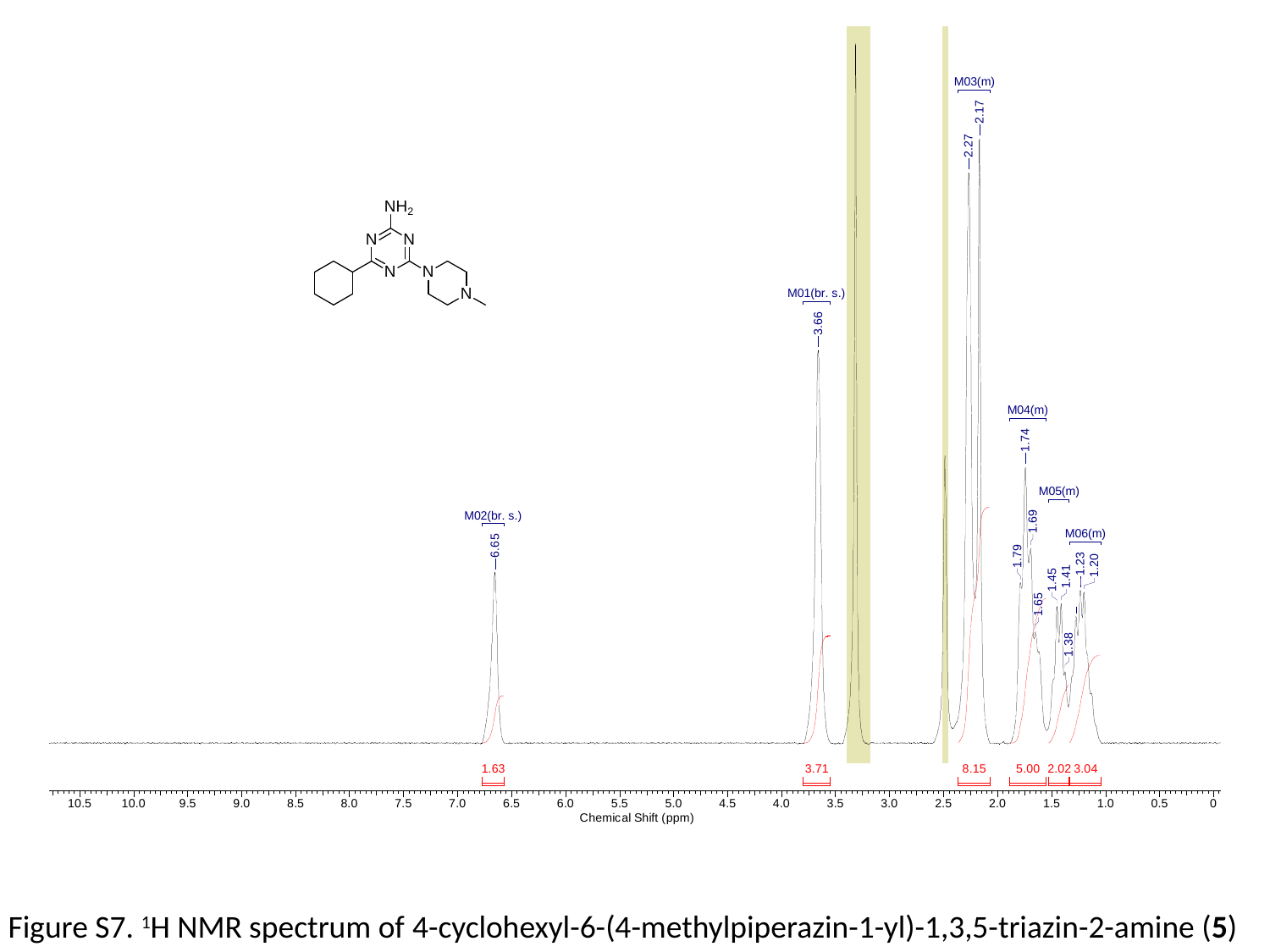

Figure S7. 1H NMR spectrum of 4-cyclohexyl-6-(4-methylpiperazin-1-yl)-1,3,5-triazin-2-amine (5)

## Slide 9
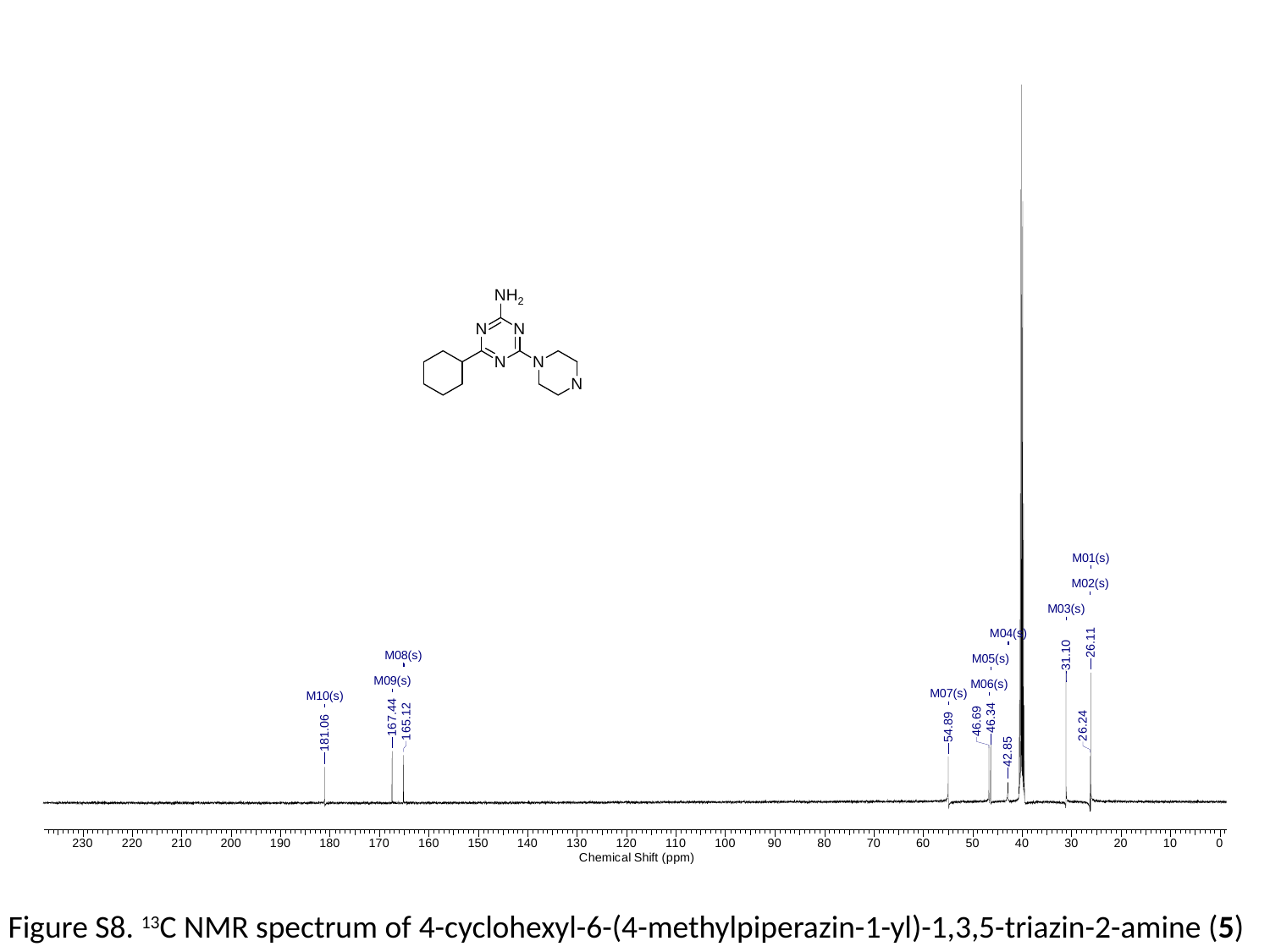

Figure S8. 13C NMR spectrum of 4-cyclohexyl-6-(4-methylpiperazin-1-yl)-1,3,5-triazin-2-amine (5)

## Slide 10
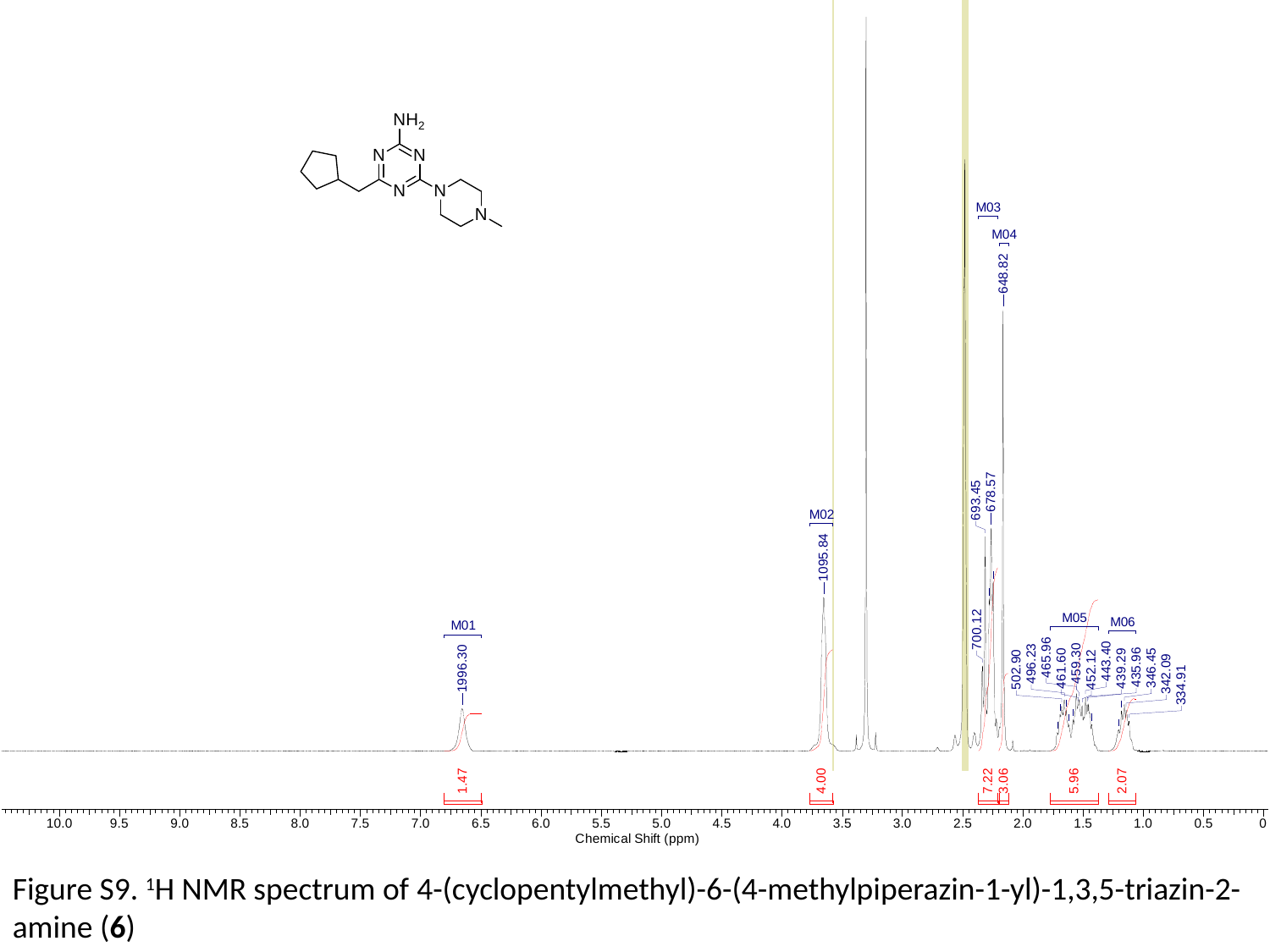

Figure S9. 1H NMR spectrum of 4-(cyclopentylmethyl)-6-(4-methylpiperazin-1-yl)-1,3,5-triazin-2-amine (6)

## Slide 11
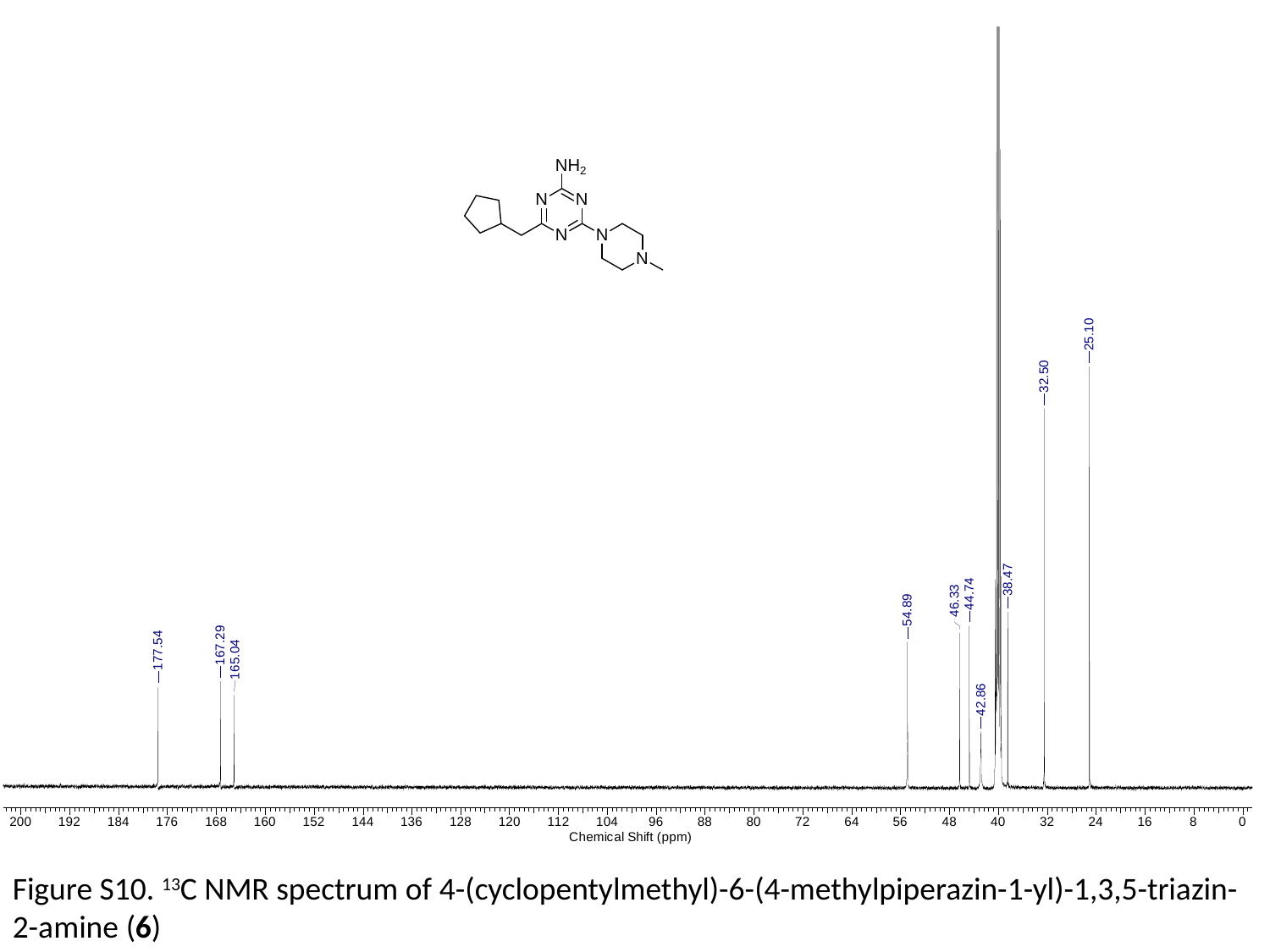

Figure S10. 13C NMR spectrum of 4-(cyclopentylmethyl)-6-(4-methylpiperazin-1-yl)-1,3,5-triazin-2-amine (6)

## Slide 12
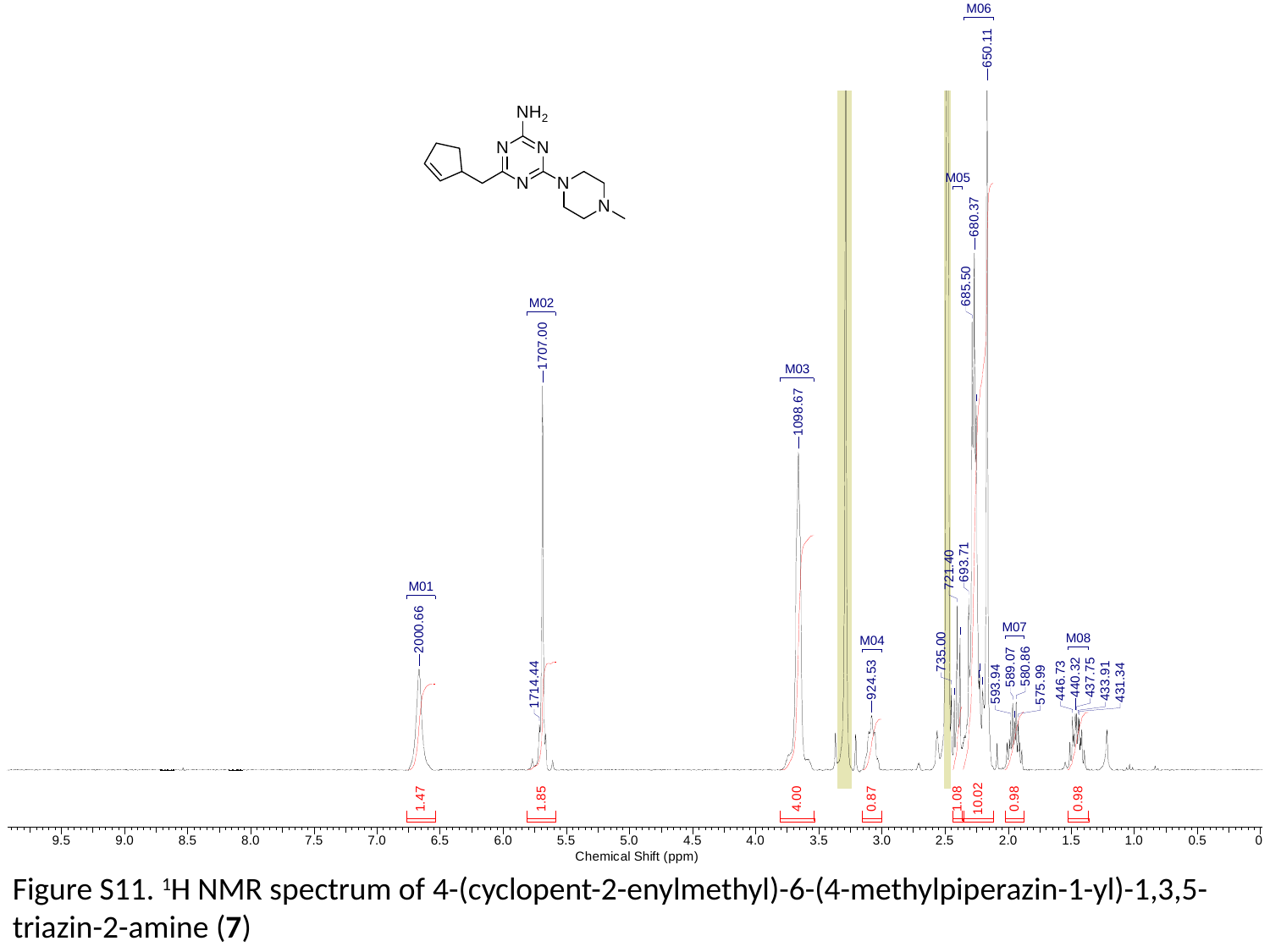

Figure S11. 1H NMR spectrum of 4-(cyclopent-2-enylmethyl)-6-(4-methylpiperazin-1-yl)-1,3,5-triazin-2-amine (7)

## Slide 13
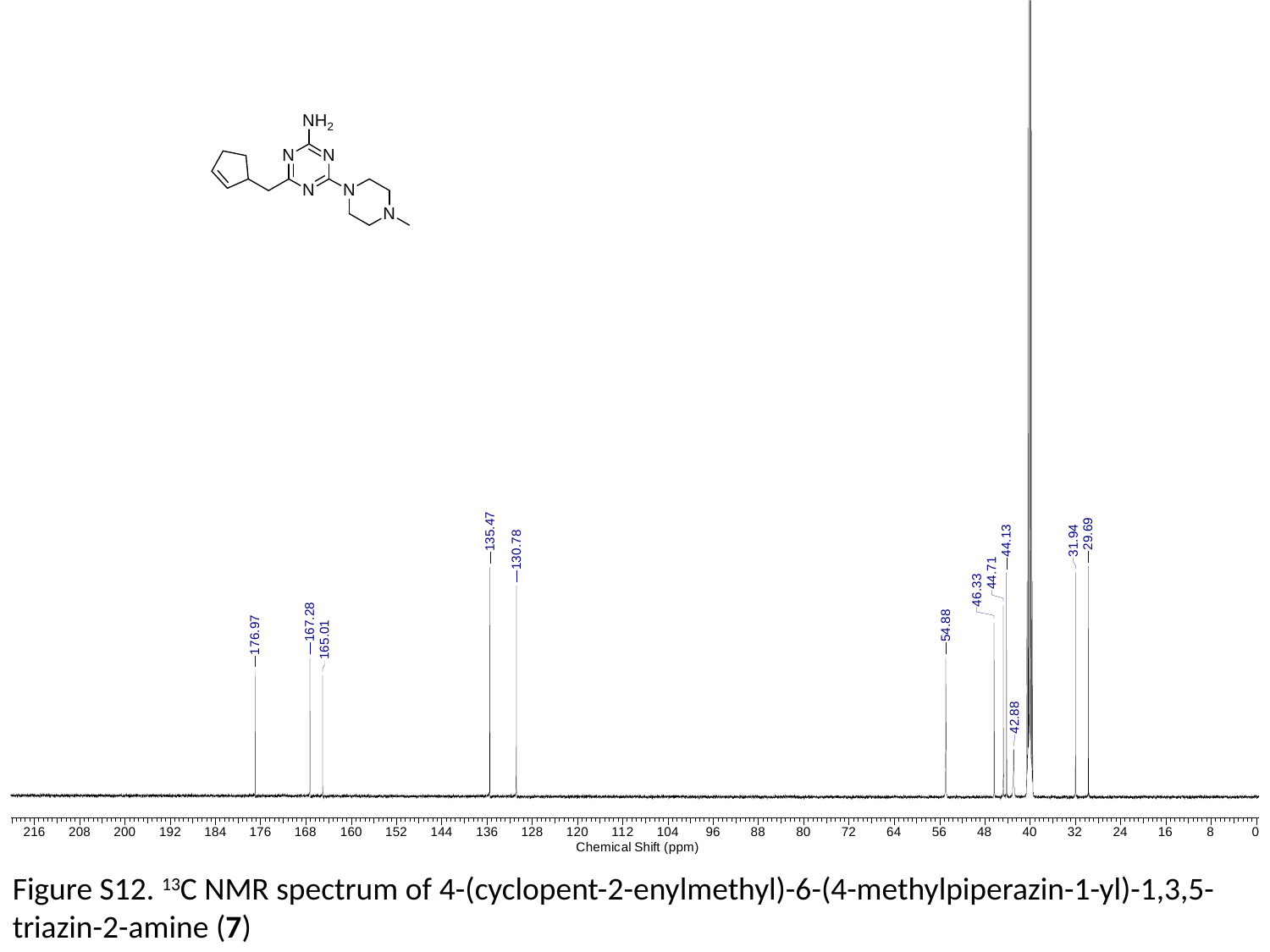

Figure S12. 13C NMR spectrum of 4-(cyclopent-2-enylmethyl)-6-(4-methylpiperazin-1-yl)-1,3,5-triazin-2-amine (7)

## Slide 14
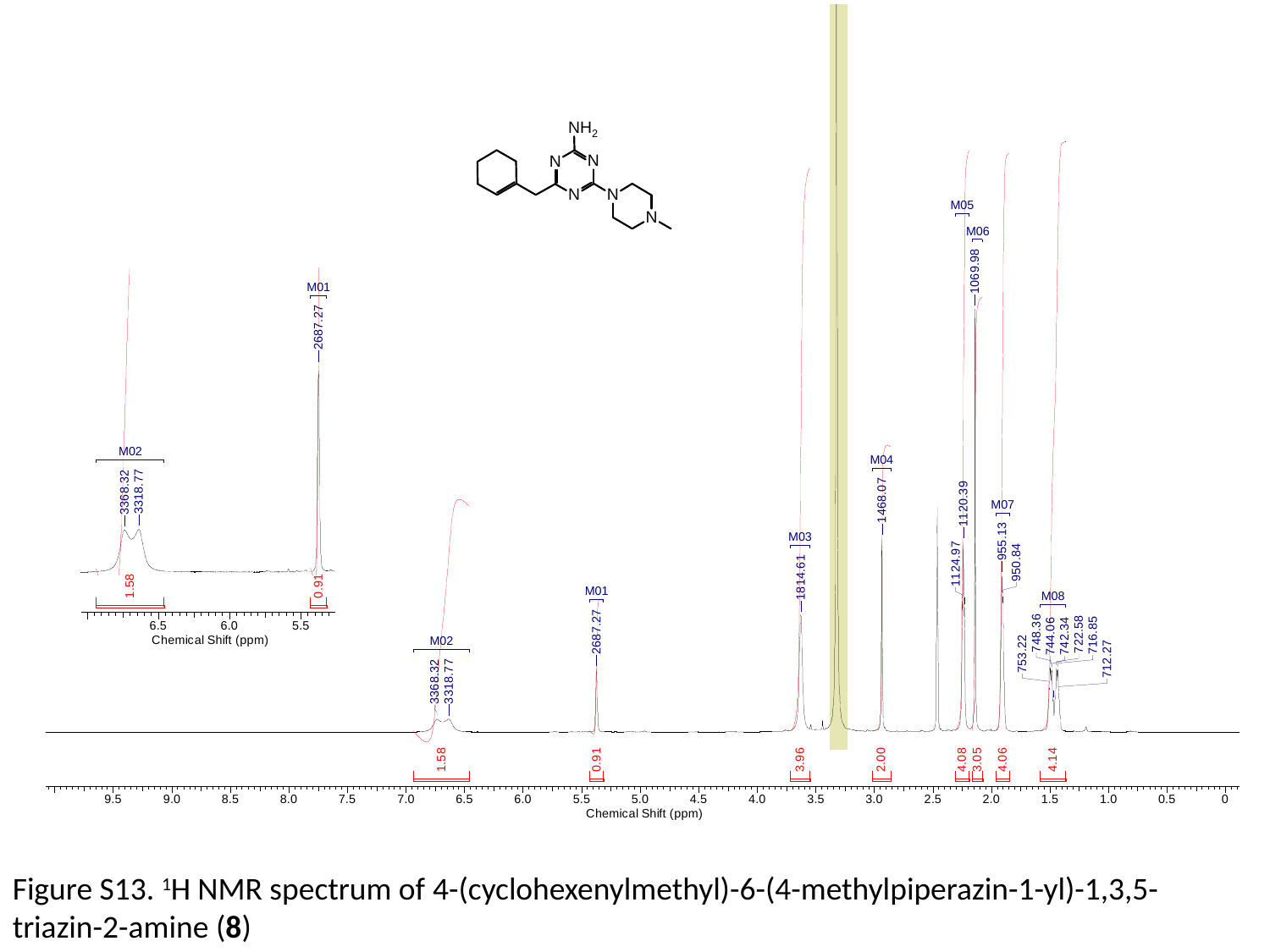

Figure S13. 1H NMR spectrum of 4-(cyclohexenylmethyl)-6-(4-methylpiperazin-1-yl)-1,3,5-triazin-2-amine (8)

## Slide 15
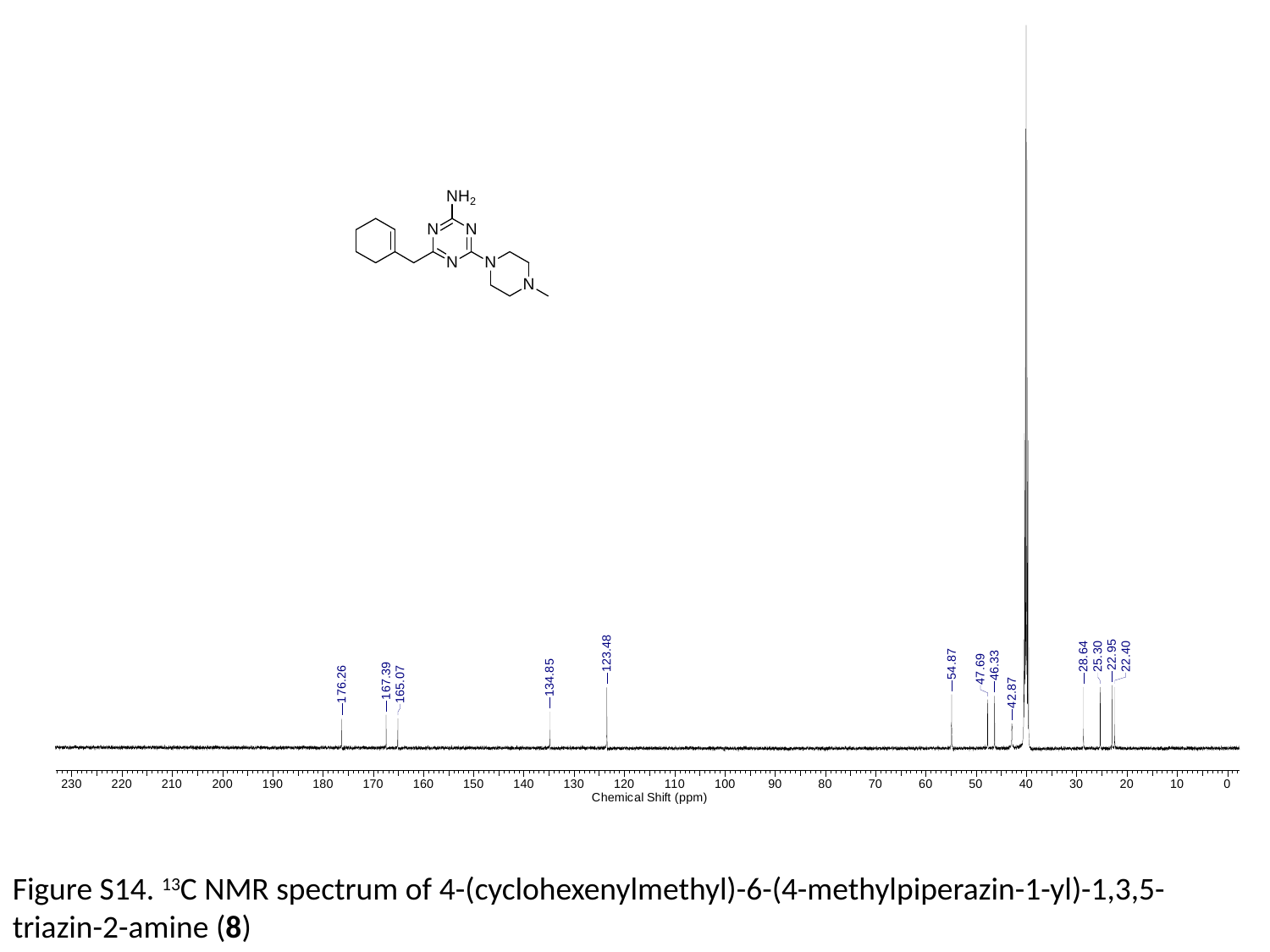

Figure S14. 13C NMR spectrum of 4-(cyclohexenylmethyl)-6-(4-methylpiperazin-1-yl)-1,3,5-triazin-2-amine (8)

## Slide 16
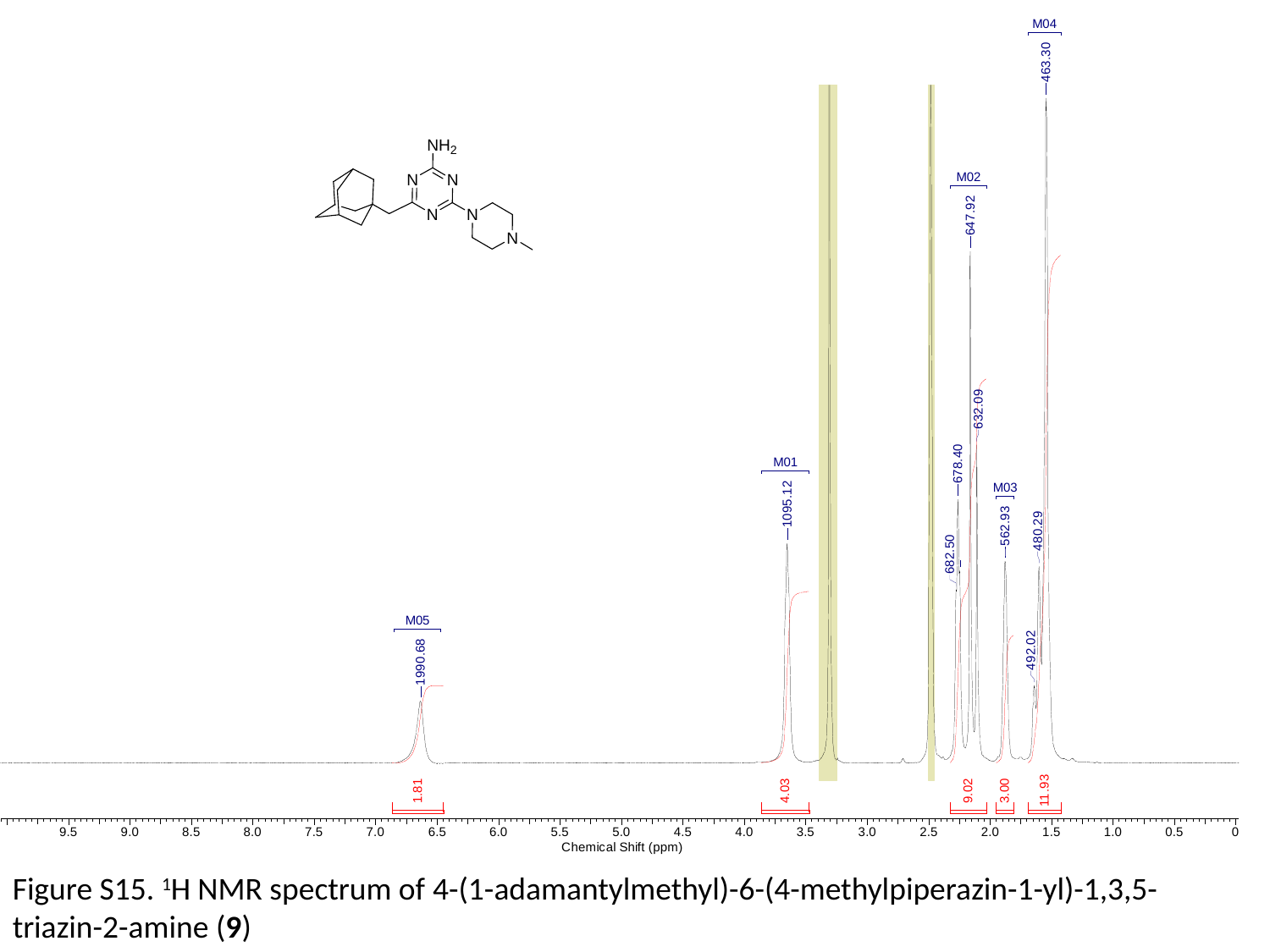

Figure S15. 1H NMR spectrum of 4-(1-adamantylmethyl)-6-(4-methylpiperazin-1-yl)-1,3,5-triazin-2-amine (9)

## Slide 17
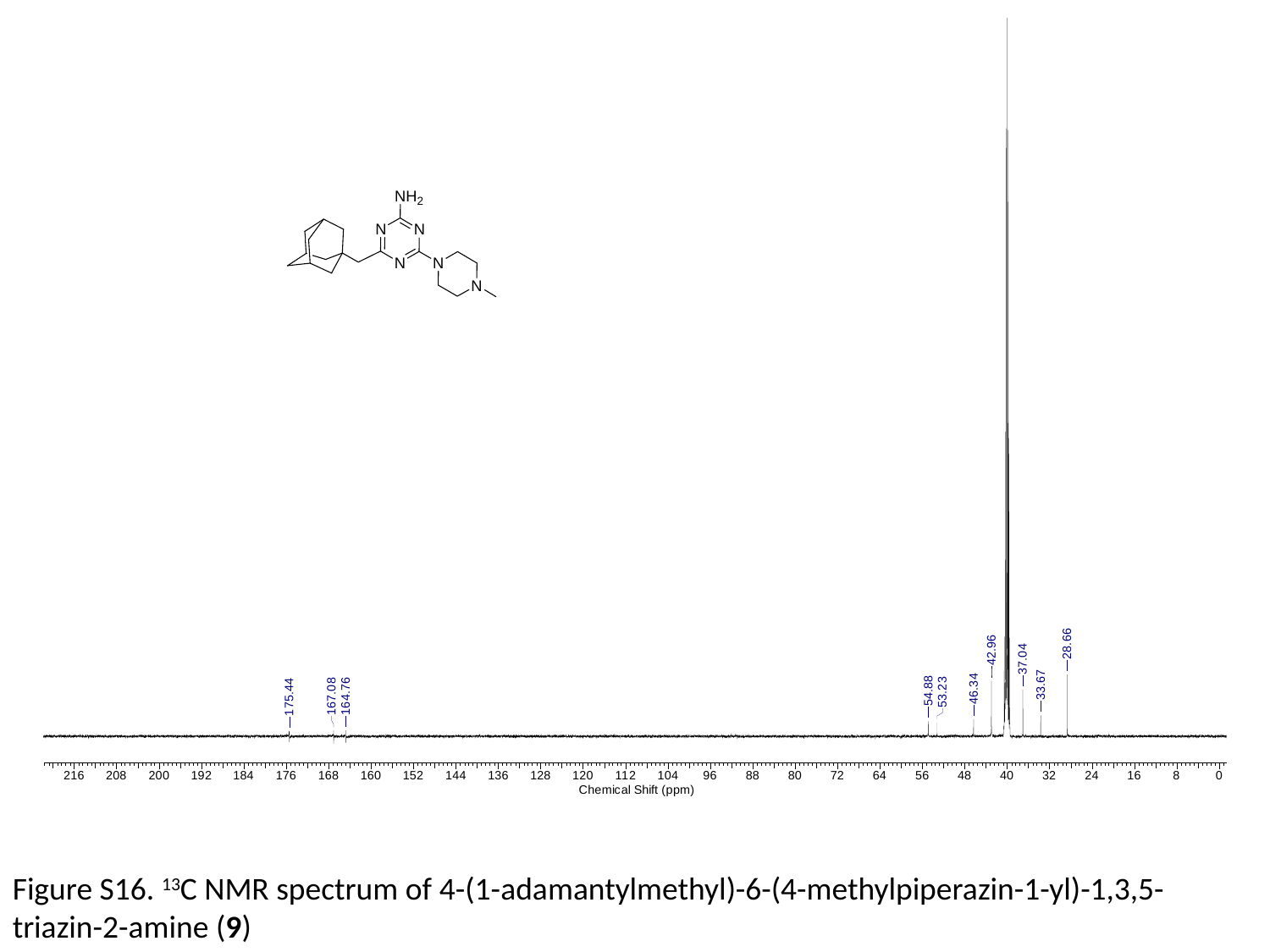

Figure S16. 13C NMR spectrum of 4-(1-adamantylmethyl)-6-(4-methylpiperazin-1-yl)-1,3,5-triazin-2-amine (9)

## Slide 18
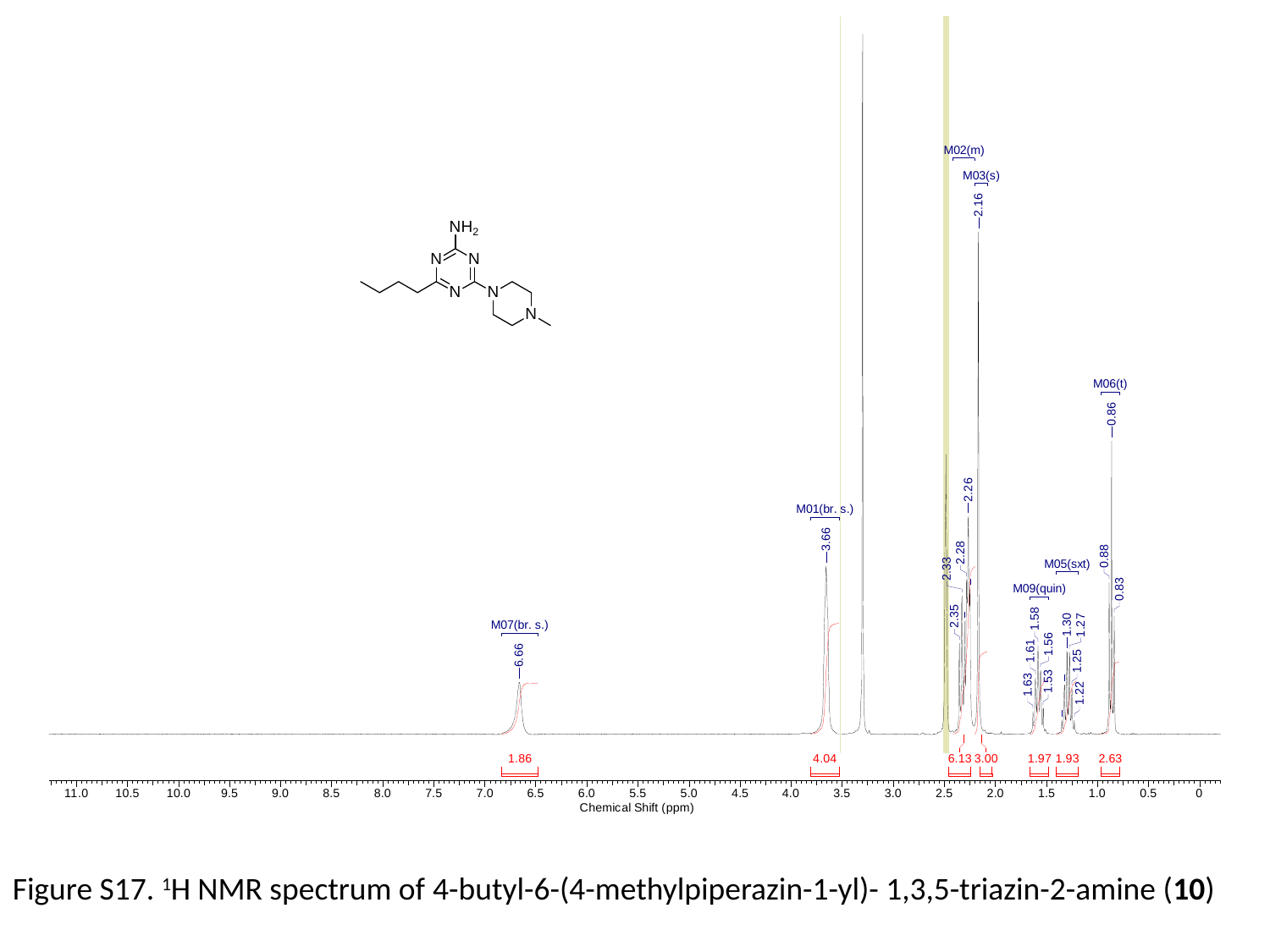

Figure S17. 1H NMR spectrum of 4-butyl-6-(4-methylpiperazin-1-yl)- 1,3,5-triazin-2-amine (10)

## Slide 19
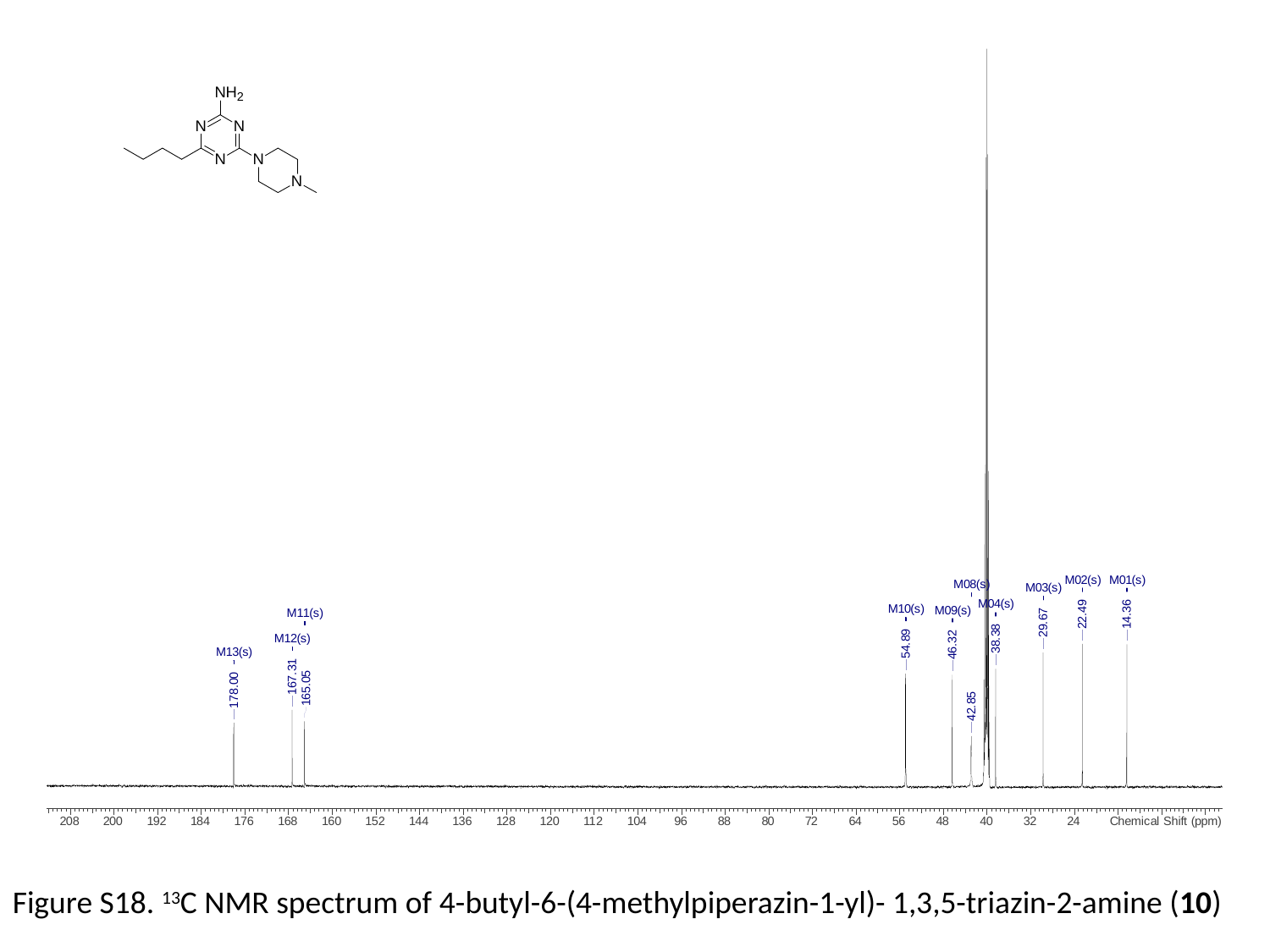

Figure S18. 13C NMR spectrum of 4-butyl-6-(4-methylpiperazin-1-yl)- 1,3,5-triazin-2-amine (10)

## Slide 20
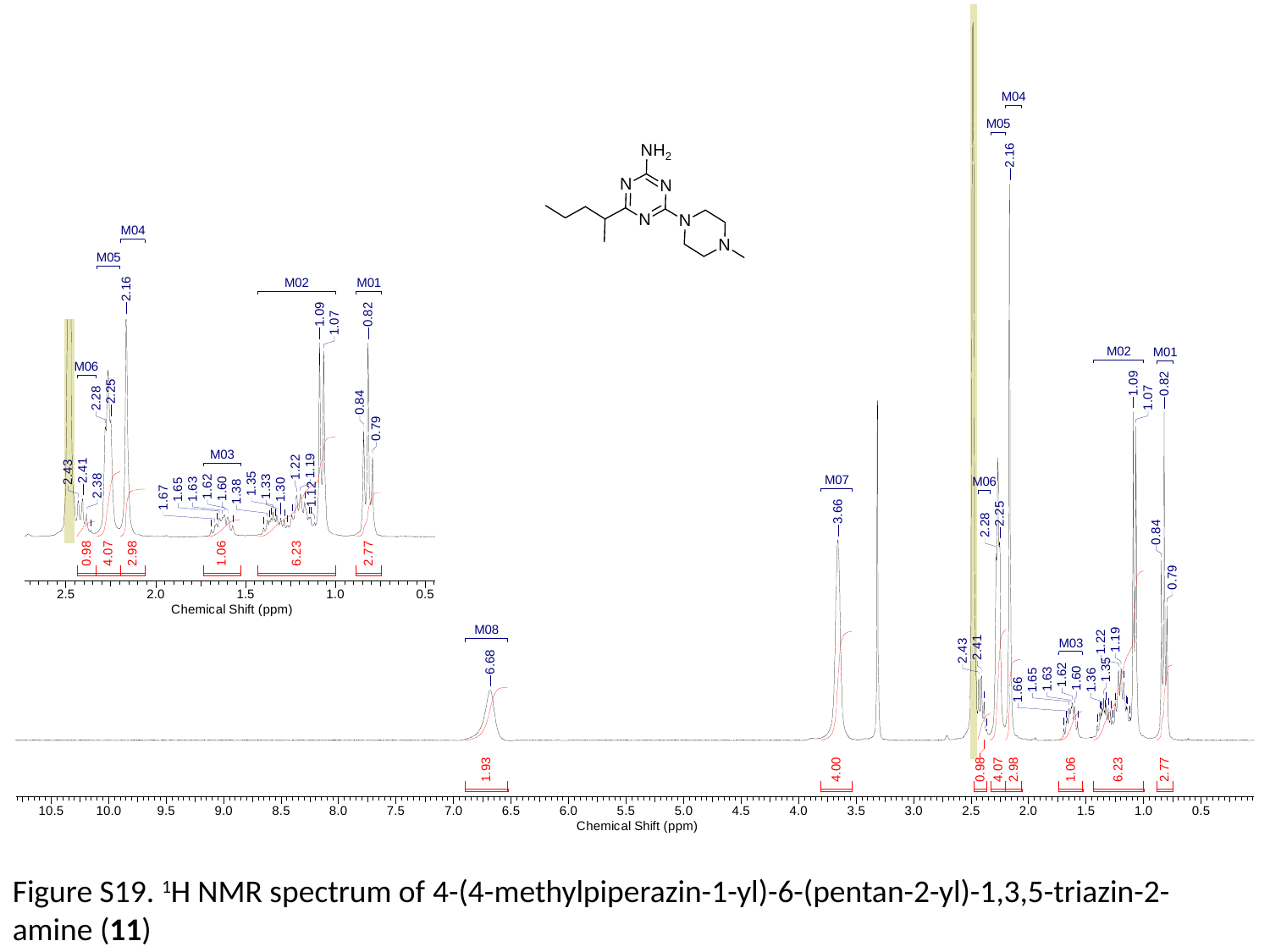

Figure S19. 1H NMR spectrum of 4-(4-methylpiperazin-1-yl)-6-(pentan-2-yl)-1,3,5-triazin-2-amine (11)

## Slide 21
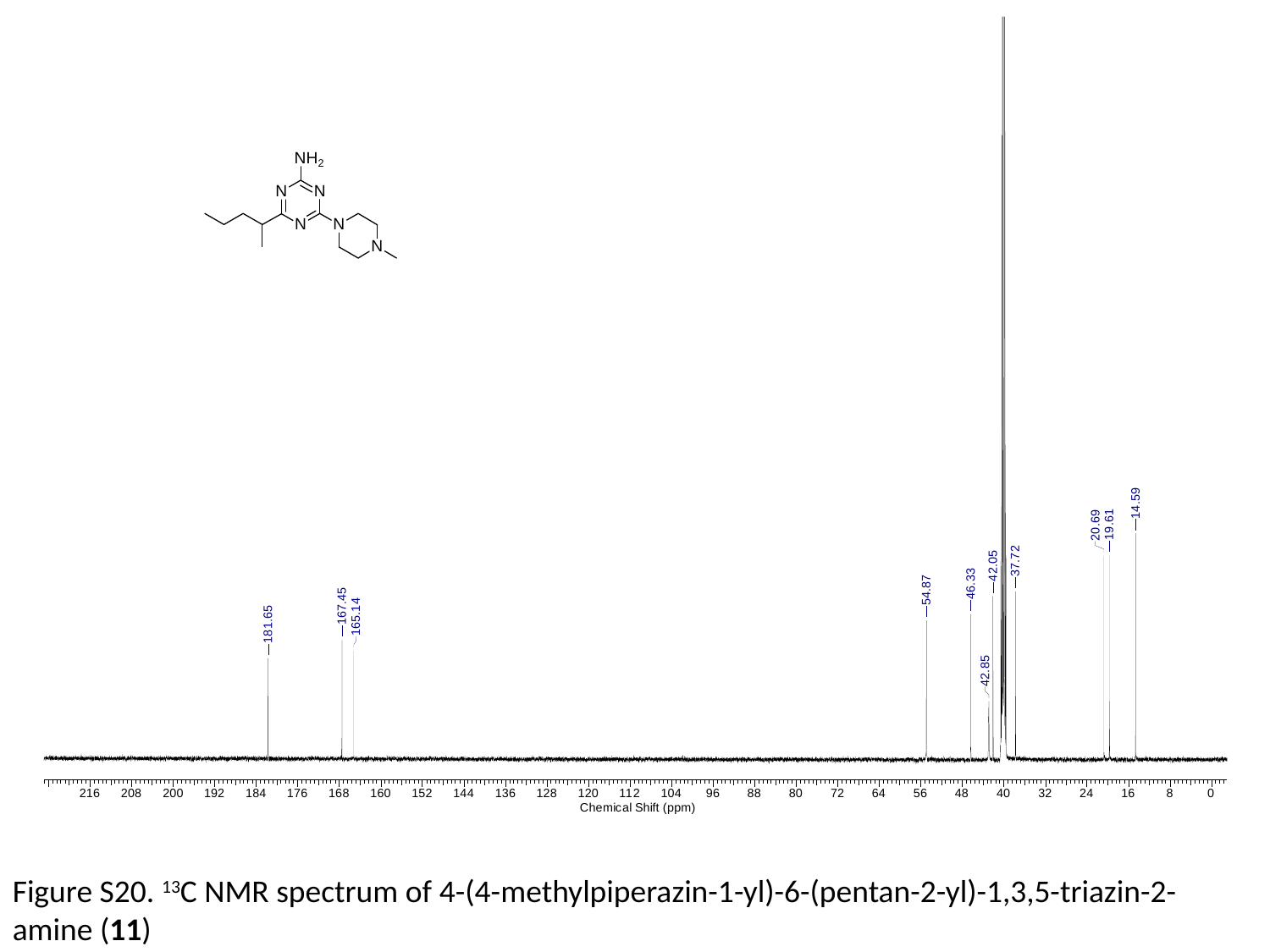

Figure S20. 13C NMR spectrum of 4-(4-methylpiperazin-1-yl)-6-(pentan-2-yl)-1,3,5-triazin-2-amine (11)

## Slide 22
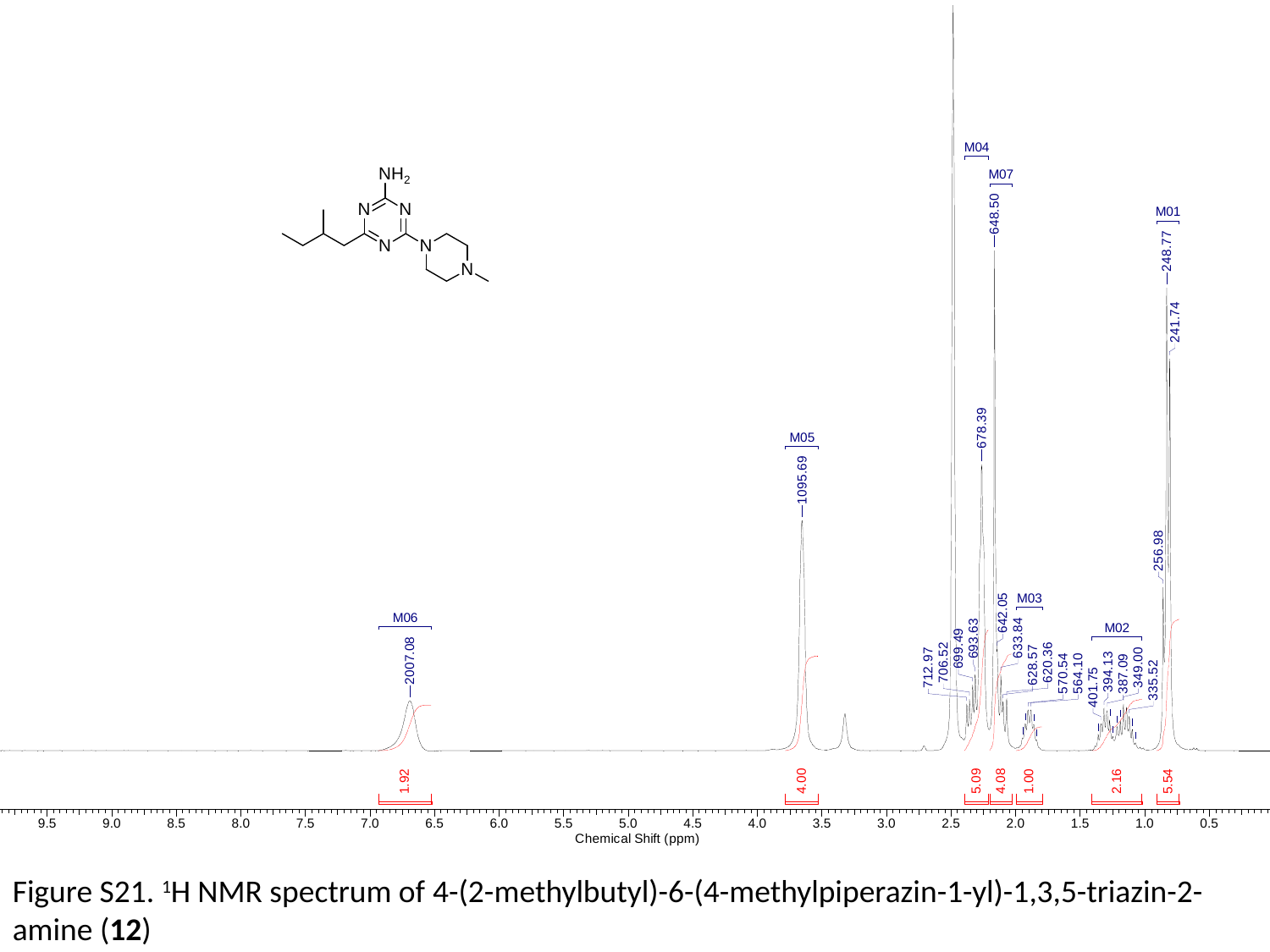

Figure S21. 1H NMR spectrum of 4-(2-methylbutyl)-6-(4-methylpiperazin-1-yl)-1,3,5-triazin-2-amine (12)

## Slide 23
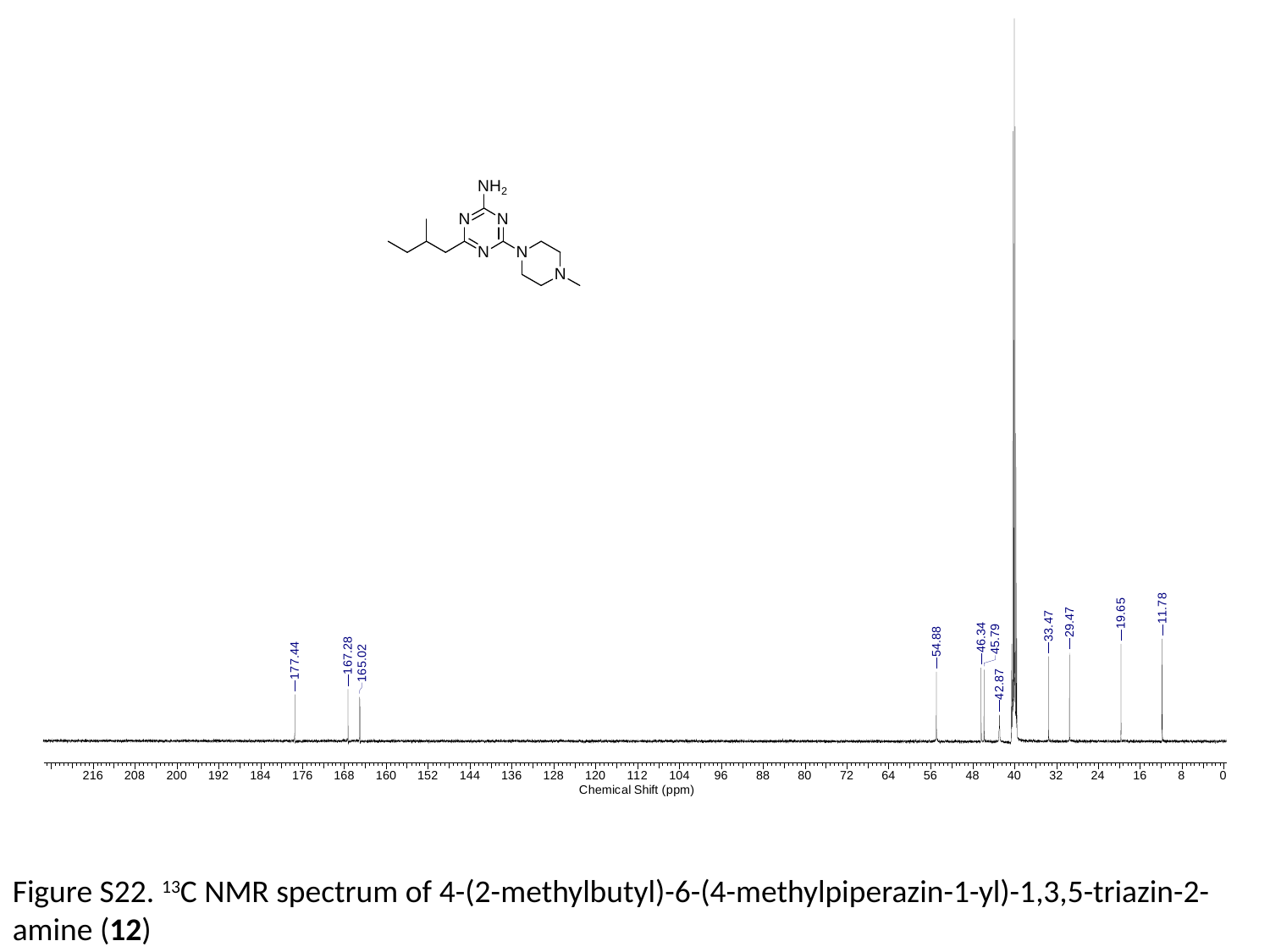

Figure S22. 13C NMR spectrum of 4-(2-methylbutyl)-6-(4-methylpiperazin-1-yl)-1,3,5-triazin-2-amine (12)

## Slide 24
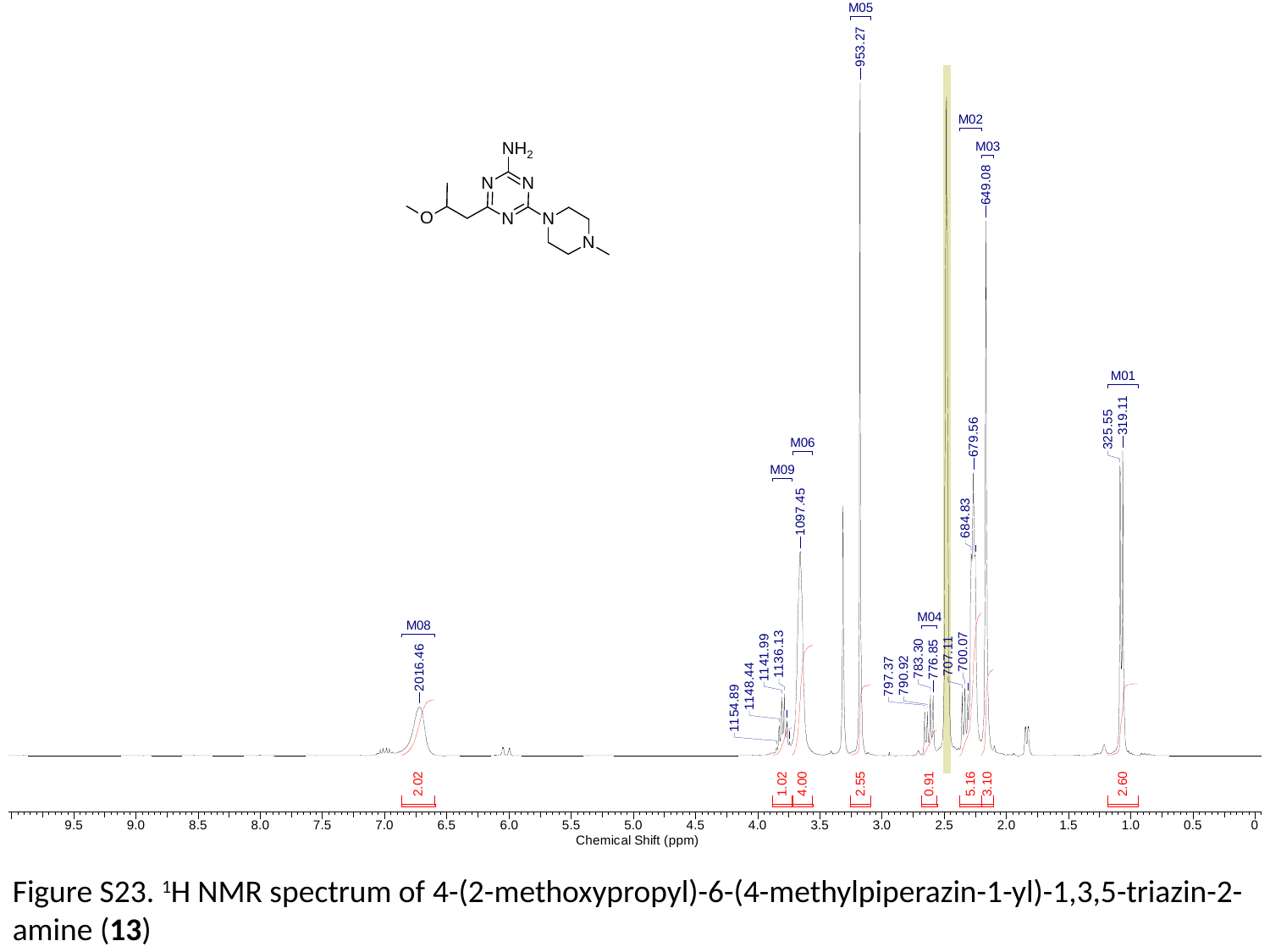

Figure S23. 1H NMR spectrum of 4-(2-methoxypropyl)-6-(4-methylpiperazin-1-yl)-1,3,5-triazin-2-amine (13)

## Slide 25
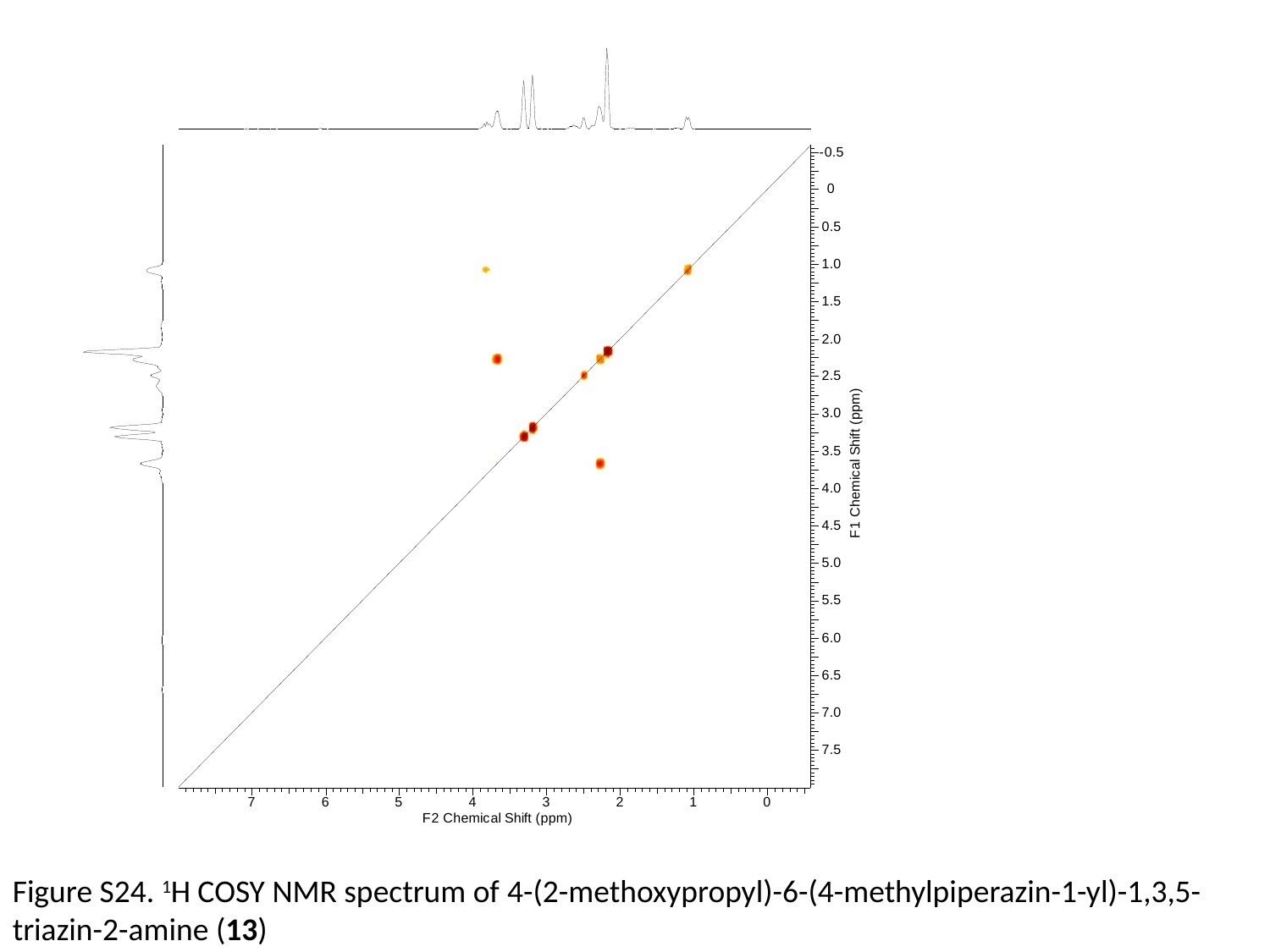

Figure S24. 1H COSY NMR spectrum of 4-(2-methoxypropyl)-6-(4-methylpiperazin-1-yl)-1,3,5-triazin-2-amine (13)

## Slide 26
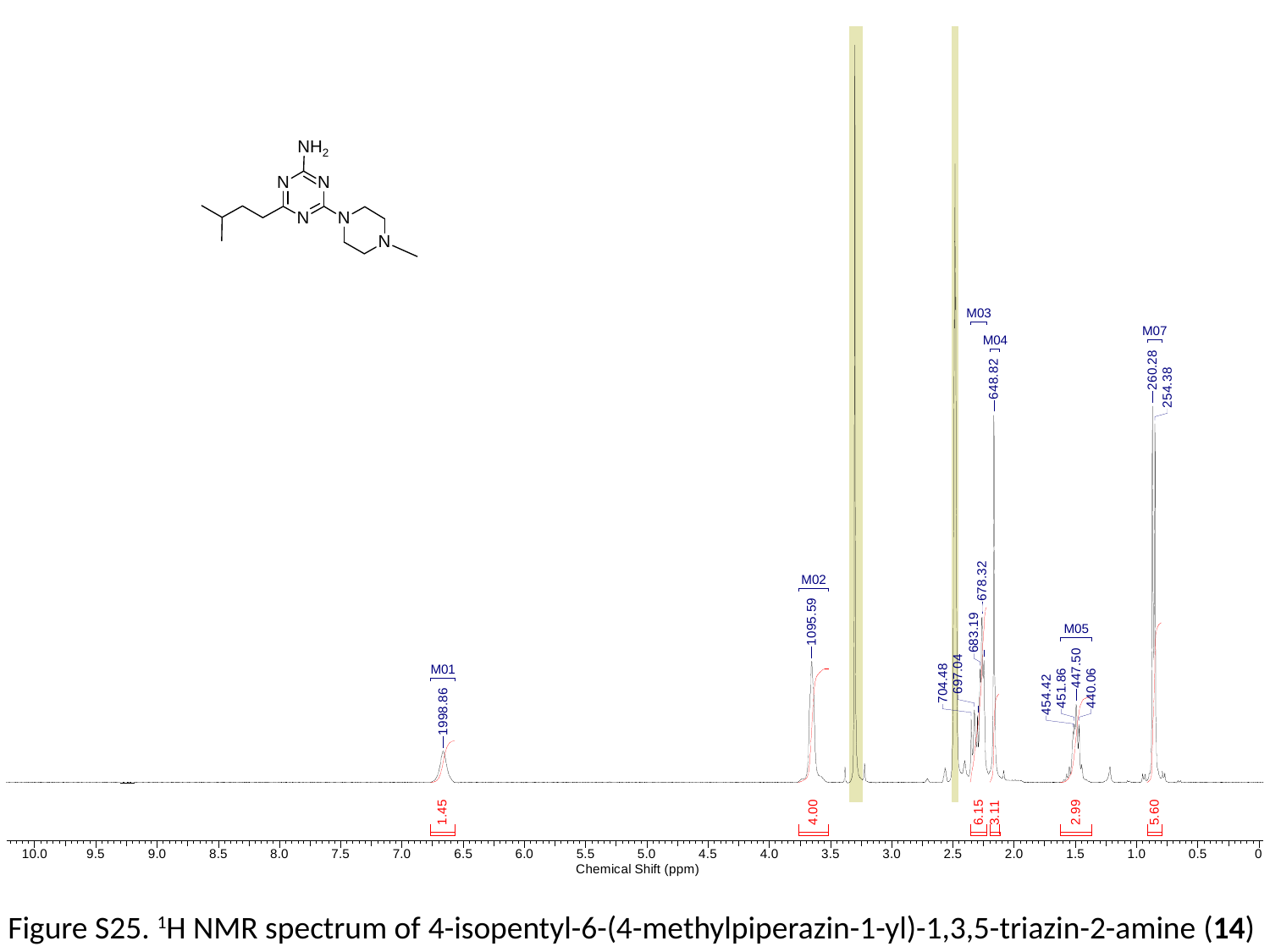

Figure S25. 1H NMR spectrum of 4-isopentyl-6-(4-methylpiperazin-1-yl)-1,3,5-triazin-2-amine (14)

## Slide 27
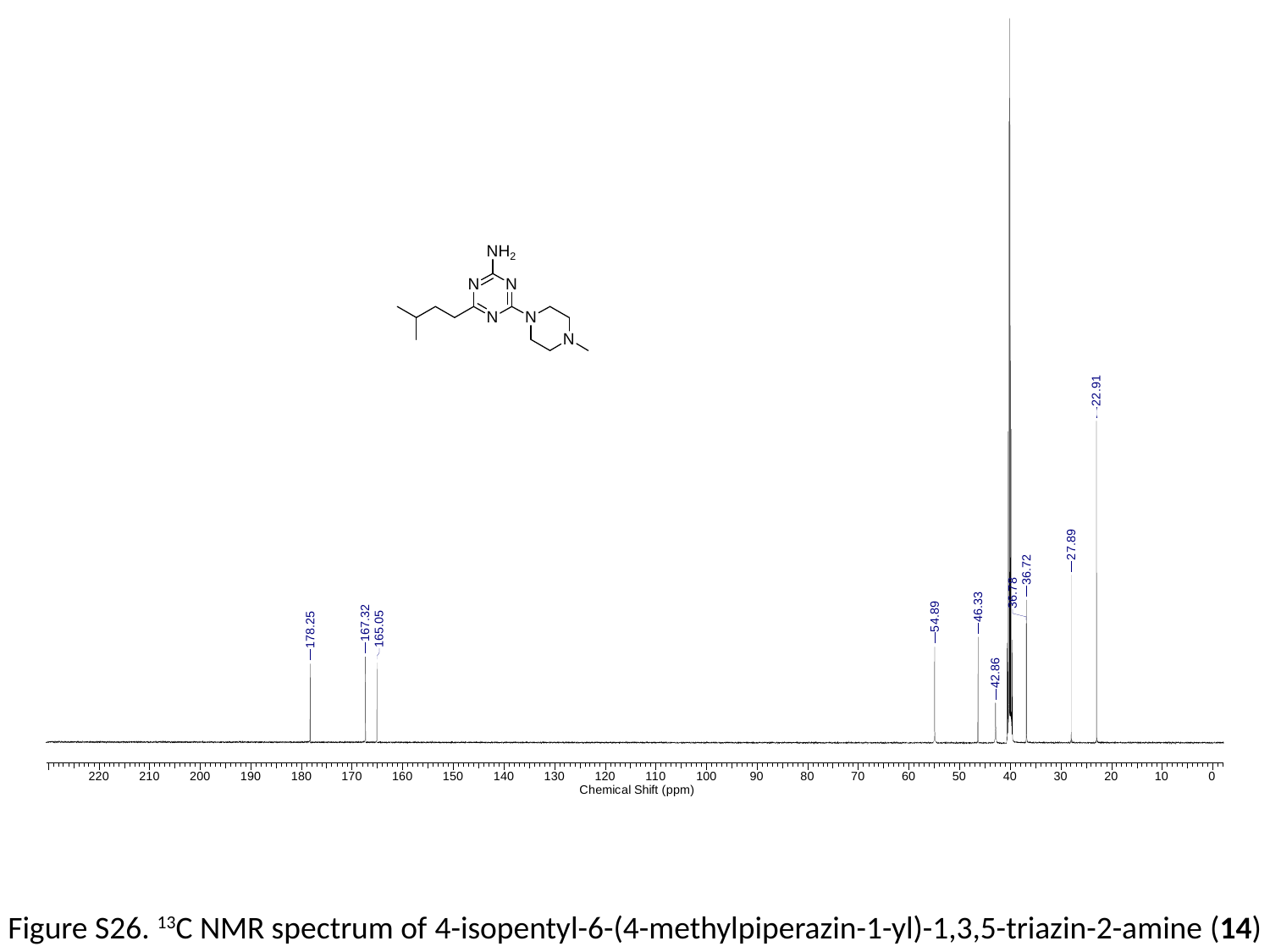

Figure S26. 13C NMR spectrum of 4-isopentyl-6-(4-methylpiperazin-1-yl)-1,3,5-triazin-2-amine (14)

## Slide 28
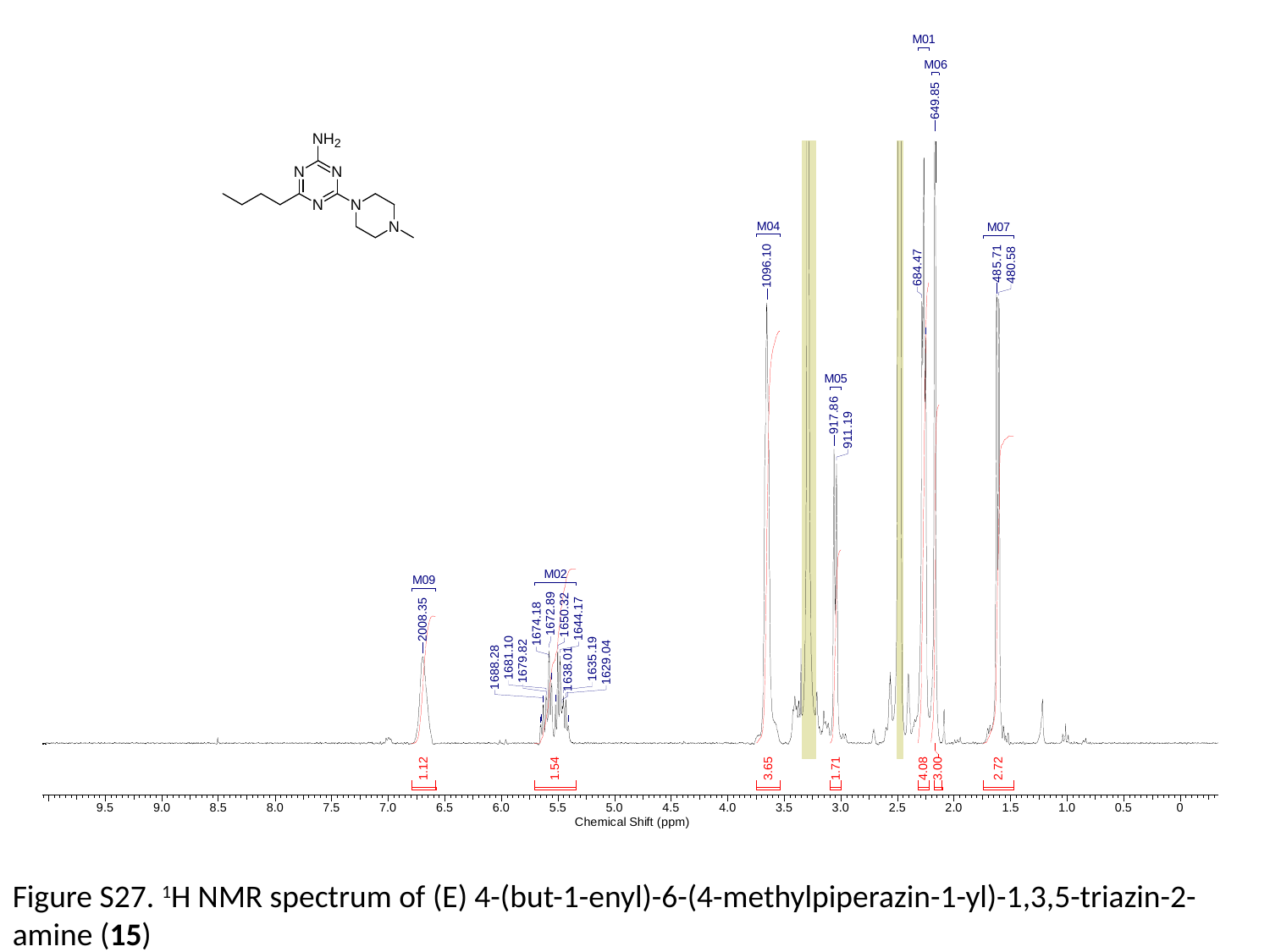

Figure S27. 1H NMR spectrum of (E) 4-(but-1-enyl)-6-(4-methylpiperazin-1-yl)-1,3,5-triazin-2-amine (15)

## Slide 29
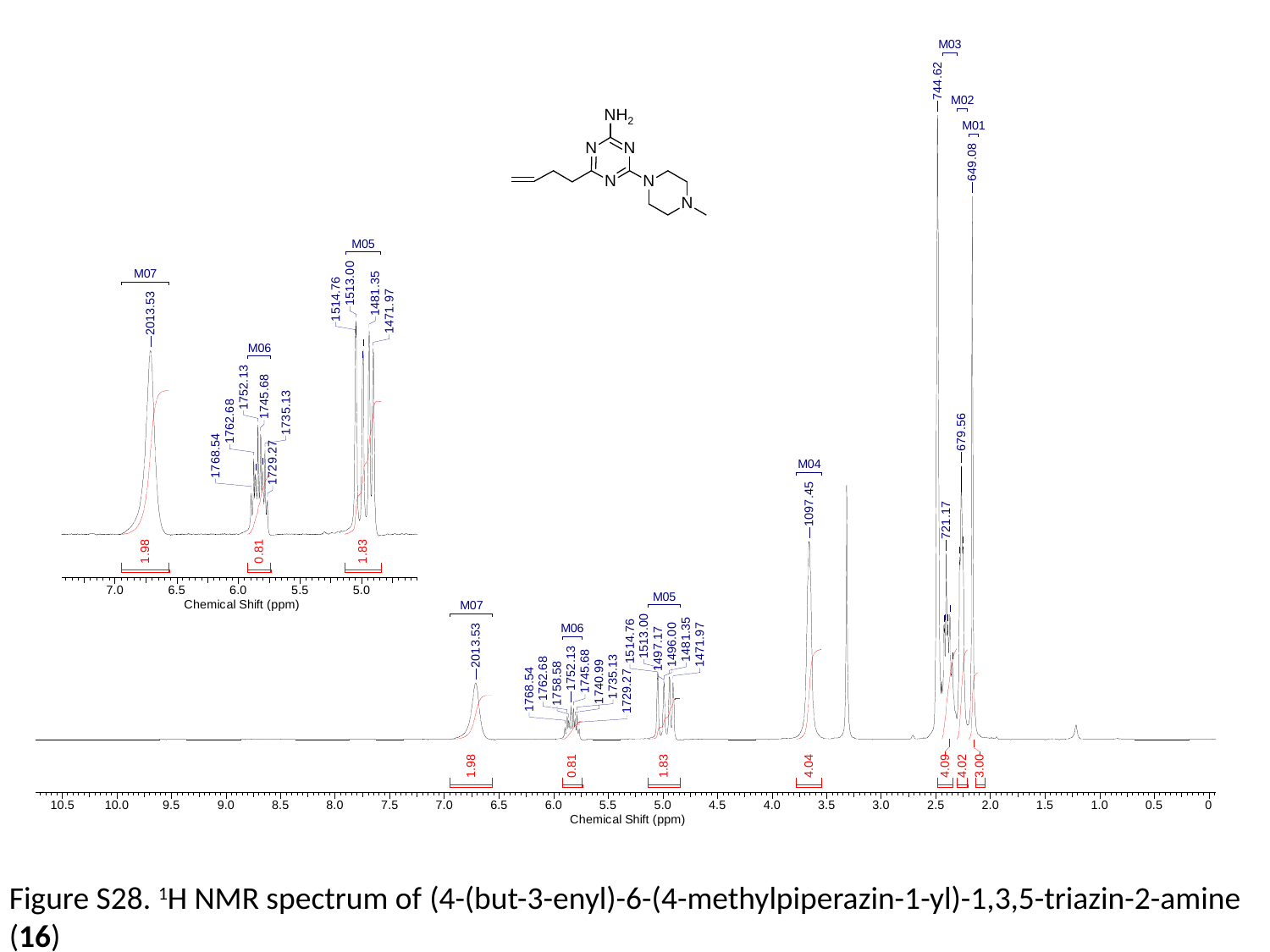

Figure S28. 1H NMR spectrum of (4-(but-3-enyl)-6-(4-methylpiperazin-1-yl)-1,3,5-triazin-2-amine (16)

## Slide 30
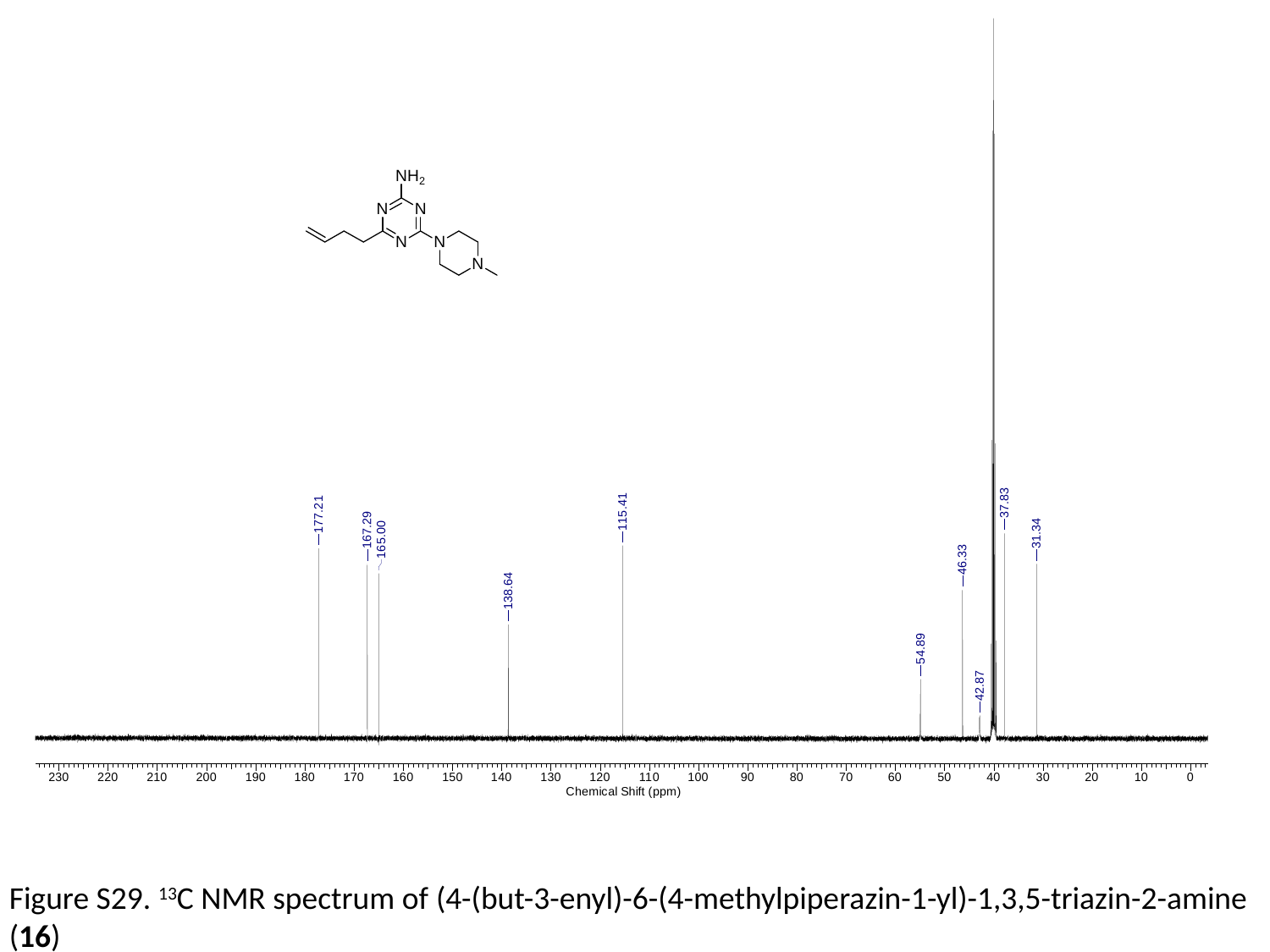

Figure S29. 13C NMR spectrum of (4-(but-3-enyl)-6-(4-methylpiperazin-1-yl)-1,3,5-triazin-2-amine (16)

## Slide 31
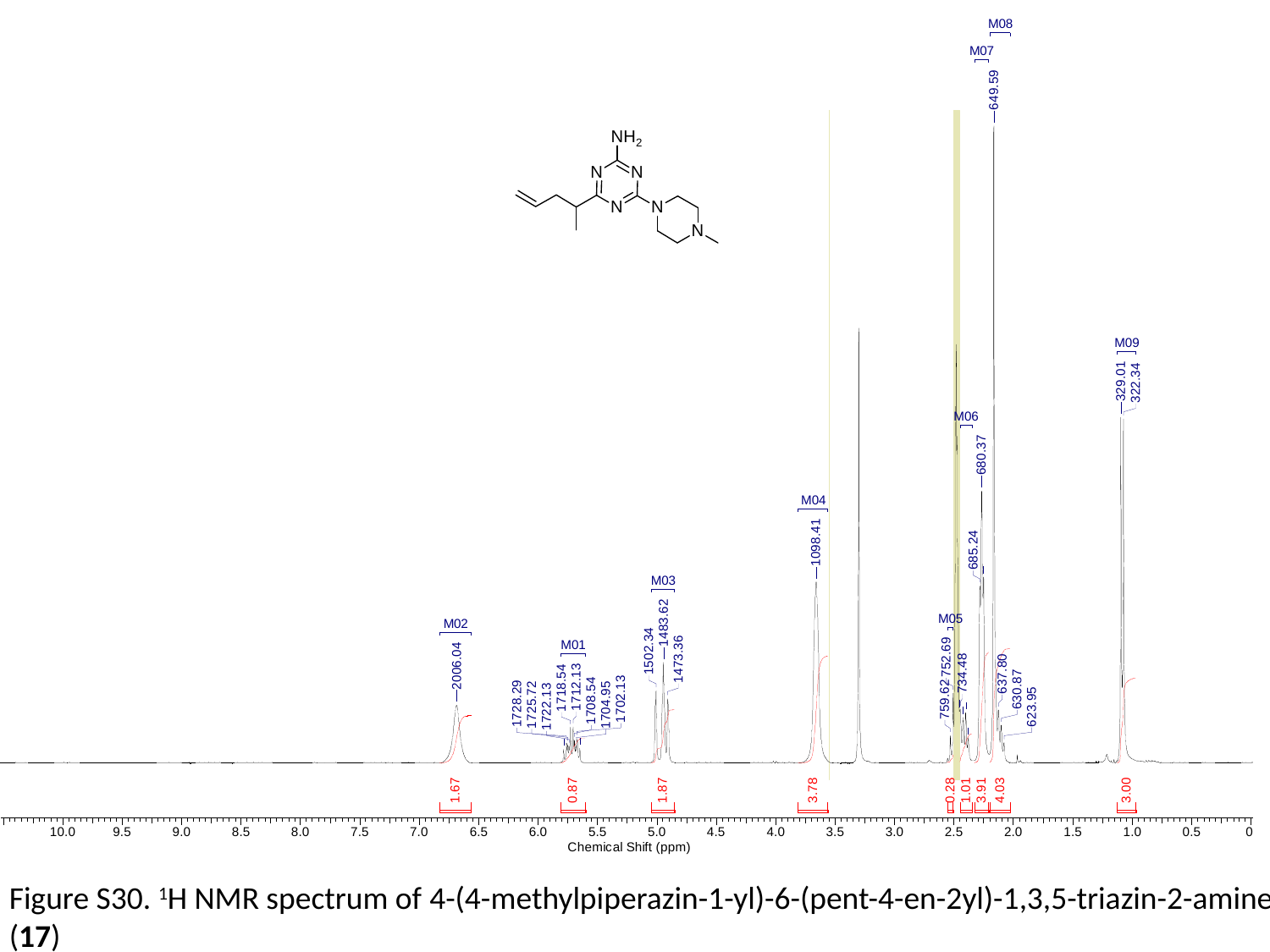

Figure S30. 1H NMR spectrum of 4-(4-methylpiperazin-1-yl)-6-(pent-4-en-2yl)-1,3,5-triazin-2-amine (17)

## Slide 32
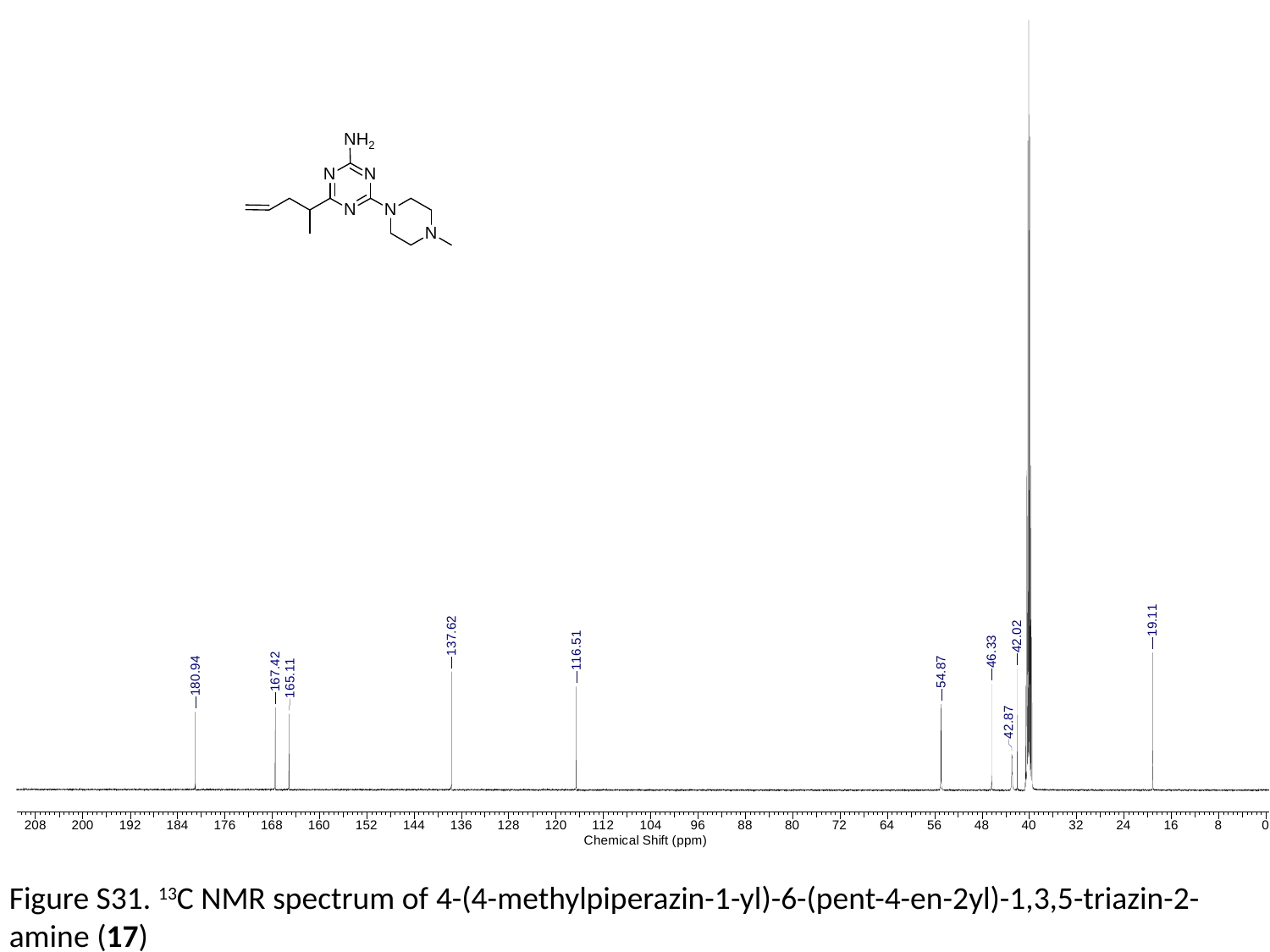

Figure S31. 13C NMR spectrum of 4-(4-methylpiperazin-1-yl)-6-(pent-4-en-2yl)-1,3,5-triazin-2-amine (17)

## Slide 33
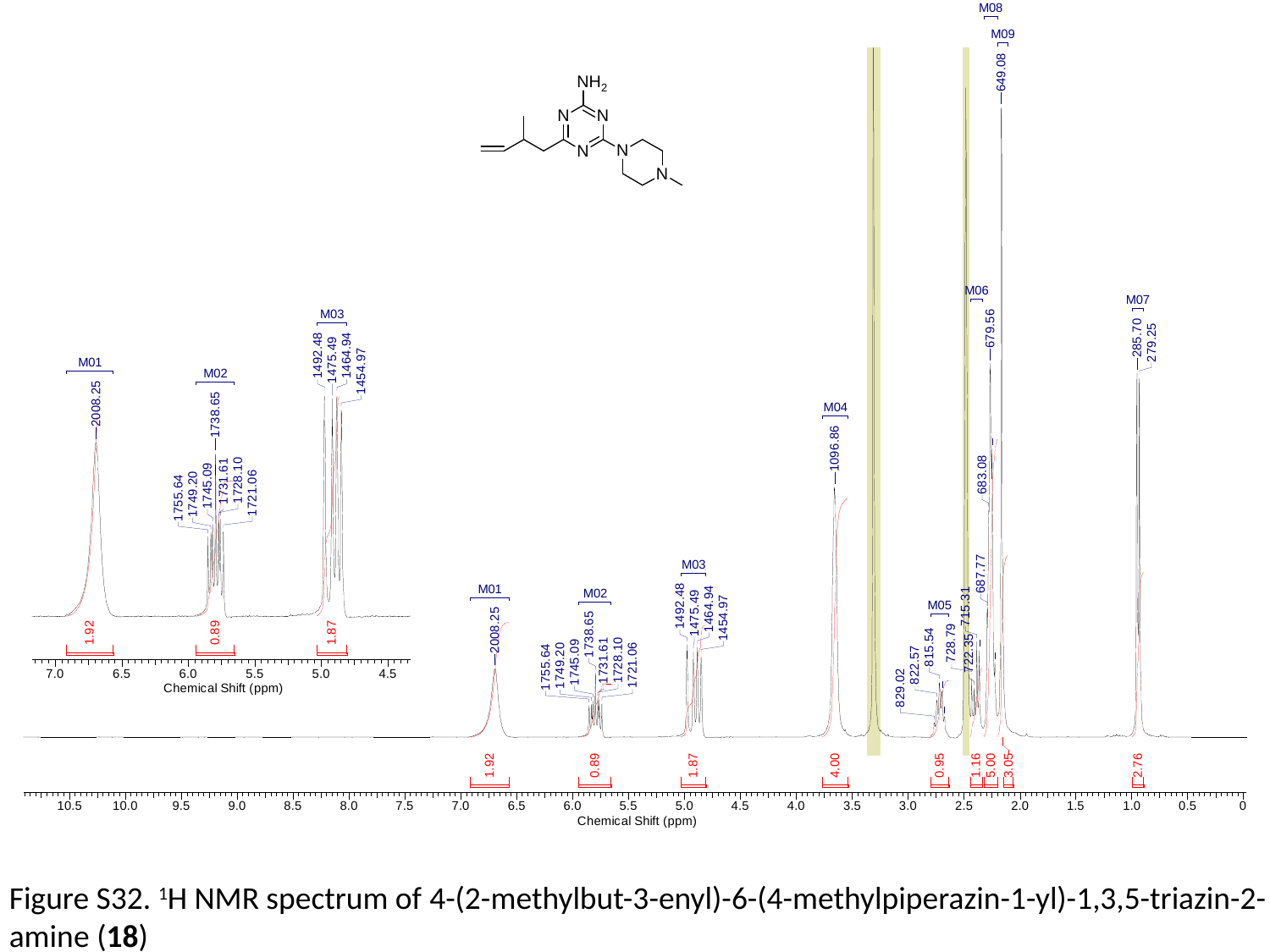

Figure S32. 1H NMR spectrum of 4-(2-methylbut-3-enyl)-6-(4-methylpiperazin-1-yl)-1,3,5-triazin-2-amine (18)

## Slide 34
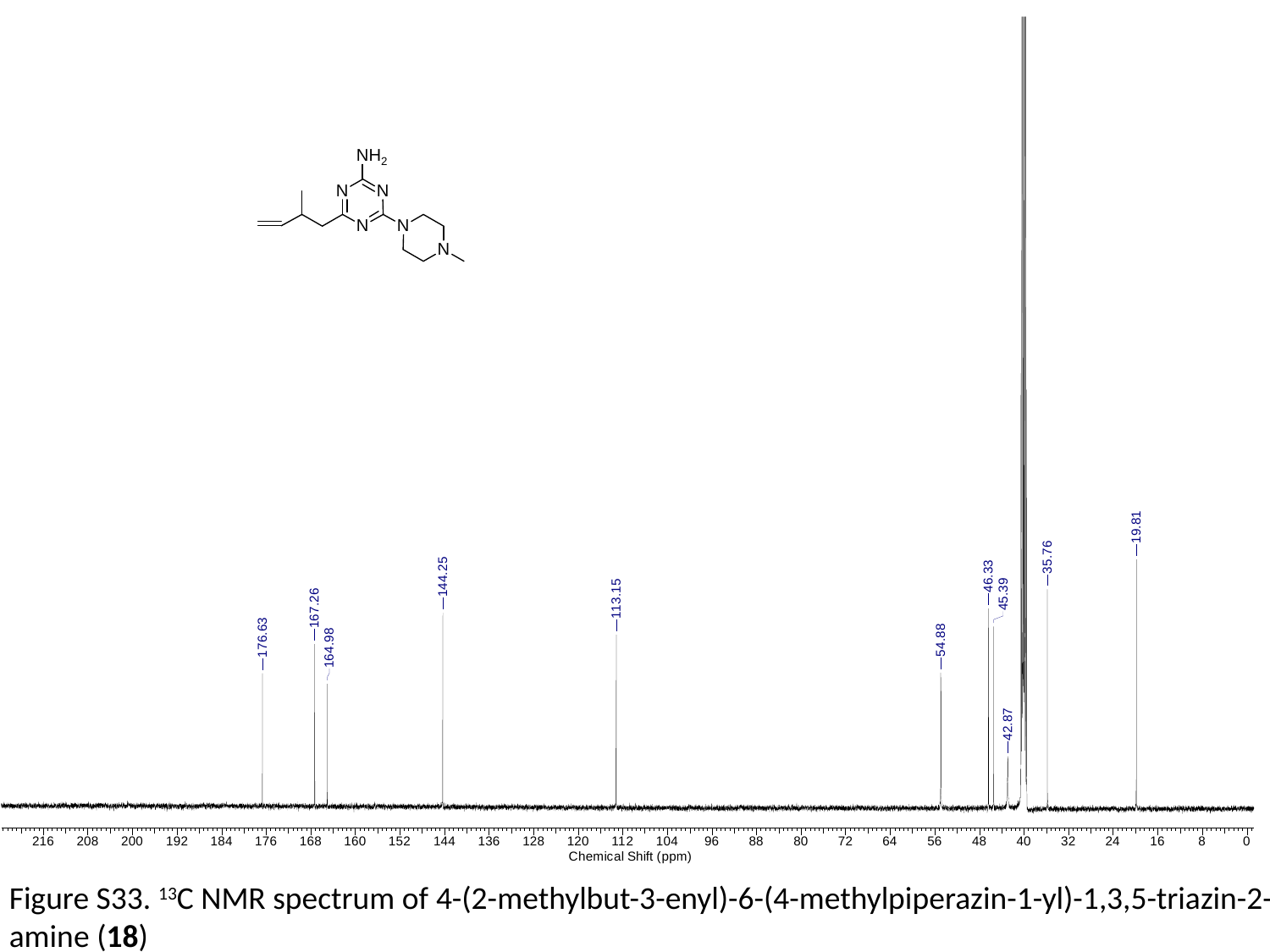

Figure S33. 13C NMR spectrum of 4-(2-methylbut-3-enyl)-6-(4-methylpiperazin-1-yl)-1,3,5-triazin-2-amine (18)

## Slide 35
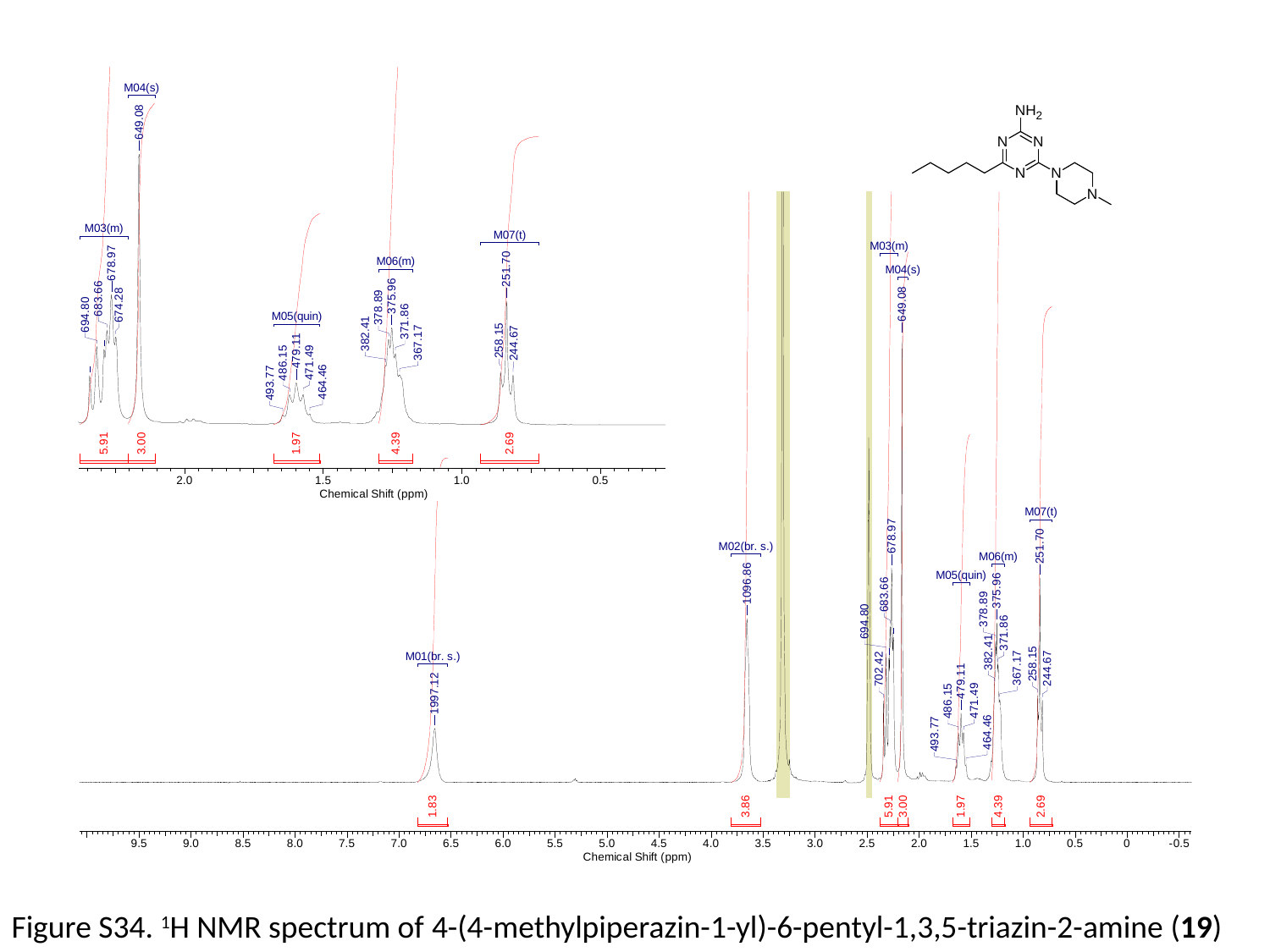

Figure S34. 1H NMR spectrum of 4-(4-methylpiperazin-1-yl)-6-pentyl-1,3,5-triazin-2-amine (19)

## Slide 36
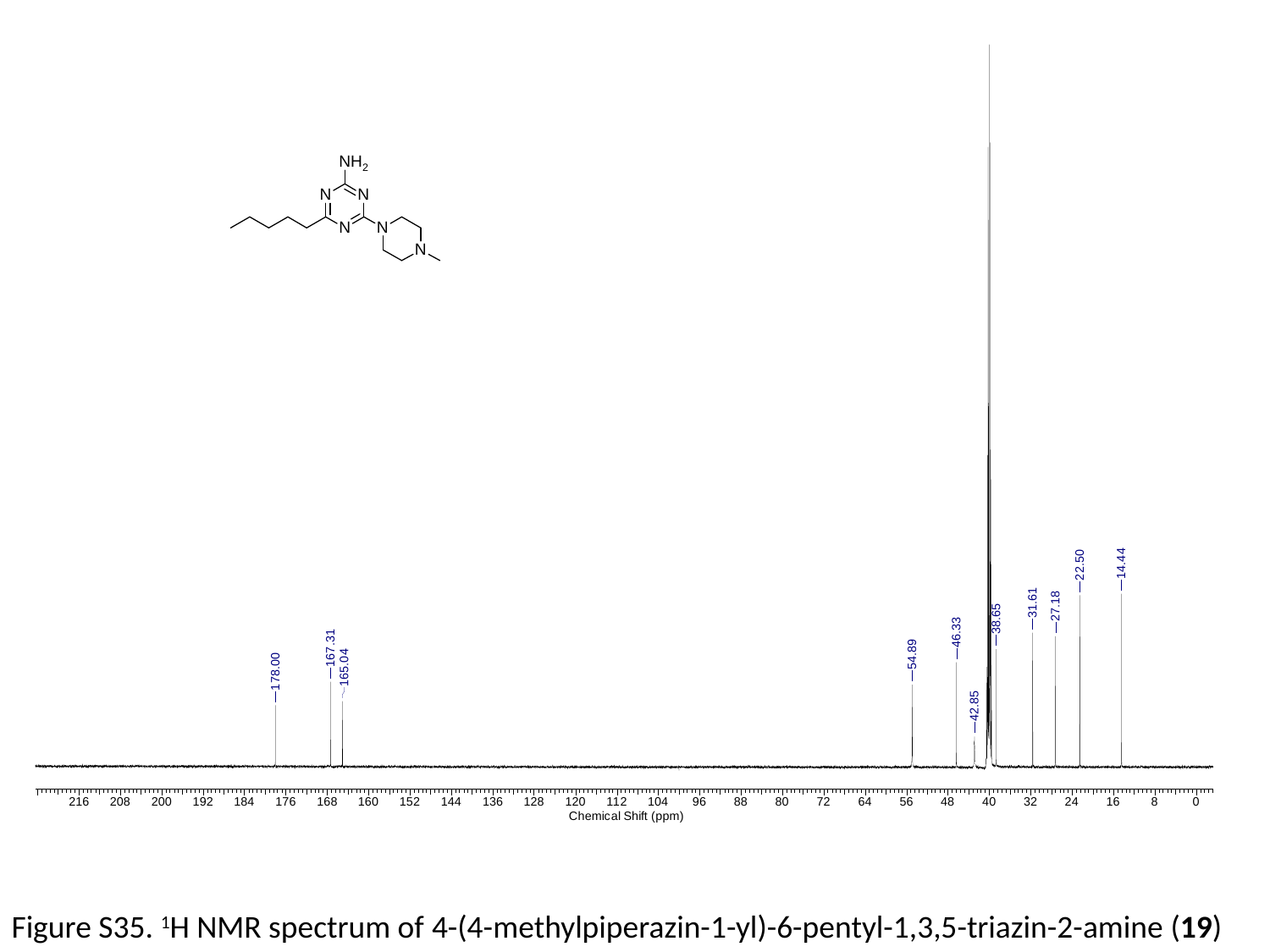

Figure S35. 1H NMR spectrum of 4-(4-methylpiperazin-1-yl)-6-pentyl-1,3,5-triazin-2-amine (19)

## Slide 37
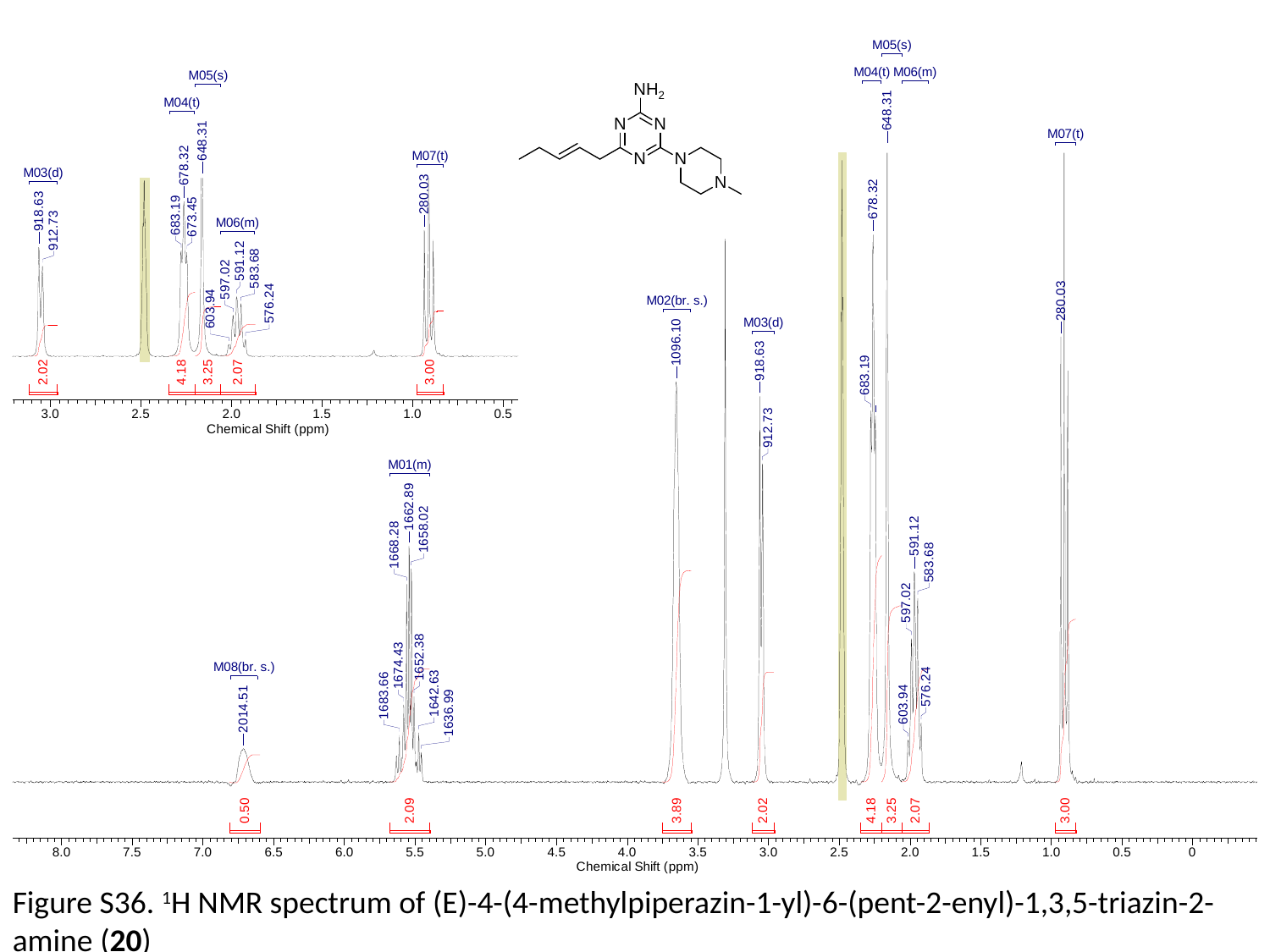

Figure S36. 1H NMR spectrum of (E)-4-(4-methylpiperazin-1-yl)-6-(pent-2-enyl)-1,3,5-triazin-2-amine (20)

## Slide 38
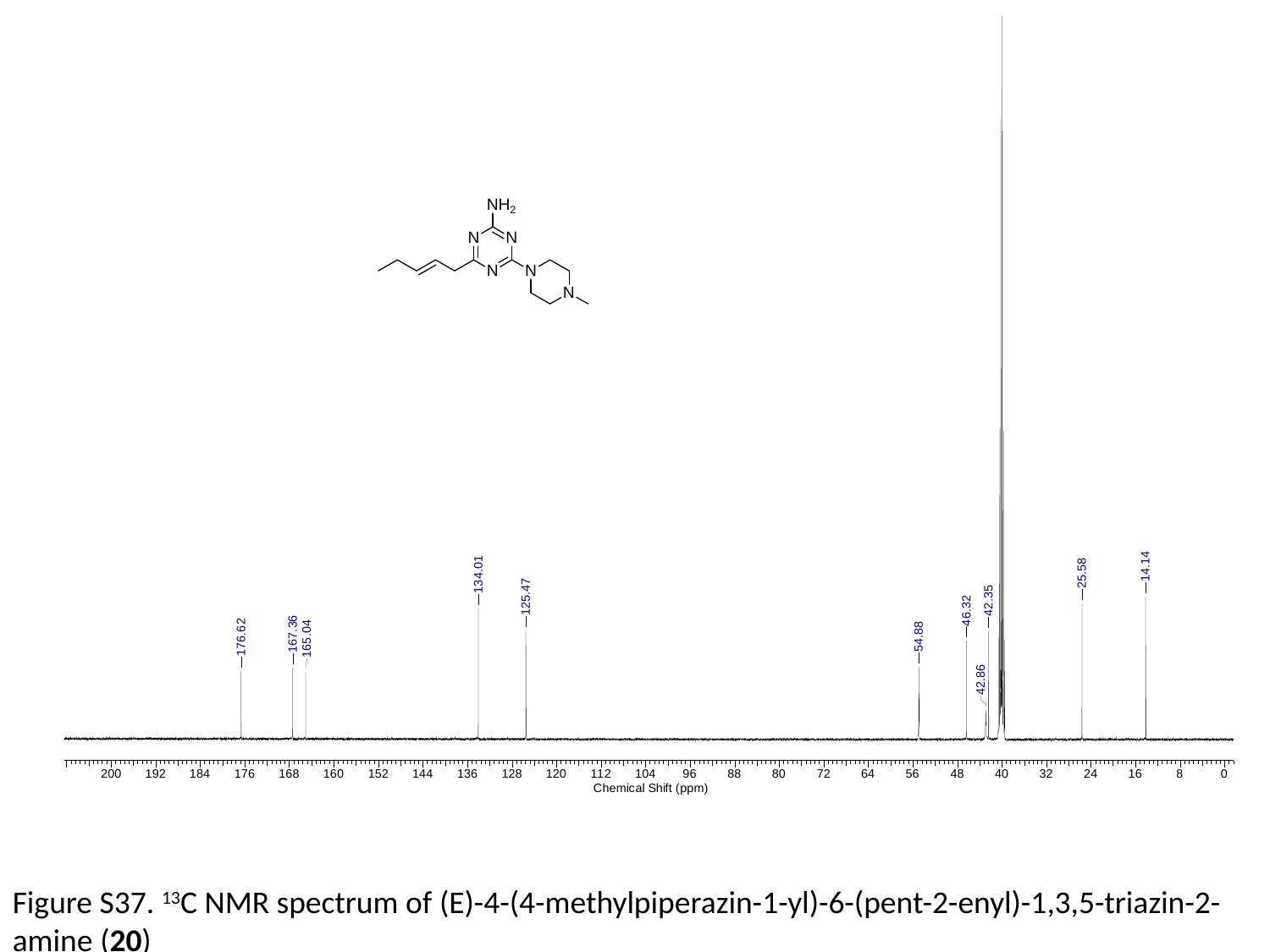

Figure S37. 13C NMR spectrum of (E)-4-(4-methylpiperazin-1-yl)-6-(pent-2-enyl)-1,3,5-triazin-2-amine (20)
